# Qualitative assessment of family caregiver-centered neonatal education program in Karnataka, India

**DOI:** 10.1371/journal.pgph.0000524

**Published:** 2023-02-14

**Authors:** Shirley D. Yan, Sahana S.D., Meghna Desai, Megan Marx Delaney, Lauren Bobanski, N. Rajkumar, Seema Murthy, Natalie Henrich

**Affiliations:** 1 Noora Health, San Francisco, California, United States of America; 2 Aurora Health Innovations, Bengaluru, India; 3 Ariadne Labs, Harvard T.H. Chan School of Public Health/Brigham and Women’s Hospital, Boston, Massachusetts, United States of America; 4 Directorate of Health & Family Welfare Services, Bangalore, Karnataka, India; The University of Texas Health Science Center at Houston School of Public Health - San Antonio Campus, UNITED STATES

## Abstract

Globally 2.5 million newborns die every year before they reach the age of one month; the majority of these deaths occur in low- and middle-income countries. Among other factors, inadequate knowledge and skills to take care of newborns contribute to these deaths. To fill this gap, training patients and family members on the behaviors needed to improve essential newborn care practices at home is a promising opportunity. One program that aims to do this is the Care Companion Program (CCP) which provides in-hospital, skills-based training on care of mothers and newborns to families. This study uses semi-structured interviews to understand how and why knowledge and behaviors of maternal and newborn care behaviors change (or don’t change) as a result of CCP sessions and participants’ perception of the impact of CCP on change. Interviews focused on knowledge and behaviors around key neonatal and newborn topics and health seeking behaviors for health complications. Forty-two in-depth interviews were conducted among families with recently-delivered babies at their homes from four districts in Karnataka, India. Respondents have a positive perception about CCP, found training useful and appreciated other family members presence during the training. CCP increased knowledge and awareness and provided critical details to key behaviors like breastfeeding. Respondents were more likely to be receptive toward details on already known topics, like hand washing before touching the baby. Awareness increased on newly learned behaviors, like skin-to-skin care, which don’t conflict with cultural norms. The CCP did not influence nonrestrictive maternal diet as much, which cultural norms heavily influence. In-hospital family caregiver education programs, like CCP, can positively influence key neonatal behaviors by imparting knowledge and key skills. However, the effect is not universal across health behaviors.

## Background

Currently 2.5 million deaths occur in the first month of neonatal life worldwide [1]. The majority (90%) of these deaths occur in LMICs due to infections, complications of preterm birth, and intrapartum related complications [2, 3]. In India, about four-fifths of these deaths occur within the first week of delivery of the more than 750,000 neonates die annually in India [4–6]. Causes of death in the late newborn period include bacterial infections, pneumonia and infection of the umbilical cord stump, which hand washing, exclusive breastfeeding and dry umbilical cord care can help prevent, respectively [7]. Several key behaviors, such as exclusive breastfeeding, skin-to-skin contact, dry umbilical cord care, unrestrictive maternal diet, and hand washing with soap, can reduce health complications and adverse health outcomes [8–12]. Initiation of early and exclusive breastfeeding in the first six months of neonatal life can prevent 20% of newborn deaths especially due to sepsis, pneumonia, tetanus, and diarrhea [8]. Additionally, maintaining dry cord care and maintaining hand hygiene can prevent neonatal deaths by reducing infection [9, 10]. During the neonatal period (first 28 days of life) and after, practice of key health behaviors are critical.

Globally, there is value to address knowledge gaps around postnatal behaviors. Though it is well documented in the literature that the previously mentioned key behaviors contribute to the prevention of poor neonatal and maternal health outcomes post-delivery, knowledge and practice of these behaviors vary widely. A Nepal based study found that while postnatal mothers knew about early breastfeeding, many of them had less knowledge on hand washing and recognition of danger signs [11]. In a cross sectional study in Nepal among recently delivered mothers, 48.7% had inadequate knowledge and 33.8% inadequate behavior for newborn care [13]. In a qualitative study in Uganda among mothers of low-birthweight babies, danger sign recognition, initiation and exclusivity of breastfeeding, and maintenance of these behaviors could be improved [14]. Maternal and neonatal care practices have been shown to vary across India but are largely influenced by religion, cultural practices beliefs, socioeconomic status, and decision of family members [15, 16]. In Karnataka, a state located in Southern India, persisting cultural beliefs and practices have influenced families to withhold nutritious foods for mothers post-delivery, apply various substances to the umbilical cord stump and eyes of the newborn, and give prelacteal feeds instead of exclusive breastfeeding [17]. Pre-discharge education on post-discharge behaviors must include the whole family to practice newborn care to reduce the risks of neonatal mortality [18, 19].

Behavior change communication has shown demonstrable impact on health behavior change and improved health outcomes. In an intervention in which frontline health workers provided counseling, posters, pamphlets, and demonstration on postnatal care, the intervention group reported reduction of potentially harmful behaviors, including withholding nutritious foods considered "hot" or "cold"; application of various substances to the umbilical stump and eyes of the newborn and giving prelacteal feeds; significant improvement in breastfeeding; and increase in knowledge on skin to skin care, identification, and management of danger signs in babies [20]. Evidence from a behavior change communication delivered through rural Indian self-help groups showed a 5–11 percentage point increase for positive antenatal, natal, and postnatal behaviors (e.g. breastfeeding, skin-to-skin care, and cord care) [21]. In addition to community based programs, hospital based programs focused on delivering neonatal information to parents have positively changed health behaviors and resulted in improved maternal outcomes (e.g. knowledge, self-efficacy, behavior, feeling supported, and attachment) and newborn health (e.g. practice of preventive behaviors, immunization, morbidity, and mortality) outcomes primarily due to uptake of knowledge for exclusive breastfeeding [19]. Based on a cross sectional analysis of 13,730 families who recently delivered in three Indian states, it is clear that newborn care topics are taught within facilities, but consistency on coverage across topics differ; for example breastfeeding is mentioned the most (26.2% of participants) and dry umbilical cord care the least (0.3%) [18].

While parent and family caregiver education is promoted throughout the literature, it remains unclear the impact of this intervention on the knowledge and behaviors of trained families [19, 22]. This qualitative study aims to understand how trained families experience one such neonatal education program focused on mothers and family caregivers: the neonatal Care Companion Program (CCP). Additionally, this study aims to assess how and why knowledge and behaviors of maternal and newborn care behaviors changes as a result of the CCP sessions, and participants’ perception of the impact of CCP on their change.

### Program description: Noora health’s Care Companion Program

Noora Health along with its associate organizations supports implementation of Neonatal Care Companion Program (CCP), a family caregiver education program that teaches families knowledge and skills pre-discharge, with the aim to reduce complications and hospital readmissions. With technical support from Noora Health and its affiliate partners, district hospital nurses and counselors teach neonatal topics pre-discharge to new mothers and their family members in wards and waiting rooms postpartum before hospital discharge about caring for themselves and their newborns. The postnatal Care Companion Program (CCP) sessions teach families key preventive behaviors (non-restrictive diet for mothers, exclusive breastfeeding, skin-to-skin care, dry umbilical cord care, hand hygiene, danger sign recognition for the mother and baby, and danger sign recognition for the baby and recently delivered mother) which is implemented at district level hospitals in six states of India (Punjab, Karnataka, Madhya Pradesh, Maharashtra, Telangana, Andhra Pradesh) (Table 1). As of April 2022, CCP programs have been implemented in 321 hospitals and health facilities across India in maternal and child health, oncology, cardiology, and inpatient condition areas.

**Table 1:** Key behaviors definitions and corresponding information taught in CCP sessions.

Behavior	Definition	CCP recommended behavior and how they are taught
Breastfeeding	Exclusive breastfeeding for the first six months of life, unless prescribed by doctor	• Exclusive breastfeeding newborns up to six months and only feeding formula if recommended by a doctor • No complementary feeding with cow’s milk, honey, gripe water (typically given to infants who are colicky or have other gastrointestinal issues), sugar water and any other things • Complete mother’s diet to help with breast milk production • In CCP session, trainer teaches behavior importance, how to troubleshoot, and taught with doll to demonstrate baby positioning
Dry Cord Care	No application of any substance on the baby’s umbilical cord care until it falls off, unless prescribed by a doctor	• No touching the cord unnecessarily, no pulling off the cord, and no applying anything (powder/oil/cream) to the cord before it falls off or it fully heals • The cord area should be kept dry and clean at all times and the cord will fall off by itself in 1–2 weeks • In CCP session, trainer teaches warning sign recognition and health-seeking behavior
Postpartum maternal Diet	Non-restrictive diet for food or liquids for postpartum mothers	• Maternal diet should include vegetables, fruits, and dairy, and meats if the mother is non-vegetarian • No restrictions of any food nor the quantity of food. In particular, "cold" foods do not have to be avoided and drink at least 8–10 glasses (2–3 liters) of water every day • In CCP session, trainer teaches what all mothers can eat postpartum
Hand Washing	Hand washing with water and soap before touching or feeding baby, after going to the bathroom, and before eating	• Wash hands with soap for 30 seconds including the front and back of their hands, between fingers, and under nails. • Wash hands before touching or feeding the baby and after going to the bathroom. • In CCP session, trainer teaches handwashing importance and demonstration
Skin-to-skin contact	Performs skin-to-skin contact with baby when baby is cold, for low-birthweight babies, and for general bonding	• Skin-to-skin care can be performed by putting the bare baby (except the diaper, cap and socks) on the bare chest of the father/mother to provide steady warmth. CCP promotes skin-to-skin care for all babies and not just underweight or preterm babies. • It should be practiced regularly, not only if you think the baby is cold • In CCP session, trainer teaches importance and demonstrates how to perform skin-to-skin contact
Warning sign identification	Identify signs of diarrheal disease, jaundice, signs of infection, hypothermia	• Recognition of danger signs in newborns (such as fever and hypothermia, lethargy, poor feeding, and blood in stools) and how to manage symptoms • In CCP session, trainer teaches warning signs to look out for
Care-seeking behavior	Upon recognition of warning signs, families seek medical care at hospitals.	• Seek medical attention upon recognition of danger signs in newborns • In CCP session, trainer teaches when to see care at hospital

Master trainers (nurses or counsellors) generally facilitate sessions in hospital hallways or wards. Noora Health supports these master trainers through training of trainers (two-three days), booster trainings (one-two days) and ongoing coaching which emphasize soft skills and tools master trainers can use to facilitate a session. An ideal CCP session involves use of flipcharts (S1 Text), visuals, and supporting text for the trainers to talk through the topics over 20–30 minutes (see flipchart example in the Annexure). Props such as a doll are used to demonstrate key skills, such as positioning for breastfeeding, how to perform skin to skin contact, and warning sign identification for jaundice. If there are televisions in the hospital, videos are used as part of the session to teach key preventive behaviors (i.e. dry cord care, skin-to-skin contact, exclusive breastfeeding, hand washing, and postpartum maternal diet; the same content that trainers cover). During a session, trainers should cover all topics, introduce the Whatsapp-based service, and encourage participants to ask questions as necessary. The Whatsapp service delivers information and behavior reinforcement messages and videos, covering the same content delivered in the CCP session. To enroll, the trainer asks the families to give a missed call to the postnatal Whatsapp number, which will then register the phone number into the service. At the end of the session, trainers distribute paper handouts to families that highlight the main topics taught in CCP. Full list of tools are shown in the annexure. Classes are conducted in groups, which has advantages over one-on-one counseling sessions that health professionals typically conduct with patients at their bedside [23–25]. These tools and overall approach for the sessions were created using a human-centered process and adult learning principles. Medical content is written and reviewed by an internal team of medically trained doctors, following from WHO and Ministry of Health, India literature and guidelines on what behaviors are linked to preventable complications. State Departments of Health and District Surgeons and Medical Officers provide final sign off to implement neonatal CCP sessions on a regular basis. The neonatal CCP program is an integrated component of the public health system in Punjab, Karnataka, Madhya Pradesh, Maharashtra, Andhra Pradesh, and Telangana implemented by the public health infrastructure. On average, sessions are run one-three times in a week to maximize the number of participants who receive the training, yet limit the burden on health care staff in delivering the program. Whether CCP sessions are implemented exactly as idealized depends on availability of infrastructure (e.g. televisions, printing challenges for handout distribution), hospital prioritization to run sessions (e.g. allocate time for nurses, hospital leadership’s interest in sessions), and individual nurse factors (e.g. nurse motivation).

### Methodology

#### Study setting

This study was set in Karnataka, which had an infant mortality rate of 27 per 1000 live births in 2015–2016 (slightly below the national average of 32.3), down from 43 since 2005–6 [26]. Mothers and family members were recruited to participate in this study if they attended CCP sessions in one of the four district hospitals (large tertiary hospitals) in Chikkaballapur, Ramanagara, Dharwad and Bagalkot, Karnataka and who had completed a short survey 28-day post-discharge survey as part of a separate on-going study covered under a separate IRB [18]. The 15-minute post-discharge survey covered questions on knowledge and behavior of key behaviors; and self-report of complications or readmissions, which will be reported separately. This qualitative research provides insight into participants’ perception of the CCP sessions, how and why families performed behaviors, and explores facilitators and barriers to change. At the time of planning for this qualitative study (July 2019), the implementation and research team looked at the eight study sites from the larger neonatal study and selected facilities based on delivery load in the last month, CCP attendance in the last month, geographic location in Karnataka, number of sessions run each month, and quality of sessions. Recently delivered mothers participated in the survey if they were 18 years or older, delivered within the hospital, and neither the mother nor newborn died before discharge. These participants would have attended sessions within selected district hospitals within January-February 2020.

#### Sampling strategy and inclusion criteria

Based on the post-discharge survey responses, study team members purposively recruited interview participants based on whether newborn complications were reported, given this is a main health outcome CCP aims to impact. The goal was to recruit equal numbers of mothers and families with and without reported newborn complications. Only individuals who spoke Kannada and/or Hindi were recruited. Mothers and family members who did not complete the 28-day post discharge survey were not included. The sampling targets were set at 40 participants total (20 family caregivers and 20 recently delivered mothers with half the participants reporting baby complications) due to expected saturation in responses.

#### Data collection instruments

The research team developed separate semi-structured interview guides for mothers and family members with questions aimed at understanding how the CCP influenced behaviors, knowledge, and confidence among women and family members. Interview questions covered the following themes: attitudes, beliefs, and norms around newborn and maternal care practices (including perceptions, benefits, risks, challenges, facilitators), and acceptability and feasibility of the CCP. Particular focus was on specific health behaviors (i.e. exclusive breastfeeding, dry umbilical cord care until it falls off, handwashing before feeding and touching the baby, skin-to-skin care on a routine basis and if the baby is cold, unrestricted maternal diet for women, and danger sign recognition during the neonatal period), and experience for women and families who experienced baby complications (see Table 1). Among participants who reported a complication with their newborn, follow up questions around health seeking behavior and follow up actions were explored.

#### Researcher team composition

A third-party external agency conducted the semi-structured interviews; four of the interviewers were female and one male. This external agency was trained on the CCP program, interview guide, and interview techniques. Staff had Kannada, Hindi, and English language capacities and came from social science and public health training. After an initial recruitment call to confirm participant interest, the interviewers would call to schedule the interview. Interviewers conducted the interviews within the household where other household members would often observe the interview. Logistically, it was challenging to enforce privacy with the new mother or family caregiver given the newborn needs and the local cultural context. An interviewer and note-taker pair conducted the interview at the participants’ home from February-March 2020 (before the COVID-19 pandemic), audio-recorded, and transcribed and translated into English. The research team read transcripts for quality and de-identified transcripts.

The research tools and protocol were first piloted by members of the research team (who were not involved in eventual data collection), and the interview guide was refined and simplified. After the third-party agency was trained, they piloted the interview guides as well and received interviewing feedback. The research team and external agency iterated on the interview guide during this period, refining language, probing questions, and question flow, based off of initial interview responses (S2, S3 Texts). Results of this qualitative research are reported against Standards Reporting for Qualitative Research guidelines [27]. Additional information regarding the ethical, cultural, and scientific considerations specific to inclusivity in global research is included in the S1 Checklist.

#### Ethical review

This study received IRB clearances from Harvard University in Boston, USA and ACE Ethics Committee in Karnataka, India. All participants underwent a written consent process for the interview and audio recording and signed (or provided thumbprints if they couldn’t read) in their local language.

#### Analysis process

Research team members, who did not conduct interviews, created a codebook after reviewing five de-identified interviews through a mix of inductive and deductive coding. A doer/non-doer analysis framework informed codebook development to understand why people performed or did not perform a specific behavior [28]. The finalized codebook included themes around barriers and facilitators for key behaviors, health seeking behavior in the event of neonatal complications, and impact from CCP sessions (S4 Text). The same researchers then coded the interviews using Dedoose online software and reviewed each other’s codes [29]. They kept in touch with the interview team as questions arose. The analysis is based on the key behaviors according to the doer/non-doer framework, to understand barriers, facilitators, and context for the key behaviors and associated impact of the CCP program.

## Results

The total sample size for this analysis is 42 respondents (23 mothers and 19 family caregivers), all from different families. Interviews lasted between 20–80 minutes. Table 2 describes the demographics of respondents and the profile of the family’s newborn, collected through the 28-day post discharge survey. Of note, the majority of the family caregivers interviewed were grandmothers to the newborn.

**Table 2:** Demographic characteristics of participants.

	N = 42
Respondent Type	
Mother	23 (54.8%)
Family Caregiver (e.g. Maternal and paternal grandparents, paternal aunt)	19 (45.2%)
Maternal Characteristics	
Maternal Age (mean age and range)	23.4 (18, 31)
Family owns smart phone	35 (83.3%)
Birth Characteristics	
Singleton birth	42 (100%)
Primiparous	26 (61.9%)
Caesarean section	26 (61.9%)
Baby sex: female	20 (47.6%)
Special Newborn Care Unit admission	8 (19.0%)
Reported Baby Complications post discharge	23 (52.8%)

### Overall perceptions of the Neonatal Care Companion Program (CCP)

Our findings show that respondents have a positive perception of the neonatal CCP sessions. More than half of respondents (n = 30) said they found the training useful (18 caretakers, 12 family members) and 32 respondents said they would recommend the CCP to a friend (18 caretakers, 14 family members). Respondents appreciated the knowledge and skills taught in the class:

*[The group session] was very useful for us. We came to know how we need to be when the baby is around, how to protect the baby, we have to go to hospital and consult doctors if there is any problem with the baby. They explained all these things to us. (Mother, complication group)*

*Interviewer: Is there any difference in taking care of those babies and this baby?*

*Respondent 1: I am taking care of this baby very much better than the other babies.*

*Interviewer: How is that?*

*Respondent 1: Because I came to know about baby’s care from the group class. I was unaware of all those things earlier and I was not so worried about all those things before. Now I know about baby’s care very well after attending the group class. (Family Caregiver, no complication group)*

Mothers (n = 19) said it was helpful to have family members present during CCP and it made them feel supported.

*I felt good to have [my mother and my brother] with me. All the things which we have been taught in the class are useful for me as well as for my family members as they can look after the other babies of our family in the future. I felt this for having them in the group class. (Mother, no complication group)*

Twelve respondents that expressed worry or concerns before the birth of their baby (or from the CCP session itself) said that knowledge and skills learned in the CCP helped alleviate some of their worry and concern.

*Respondent: I was not worried about anything but I got scared after hearing the nurse when they spoke about things that could go wrong for a baby. So I started to observe the baby’s urine color and umbilical cord. I was a little scared about it.*

*Interviewer: Did the group class help you to overcome your fear?*

*Respondent: Yes, they send the information through messages and after reading that information I don’t feel scared anymore. (Mother, no complication group)*

*We just felt that we have to follow all the instructions that they told us in the group class. We would have never come to know all these things if they didn’t tell us all these things in the group class. We would have neglected the baby’s problems but now we will be very careful about all these things. They are spending so much money for us and for our good sake. So, I think we have to follow all the things that they have told us in the group class. We can plan according to the information that we received from the class. (Family Caregiver, complication group)*

#### Influence on knowledge, preventive behaviors, and confidence of newborns’ family caregivers

We analyzed five main, preventive behaviors taught in the CCP: exclusive breastfeeding, handwashing, skin to skin care, dry cord care, and non-restrictive mother postpartum diet. The data indicates that respondents come into the CCP with prior knowledge on newborn care behaviors and receive knowledge from multiple different sources including healthcare providers, family, friends, and media channels. Cultural norms and social beliefs also have an influence on individual newborn care behavior.

#### Exclusive breastfeeding

Breastfeeding is not a new behavior to most respondents. They have prior knowledge through their own personal experience (previous children, observing other women in family breastfeed), advice from health care workers (doctors and nurses at the hospital, Anganwadi workers) and advice from family and community members. Several (n = 7) respondents said that the CCP offered them new knowledge on breastfeeding including positioning, breastfeeding technique, using a pillow to help support the baby and the mother’s back, and burping.

*Normally, everyone would breastfeed however they liked…By doing it the way that they taught us it has been more convenient for the baby to drink breastmilk… and also it is “easier” for us…like this we can hold the baby(showing with hands how to cradle the baby in her arms before breastfeeding) put one hand on the baby’s back and this way (showing with hands making a ‘V’ with her finger on her chest indicating a scissor position to support the nipple). Doing this has made it easy for me to hold the baby and also it is more convenient for the baby this way to drink milk. (Mother, complication group)*

Respondents felt most confident in their ability to breastfeed when they are producing enough milk for their baby and when their baby is consuming enough breastmilk. In general, the most commonly mentioned barrier to breastfeeding is a lack of milk production.

All respondents said their babies were fed breastmilk (n = 42) but several respondents said that they also fed their newborn other foods, indicating suboptimal or nonexclusive breastfeeding. In general, respondents that experienced problems with milk production supplemented breast milk with other items (prior to six months of age) including cow’s milk and glucose water. One respondent that experienced difficulty producing breast milk was prescribed powder milk and provided it to her newborn. Respondents said that they breastfed with complementary feeding of animal milk (cow, sheep) (n = 5) or glucose water (n = 1) because the mother was not producing enough breast milk. Other items fed to newborns included almond paste (n = 1), ground dates and almonds mixed in with breast milk “for strength” (n = 1), Ghutti (herbal medicine for infants) and honey paste (n = 1), and honey (n = 2). Several respondents also fed their baby gripe water (not prescribed) (n = 7) and prescribed medicine (n = 2) to help with digestion and gas, and growth.

#### Handwashing

Most respondents have prior existing knowledge about good hand washing practice. Based on the data, handwashing seems to be a generally understood and accepted behavior (even prior to CCP attendance); it is a common behavior that is not unique to newborn care. The majority of respondents (n = 35) practiced regular hand washing *before* attending the CCP. Overall, mothers and family caregivers are aware of the importance of washing hands with soap. Respondents recognized the importance of handwashing (n = 39) and understood the benefits including being able to make the link between good handwashing practice and preventing infection.

Respondents attributed to CCP that they now regularly wash their hands with soap before touching their baby (n = 14), five respondents said that their family members also wash their hands now with soap before touching their baby and they are now more careful to wash hands more frequently with soap (n = 7). The CCP helped this group of respondents understand the benefits of using soap in addition to water when hand washing and helped respondents understand the importance of washing their hands prior to touching their newborn in order to help minimize spread of infection to their baby. One respondent stated that they do not follow the exact method of handwashing taught in the CCP but still wash their hands. Overall, respondents reported having regular access to water (in-house pipes, borewell, stored water) and soap which facilitates handwashing behavior. A few respondents also have access to hand sanitizer (n = 3). In general, the CCP influenced when respondents wash hands, including after using the bathroom, before eating and/or serving meals to family members, after doing housework, after coming home from doing outside work, and before touching their baby (including before breastfeeding and after handling a dirty diaper):

*Respondent: We brought a dettol (soap) to our home and we are using it to wash our hands. We wash our hands using it before touching the baby….*

*Interviewer: Is there any difference in the way of hand washing before and after listening to the group class?*

*Respondent: Yes, there is a difference.*

*Interviewer: How?*

*Respondent: I was not used to hand washing before listening to the class and started to wash after listening to them in the group class.*

*Interviewer: Can you tell me what you mean by you were not used to hand washing?*

*Respondent: I used to wash my hands only before and after having my meal earlier but now I keep washing my hands often because I have to carry my baby now. Everyone in our home washes their hands now.*

*Interviewer: So, everyone in your home washes their hands using dettol now?*

*Respondent: Yeah, everyone in our home uses the dettol in our home to wash their hands. (Mother, no complication group)*

*They said that we have to use dettol or soap to wash our hands. We have to wash our hands after coming home from outside or after touching anything. We have to wash our hands properly before touching the baby. We are following the same in our home. (Family Caregiver, no complication group)*

#### Skin-to-skin care

Overall, responses indicate that there is limited existing knowledge about skin-to-skin care for newborns. The data show that there aren’t any strong existing cultural and social beliefs around the practice, and family and community members are not offering advice around this behavior. Respondents’ knowledge of skin-to-skin care seems to be coming from the CCP and other healthcare workers.

More than half of respondents said that they do *not* practice skin to skin care (n = 25) although 29 respondents believe that there are benefits to skin-to-skin care including newborn weight gain, increased bonding between mother and newborn, and transfer of warmth. Many respondents (n = 33) said they learned about skin to skin from the CCP and some respondents (n = 6) could recall the use of a doll to demonstrate skin-to-skin care (“hugging”, “wrapping of doll”). However, there is variation among respondents in their understanding of how and when to perform skin-to-skin care. Responses regarding how skin-to-skin care should be performed varied and were sometimes contradictory to what was taught in the CCP. The following are examples of responses when respondents were asked how and when to perform skin-to-skin care:

- Baby should be bare
- Baby should be wrapped
- “Tie like a frog”
- Only mother and father can do skin-to-skin
- Any family member can do skin-to-skin
- Place baby on chest
- Place baby on stomach
- Family caregiver must bathe before practicing skin-to-skin
- Must bathe newborn before practicing skin-to-skin
- No direct skin contact with baby, must have cloth in between
- Only if baby is weak or cold
- Not if baby is weak/premature

A few respondents (n = 5) said that skin-to-skin care should only be done after the mother or family member bathes. Nineteen respondents said that skin-to-skin care should be practiced when the baby is cold or has a problem (when the newborn is “underweight” or “weak”, when the newborn has a “fever”).

*When the weight of the baby is low, they said to wrap the baby warm while putting him to sleep….They said to put the baby on chest and lay down while watching TV. They said to put the baby on the stomach with a bare body. (Mother, no complication group)*

A few respondents (n = 7) said they did not feel the need to practice skin-to-skin because their baby is healthy and others (n = 4) did not want to practice skin-to-skin because they felt their newborn was “too weak” (premature) and they were scared to carry their newborn because it could cause harm to the baby in some way.

*He is very small. I am afraid that I may drop him, so I have not given him ‘chest warmth.’ (Mother, no complication group)*

#### Cord care

Over half of respondents (n = 27) had prior knowledge around dry cord care practice. Sources of knowledge included advice around cord care from family members, influence of “elders”, prior personal experience, and information from healthcare workers (physicians, nurses, ASHA workers). Over half of respondents (n = 22) said they learned new information from the CCP or the CCP reinforced dry cord care knowledge.

*We didn’t do anything to the baby’s umbilical cord madam. It fell on the 6th day when we were still in hospital. We did not apply any oil or powder to it. They informed us in that class not to apply any powder as babies may get infections due to that which will lead to problems. Our baby did not face any such problems and it was all fine. (Mother, no complication group)*

The majority of respondents (n = 37) did not put anything on the cord or cord area and allowed the cord to naturally dry and fall off. Several of these respondents recalled that putting something on the cord could lead to infection (n = 7) and a few respondents recalled that dry cord care prevents infection and pus formation (n = 2). A few respondents (n = 5) put something onto the cord *before* it fell off (hot coconut oil, powder, castor oil, “boric powder”). Respondents that put something onto the cord before it fell off explained this was due to direct advice from a family member (“elders”, mother-in-law, sister). Thirteen respondents applied something (oil, powder) to the cord area *after* the cord fell off. Common reasons given for applying items to newborn cord or cord area include: to reduce pain, keep cord area dry, reduce redness, heal a cut, stop bleeding, or stop pus.

#### Mother postpartum diet restriction

Almost all respondents (n = 38) said that the mother restricted specific foods and/or liquids post-pregnancy, either for a specified (e.g. days or months) or unspecified amount of time. Thirty-three of these respondents could recall that the CCP recommended that the mother not restrict any food or liquid. However, six respondents recalled being told during the CCP to avoid certain foods (cold food, spicy food, salty food, oily food). In general, respondents offered contradictory answers to interview questions about maternal postpartum diet. When initially asked if they restrict any foods, respondents typically answer “No” and say that they follow what was taught in the CCP, but when probed about specific food items (fruits, spicy foods etc.) then the respondent said that the mother does restrict certain foods and liquids.

The majority of respondents restrict despite what they recall being instructed to do in the CCP. The data shows that a complex set of factors influence maternal postpartum diet restriction including individual beliefs, advice from family, advice from extended community, and cultural practices. Mothers seem to receive mixed messages and contradictory advice around diet from various sources. Key sources of advice include elders within their family such as their mother, grandmother, and mother-in-law. The elder women of the household often prepare food for the mother and therefore have control over what and when she eats; she is dependent on what her family provides her and has little choice.

*If the mother eats spicy food the baby gets dysentery and a burning sensation in his stomach. That is the reason I do not give her spicy food. I do not use green chilies. I use only red chili powder. I prepare food with very little quantity of chili powder. (Family Caregiver, complication group)*

There are strong held social beliefs about risks to the newborn and mother from consuming certain foods or drinking too much liquids. Consequently, respondents avoid eating certain foods and consuming too much liquid. The most common restricted foods and liquids include:

○ Foods and liquids that are believed to cause cold in mother and baby such as certain fruits, specific vegetables, and cold water
○ “Hard foods” such as certain grains and lentils, which are believed to make the baby’s stomach “hard” (not able to digest) and cause pain
○ Foods that are believed to cause “heat” such as spicy food
○ Foods that are believed to cause infection or “pus formation” after a c-section (rice)
○ Water: several participants restrict maternal consumption of water because they believe it will cause issues in the newborn’s small stomach, “twisting” in the stomach, and pain (n = 8). Out of these respondents a few said they restricted water or thinned milk because they had given birth to a male child (n = 3).

In general, participants seem to have regular access to meals with grain, fruits, vegetables, and water. A few respondents (n = 4) said that they do *not* restrict any foods or liquids post pregnancy. Three of these respondents directly attributed this to what they learned in the CCP.

*They told me to have more water all the time madam. We were not knowing the benefits of having more water. They told me that I can produce more milk for the baby if I eat good nutrition food. It’s the same with water as well. They explained to me that If I drink more water in the morning then I can produce more milk for the baby at the same time. If I don’t drink the water in the evening then I cannot produce the milk for the baby in the evening and in such situations, we may think that there is a problem in producing the milk and we rush to hospital. So, to avoid such things I have to eat good nutritious food and should drink more water all the time. I told the same thing to my mother as well. My mother is an old lady and they think that I will produce thin milk if I drink more water and that milk will not give any protein to the baby. That is the reason they don’t allow me to drink more water. They explained all the right things in the group session which helped me a lot. (Mother, complication group)*

Out of the three respondents, two said that food/liquid restriction was present for their first child but for the second baby there was no restriction due to what they learned in the CCP. Several respondents (n = 11) made the connection between a good maternal postpartum diet and breast milk production.

*Interviewer: Is your confidence about breastfeeding affected by the group class?*

*Respondent: My confidence is increased by the class.*

*Interviewer: How?*

*Respondent: They told us what to eat if we can’t produce more milk. They informed me to eat all the foods except cold foods, which can lead to not enough breastmilk. They even mentioned eating non-vegetarian food. That is what we are following at home. That is the reason I am confident that my baby is getting enough milk for him. (Mother, no complication group)*

#### Identifying danger or warning signs

We interviewed 23 respondents (13 mothers and 10 family members) that self-reported a post-discharge newborn complication in a follow-up survey conducted by Noora Health. Out of the 23 respondents identified using the survey, the most commonly reported complications were fever (n = 6) and cold/cough (n = 5), followed by cord infection (n = 4), jaundice (n = 2), rash (n = 2), infection from drinking “womb water” (n = 1), problems with urine production (n = 1), vomiting (n = 1), and gas (n = 1). From the analysis, we identified nine additional respondents from the non-complication stratum who mentioned that their newborn experienced some sort of problem (not originally mentioned in the 28-day post discharge survey). These respondents reported cord infection (n = 3), cold (n = 2), breathing problems (n = 1), rash (n = 1), stomach pain (n = 1), and discomfort when passing urine (n = 1). Two respondents (both family members) believed that the reason for their baby’s problem (fever, constipation, stomach pain) was due to maternal diet.

A few respondents (n = 4) said that the CCP influenced their knowledge and understanding of danger signs related to their newborn’s complications (3 fever, 1 cord infection). Four respondents said that the CCP influenced their symptom management and three respondents said that the CCP influenced their health seeking behavior. Several respondents (n = 4) said that the CCP had no influence on their ability to identify danger signs, symptom management, or health seeking behavior. In general, respondents either tried to manage symptoms at home with medicine from the pharmacy and/or they went to a clinic or hospital for treatment. There was no mention among respondents of utilizing a traditional healer.

*In the group class they told us not to apply any sort of oil to the umbilical cord as it will lead to infections and wounds may turn to worse. We have to take the baby to the doctor if there are any wounds near the umbilical cord. They asked us to always check the umbilical cord for any wounds but my baby had nothing as such. After one month our baby got a wound near the umbilical cord area. So, we took the baby to hospital and they prescribed ointment and powder to apply and we were applying that to the baby. (Mother, no complication group)*

Overall, respondents reported that they were able to recognize when their baby had a problem and understood the importance of seeking medical advice and treatment. The data indicate that health seeking behavior (taking baby to the clinic or hospital) is common among parents of newborns and that respondents believe they are able to recognize when something is wrong with their baby. In general, responses indicate that prior knowledge contributed to health seeking behavior. It is possible that the CCP reinforced certain knowledge around danger signs and behaviors (symptom management, health seeking behavior) but we cannot determine that with our current data.

At the start of the interview, all respondents (n = 42) were asked “*In the group meeting*, *the Nurses talked about a lot of things that could go wrong for a baby*. *How did hearing about these things make you feel*? *Why*?” Most respondents described danger signs taught. Fifteen respondents recalled being told about jaundice during the CCP and eight of these respondents recalled the symptoms of jaundice including yellowish discoloration of the eyes or body. Two respondents recalled that in the CCP the nurses said to take the baby to the hospital if they noticed breathing problems including if the baby started to “breathe heavy” and two respondents recalled that that in the CCP the nurses said to take the baby to the hospital if the “feet turn blue” and if the body turns blue. It is possible that respondents are able to recall the various danger signs discussed in the CCP but in this study the interview guide did not specifically explore each danger sign discussed in the CCP with each respondent.

## Discussion

Overall, participants perceive that CCP sessions have a positive impact on health behaviors though the extent varies across behaviors and type of participant. Mothers and family caregivers did report learning new information on new or already known behaviors, but the practice of these behaviors was not consistent. The practice of key behaviors varied, possibly due to nurse level factors, how new the information was to mothers and family caregivers, and whether behaviors stood in contrast with cultural and family advice. For example, steps and triggers for when to do skin-to-skin contact varied across participants, possibly because how some nurses promote skin-to-skin care for premature and low birthweight babies, compared to the CCP session which promotes skin-to-skin care regardless of premature or birthweight status. For other more common behaviors, such as exclusive breastfeeding or handwashing, which are already saturated with public health messaging, the value of the CCP session emerged when specific, new details were provided (e.g. how to troubleshoot breastfeeding challenges with hand positioning or when to wash one’s hands). A final potential explanation of whether the CCP changed skills depends on whether they align with cultural norms. For example, nonrestrictive maternal diet as taught in CCP conflicts with cultural norms, which may explain why the majority of mothers restricted food or liquids postpartum.

As for whether the CCP helped families identify warning signs, overall the data suggest that CCP information did help a few of the respondents who reported health complications and reinforced existing health seeking practices to go to a facility. Families either tried to manage symptoms with pharmacy-bought medicine or went to a health facility for treatment.

Based on the interviews, it was clear there were variations in the CCP session, like skin-to-skin care for postpartum maternal dietary habits. Based on the information from the interviews, we cannot determine why there is variation among respondents but it is possible that these topics may be inconsistently taught across CCP sites and/or respondents are receiving conflicting advice from healthcare workers (such as nurses and doctors at the hospital and frontline health workers).

These qualitative results completement a previous pilot evaluation of CCP in Punjab and Karnataka, which reported statistically significant improvements on dry cord care practice by 4%, skin-to-skin care by 78%, newborn complications reduced by 16%, mother complications by 12%, and newborn readmissions by 56% in the postintervention group as compared with preintervention group [30]. However, the practice of exclusive breastfeeding, unrestricted maternal diet, hand-hygiene and being instructed on warning signs were not statistically different. The health behaviors for which no statistically significant changes were found between the pre and post intervention group are those that either have a high ceiling effect or have strong pre-existing norms, as found by this study.

Given the findings from this qualitative study, there are several opportunities for improvement in the CCP program, most notably to include family members (e.g. what can mothers, fathers, grandmothers, or other family caregivers do) with specific messaging and address beliefs and practices directly (e.g. target grandmothers who prepare maternal foods and emphasize that all foods can be eaten). CCP sessions can be improved through greater program consistency across trainers, speaking directly to socio-cultural norms that may conflict with medical advice, and allowing for more interactive question-answer sessions. CCP sessions are similar to other parent focused education programs delivered before hospital discharge, in that it uses a multitude of tools and techniques and covers multiple topics. Like CCP, in a scoping review of parent focused education programs before hospital discharge, 61% focused on a single topic, almost all interventions delivered information verbally (93.5%), 20% through modeling, 17% through images, 11.7% using videos, and 11.7% in groups [19].

Findings demonstrated that other family members, especially grandmothers to the baby, play a significant role in postnatal care. This is consistent with Lunkenheimer’s research which identifies that families, friends, and neighbors are more likely to influence maternal diet than ASHA workers and contribute to restricting postpartum maternal diet in Bihar [31]. These family caregivers have particular influence and control over the mother’s diet, especially when they prepare the mother’s diet. Globally across Africa, Asia, and Latin America, Aubel argues the importance grandmothers have in maternal and child health given their experience and access to social networks for help beyond the household, although grandmothers offer advice, sometimes with outdated, unscientific information that is not in line with current medical recommendations [32, 33]. Interventions that include grandmothers especially as part of the target audience are key to changing behaviors and improving outcomes, given their influence on maternal and newborn practices. Given the mother’s unique position as both the “patient” and a “caregiver,” there is value in training the whole family so the burden doesn’t only fall onto the mother. It is estimated that girls and women contribute more than 70% of all caregiving hours globally [34]. Family caregiver training can help offset gendered care expectations for newborns, which often falls to the women of the family and involve male family members more.

The limitations of this study include the fact that respondents were not interviewed privately; limited location of the study; only inquiring about complications for the families who reported it; and the reliance on self-reported data. Though the intention was to interview mothers and family caregivers separately, practically this was not always possible due to the size of the house and the presence of others in the household. This invariably could have influenced the answers that participants gave. Second, the research team only recruited families from within four sites in Karnataka due to time and financial constraints. A wider sample across a more diverse population could have revealed how CCP programs interact with different cultural contexts and norms around neonatal health. Additionally, the majority of the newborn complications group reported non-serious complications (e.g. fever or cold), making it challenging to ascertain impact on health complications or hospital readmissions. Additionally, we chose only to ask complication related questions to families who reported neonatal complications during the 28-day post discharge survey. Finally, given the reliance on self-reported data, there could be social desirability bias or limited recall.

## Conclusion

The CCP, a health education program delivered within hospitals for postnatal caregivers, delivers valuable information for mothers and family caregivers to practice preventive behaviors. The CCP session not only provided details on already known behaviors like breastfeeding or handwashing, but taught new information such as skin-to-skin care. Throughout the interviews, it was clear that there are persisting cultural norms that conflict with health information provided during the CCP, so additional consideration of these norms are needed. Finally, CCP reinforced warning sign recognition.

## Supporting information

S1 Text Postnatal care flipchart in English. (PDF)

S2 Text Interview guide for women who recently delivered in Kannada and English. (PDF)

S3 Text Interview guide for family members in Kannada and English. (PDF)

S4 Text Qualitative codebook with definitions. (PDF)

S1 Checklist Inclusivity questionnaire. (PDF)

## References

[1] UNICEF. India (IND)—Demographics, Health & Infant Mortality [Internet]. UNICEF DATA. 2020 [cited 2021 Nov 19]. Available from: https://data.unicef.org/country/ind/

[2] Jain K, Sankar MJ, Nangia S, Ballambattu VB, Sundaram V, Ramji S, et al. Causes of death in preterm neonates (<33 weeks) born in tertiary care hospitals in India: analysis of three large prospective multicentric cohorts. Journal of Perinatology. 2019 Sep;39(1):13–9. 31485016 10.1038/s41372-019-0471-1 PMC8075971

[3] Berhan D, Gulema H. Level of Knowledge and Associated Factors of Postnatal Mothers’ towards Essential Newborn Care Practices at Governmental Health Centers in Addis Ababa, Ethiopia. Khubchandani J, editor. Advances in Public Health. 2018 Oct 21;2018:8921818.

[4] Fadel SA, Ram U, Morris SK, Begum R, Shet A, Jotkar R, et al. Facility Delivery, Postnatal Care and Neonatal Deaths in India: Nationally-Representative Case-Control Studies. PLoS One [Internet]. 2015 Oct 19 [cited 2021 Feb 3];10(10). Available from: https://www.ncbi.nlm.nih.gov/pmc/articles/PMC4610669/ doi: 10.1371/journal.pone.0140448 26479476 PMC4610669

[5] Causes of neonatal and child mortality in India: nationally representative mortality survey. Lancet. 2010 Nov 27;376(9755):1853–60. 21075444 10.1016/S0140-6736(10)61461-4 PMC3042727

[6] Ram U, Jha P, Ram F, Kumar K, Awasthi S, Shet A, et al. Neonatal, 1–59 month, and under-5 mortality in 597 Indian districts, 2001 to 2012: estimates from national demographic and mortality surveys. Lancet Glob Health. 2013 Oct;1(4):e219–226. doi: 10.1016/S2214-109X(13)70073-1 25104347

[7] Herbert HK, Lee ACC, Chandran A, Rudan I, Baqui AH. Care seeking for neonatal illness in low- and middle-income countries: a systematic review. PLoS Med. 2012;9(3):e1001183. doi: 10.1371/journal.pmed.1001183 22412355 PMC3295826

[8] Phukan D, Ranjan M, Dwivedi LK. Impact of timing of breastfeeding initiation on neonatal mortality in India. Int Breastfeed J. 2018;13:27. doi: 10.1186/s13006-018-0162-0 29988694 PMC6029033

[9] Kinanu L, Odhiambo E, Mwaura J, Habtu M. Cord Care Practices and Omphalitis among Neonates Aged 3–28 Days at Pumwani Maternity Hospital, Kenya. Journal of Biosciences and Medicines. 2016;04(01):27.

[10] Kuti BP, Ogunlesi TA, Oduwole O, Oringanje C, Udoh EE, Meremikwu MM. Hand hygiene for the prevention of infections in neonates. Cochrane Database Syst Rev. 2021 Jan 20;1:CD013326. doi: 10.1002/14651858.CD013326.pub2 33471367 PMC8094276

[11] Shrestha T, Bhattarai SG, Silwal K. Knowledge and practice of postnatal mother in newborn care. JNMA J Nepal Med Assoc. 2013 Jun;52(190):372–7. 24362663

[12] Srivastava S, Gupta A, Bhatnagar A, Dutta S. Effect of very early skin to skin contact on success at breastfeeding and preventing early hypothermia in neonates. Indian J Public Health. 2014 Mar;58(1):22–6. doi: 10.4103/0019-557X.128160 24748353

[13] Singh DR, Harvey CM, Bohara P, Nath D, Singh S, Szabo S, et al. Factors associated with newborn care knowledge and practices in the upper Himalayas. PLoS One. 2019;14(9):e0222582. doi: 10.1371/journal.pone.0222582 31525242 PMC6746396

[14] Nabiwemba EL, Atuyambe L, Criel B, Kolsteren P, Orach CG. Recognition and home care of low birth weight neonates: a qualitative study of knowledge, beliefs and practices of mothers in Iganga-Mayuge Health and Demographic Surveillance Site, Uganda. BMC Public Health. 2014 Jun 2;14(1):546. doi: 10.1186/1471-2458-14-546 24888464 PMC4064282

[15] Withers M, Kharazmi N, Lim E. Traditional beliefs and practices in pregnancy, childbirth and postpartum: A review of the evidence from Asian countries. Midwifery. 2018 Jan 1;56:158–70. doi: 10.1016/j.midw.2017.10.019 29132060

[16] Pati S, Chauhan AS, Panda M, Swain S, Hussain MA. Neonatal care practices in a tribal community of Odisha, India: A cultural perspective. Journal of Tropical Pediatrics. 2014 Jun 1;60(3):238–44. doi: 10.1093/tropej/fmu005 24519674

[17] George M, Johnson A. Postpartum and Newborn Care-A Qualitative study. Indian Journal of Community Health. 2018 Jun 30;30:163–5.

[18] Subramanian L, Murthy S, Bogam P, Yan SD, Marx Delaney M, Goodwin CDG, et al. Just-in-time postnatal education programees to improve newborn care practices: needs and opportunities in low-resource settings. BMJ Glob Health. 2020;5(7). doi: 10.1136/bmjgh-2020-002660 32727842 PMC7394013

[19] Dol J, Campbell-Yeo M, Tomblin Murphy G, Aston M, McMillan D, Gahagan J, et al. Parent-targeted postnatal educational interventions in low and middle-income countries: A scoping review and critical analysis. Int J Nurs Stud. 2019 Jun;94:60–73. doi: 10.1016/j.ijnurstu.2019.03.011 30933873

[20] Parashar M, Singh SV, Kishore J, Kumar A, Bhardwaj M. Effect of community-based behavior change communication on delivery and newborn health care practices in a resettlement colony of Delhi. Indian Journal of Community Medicine. 2013 Jan 1;38(1):42. doi: 10.4103/0970-0218.106627 23559703 PMC3612297

[21] Hazra A, Atmavilas Y, Hay K, Saggurti N, Verma RK, Ahmad J, et al. Effects of health behaviour change intervention through women’s self-help groups on maternal and newborn health practices and related inequalities in rural india: A quasi-experimental study. EClinicalMedicine. 2020 Jan 1;18:100198. doi: 10.1016/j.eclinm.2019.10.011 31993574 PMC6978187

[22] Pillay T. Parent-Carer Education: Reducing the Risks for Neonatal and Infant Mortality. Neonatal Medicine [Internet]. 2019 Jan 21 [cited 2021 Feb 18]; Available from: https://www.intechopen.com/books/neonatal-medicine/parent-carer-education-reducing-the-risks-for-neonatal-and-infant-mortality

[23] Merakou K, Knithaki A, Karageorgos G, Theodoridis D, Barbouni A. Group patient education: effectiveness of a brief intervention in people with type 2 diabetes mellitus in primary health care in Greece: a clinically controlled trial. Health Educ Res. 2015 Apr;30(2):223–32. doi: 10.1093/her/cyv001 25724879

[24] Rickheim PL, Weaver TW, Flader JL, Kendall DM. Assessment of Group Versus Individual Diabetes Education: A randomized study. Diabetes Care. 2002 Feb 1;25(2):269–74. doi: 10.2337/diacare.25.2.269 11815494

[25] Torres H de C, Franco LJ, Stradioto MA, Hortale VA, Schall VT. Evaluation of group and individual strategies in a diabetes education program. Rev Saude Publica. 2009 Apr;43(2):291–8. 19225700

[26] Ministry of Health and Family Welfare. National Family Health Survey-4, State Factsheet Karnataka [Internet]. 2017 [cited 2021 Nov 19]. Available from: http://rchiips.org/NFHS/pdf/NFHS4/KA_FactSheet.pdf

[27] O’Brien BC, Harris IB, Beckman TJ, Reed DA, Cook DA. Standards for Reporting Qualitative Research: A Synthesis of Recommendations. Academic Medicine. 2014 Sep;89(9):1245–51. doi: 10.1097/ACM.0000000000000388 24979285

[28] Kittle B. A Practical Guide to Conducting a Barrier Analysis [Internet]. New York: Helen Keller International; 2013 [cited 2021 Jun 17]. Available from: https://pdf.usaid.gov/pdf_docs/PA00JMZW.pdf

[29] Dedoose [Internet]. Los Angeles, California: SocioCultural Research Consultants, LLC; 2020 [cited 2021 Jun 17]. Available from: https://www.dedoose.com/

[30] Kashyap S, Spielman AF, Ramnarayan N, SD S, Pant R, Kaur B, et al. Impact of family-centred postnatal training on maternal and neonatal health and care practices in district hospitals in two states in India: a pre–post study. BMJ Open Qual. 2022 May 1;11(Suppl 1):e001462. doi: 10.1136/bmjoq-2021-001462 35545272 PMC9092167

[31] Lunkenheimer HG, Burger O, Akhauri S, Chaudhuri I, Dibbell L, Hashmi FA, et al. Tradition, taste and taboo: the gastroecology of maternal perinatal diet. BMJ Nutrition, Prevention & Health. 2021 Jul 1;bmjnph. doi: 10.1136/bmjnph-2021-000252 35028510 PMC8718855

[32] Aubel J. Grandmothers—a neglected family resource for saving newborn lives. BMJ Global Health. 2021 Feb 1;6(2):e003808. doi: 10.1136/bmjgh-2020-003808 33589417 PMC7887373

[33] Aubel J. The role and influence of grandmothers on child nutrition: culturally designated advisors and caregivers. Matern Child Nutr. 2011 Sep 28;8(1):19–35. doi: 10.1111/j.1740-8709.2011.00333.x 21951995 PMC6860857

[34] Langer A, Meleis A, Knaul FM, Atun R, Aran M, Arreola-Ornelas H, et al. Women and Health: the key for sustainable development. The Lancet. 2015 Sep 19;386(9999):1165–210. doi: 10.1016/S0140-6736(15)60497-4 26051370

