# Supplementary material for: Qualitative assessment of family caregiver-centered neonatal education program in Karnataka, India
Source: PLOS Glob Public Health. 2023 Feb 14;3(2):e0000524. doi: 10.1371/journal.pgph.0000524 (PMC10022017; doi:10.1371/journal.pgph.0000524)
Supplement: S1 Text — (PDF) [file pgph.0000524.s001.pdf]

Postnatal Care

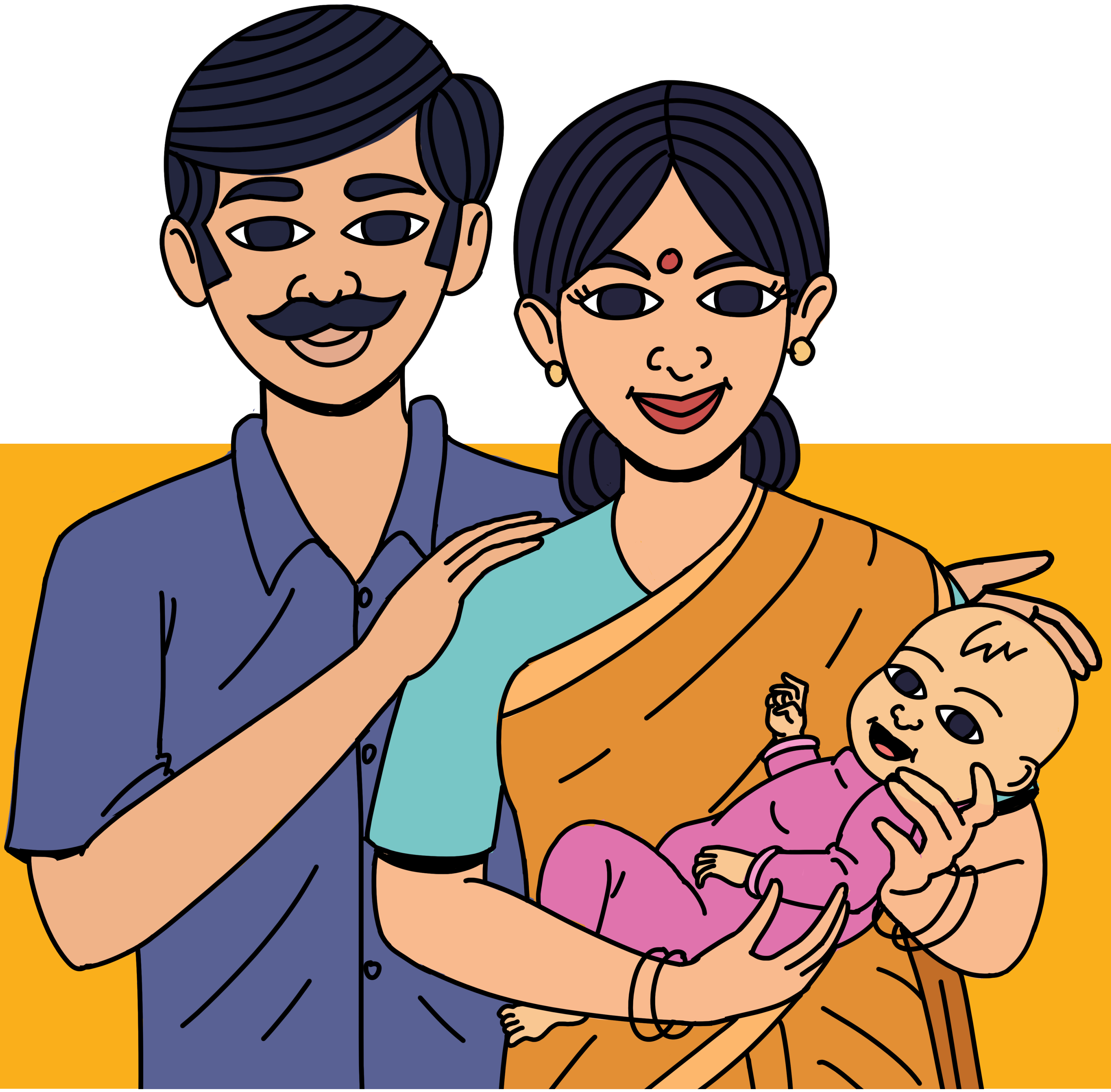

CARE COMPANION PROGRAM

20th April 2022

NOORA HEALTH

YOSAID INNOVATION FOUNDATION

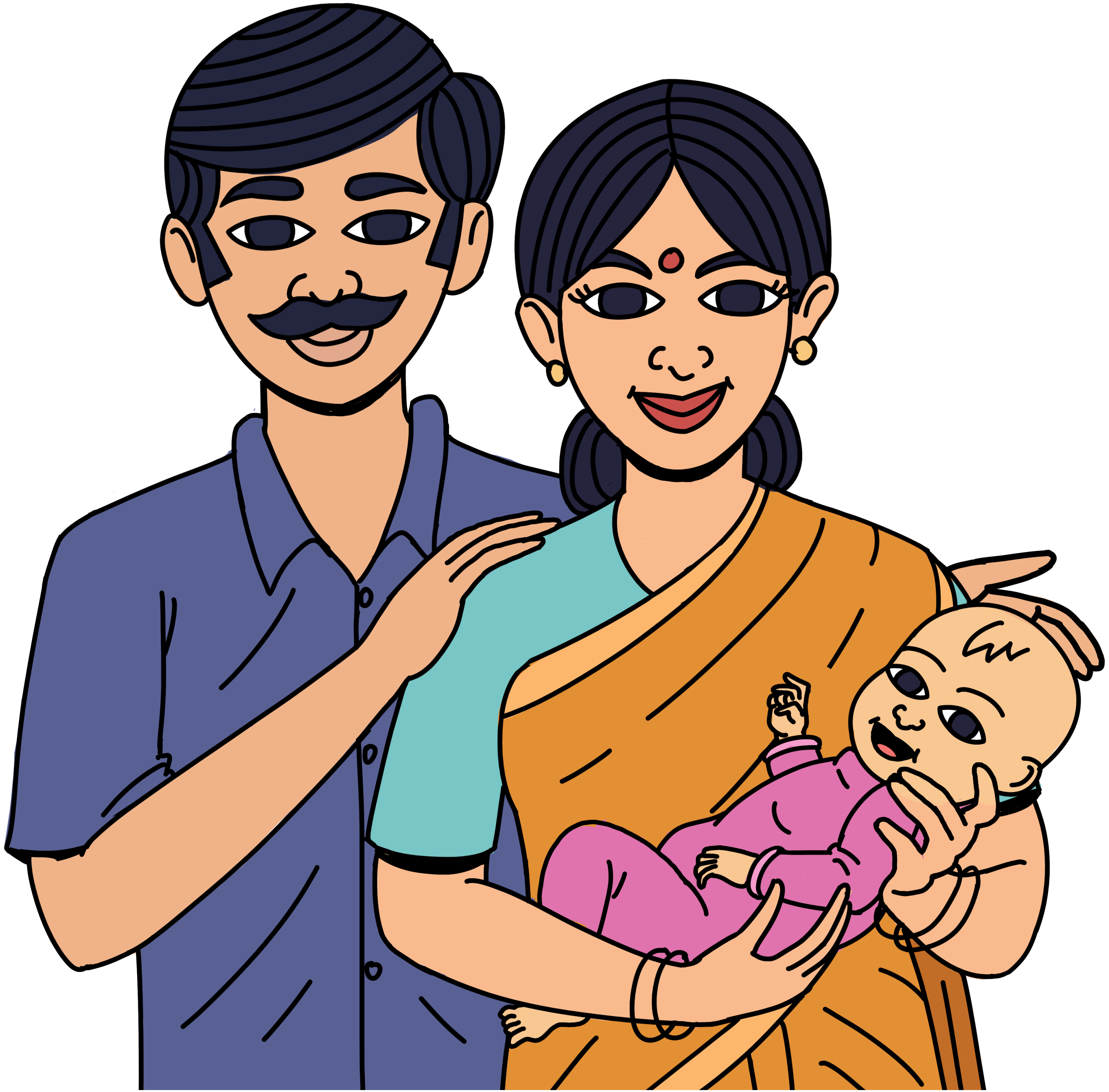

INDEX

Introduction 4

Breastfeeding 6

Burping 8

List of Danger Signs in Baby 10

Breathing Difficulty in Baby 12

Umbilical Cord Care and Infection 14

Jaundice in Newborns 16

Diarrhea and Water Loss in Baby 18

Handwashing 20

KMC/Skin-to-Skin Care 22

Diet and Healthy Behaviors for the Mother 26

Follow Up for - Mother and Baby 28

WhatsApp Onboarding 30

Discussion Section 32

1. Maternal Complications and Less Milk
2. Correct Way to Breastfeed and Breast Problems
3. Hand Expression of Breast milk

Introduction

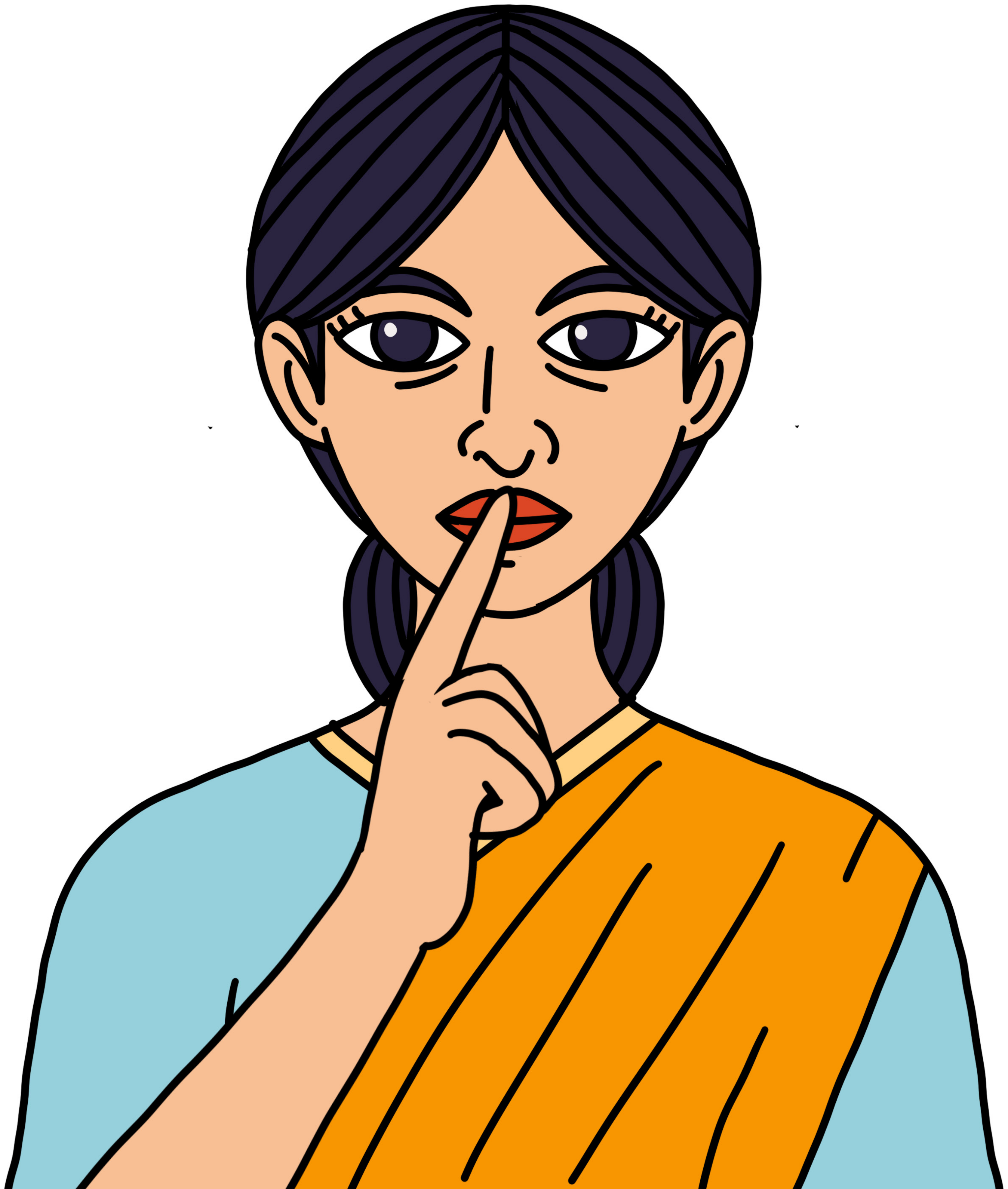

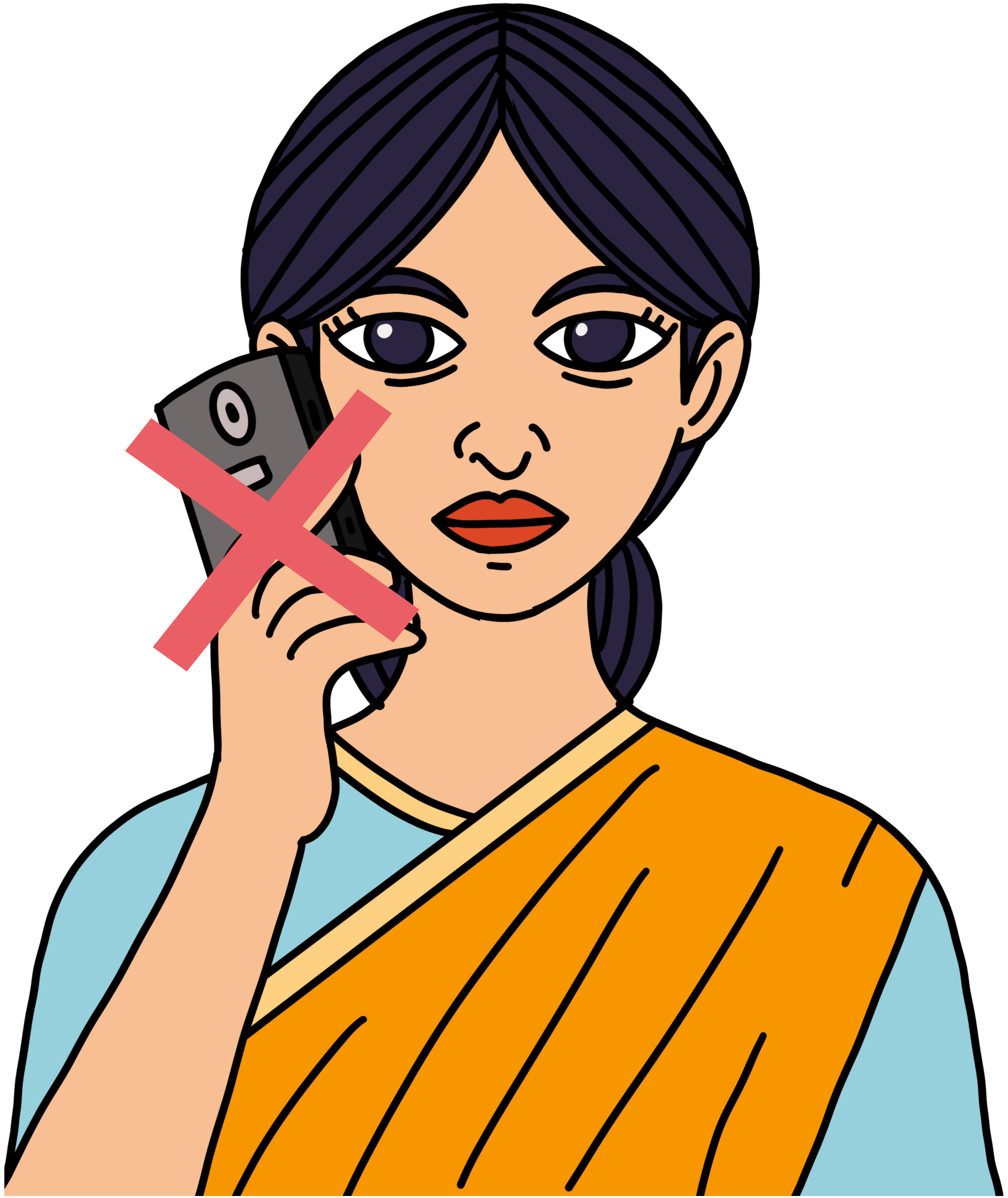

Introduction

Namaste,

My name is _______________.

I am a **Nursing officer** in this hospital, working for the last ____ years/ months.

Firstly, let me congratulate you on the arrival of the baby into your family!

This **Care Companion Program** has been arranged by our hospital on behalf of the government.

Today, I will teach you important things on how to take care of your baby and the mother while in this **hospital and at home.**

Request to participants

Before the start of the session, we request you to

- Please keep your mobile phones in silent mode or switch it off
- Please be quiet and listen while I am speaking
- If you have other work, you can go out and do it, **without disturbing others**
- **Please sit till the end of the session to understand all the topics. You can ask questions any time**

1. Breastfeeding

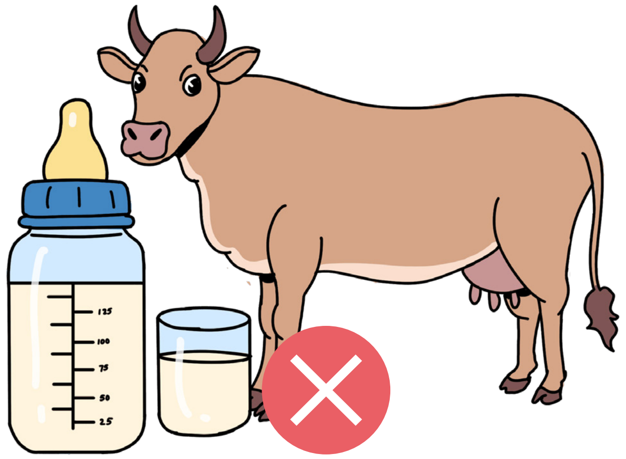

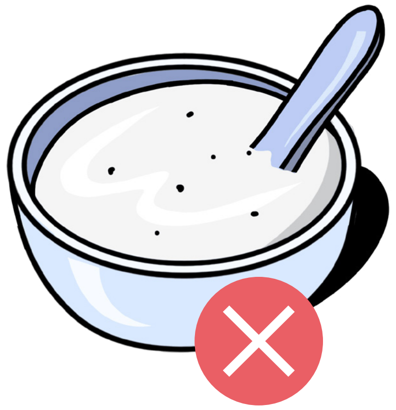

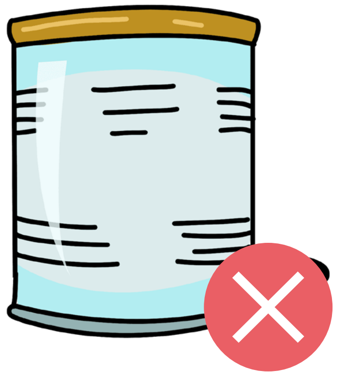

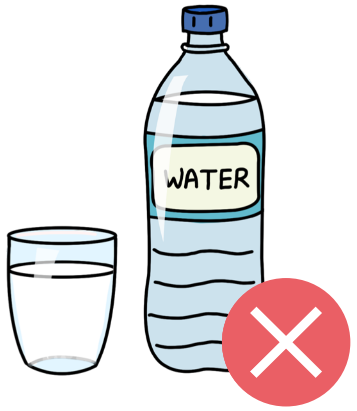

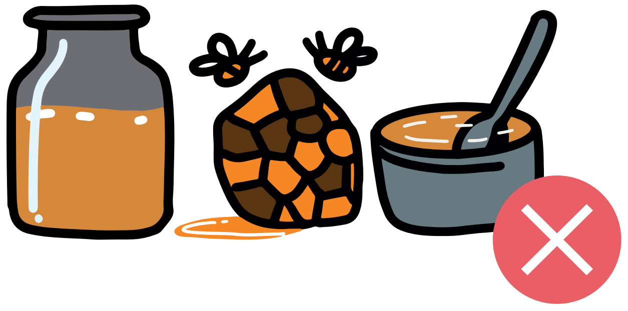

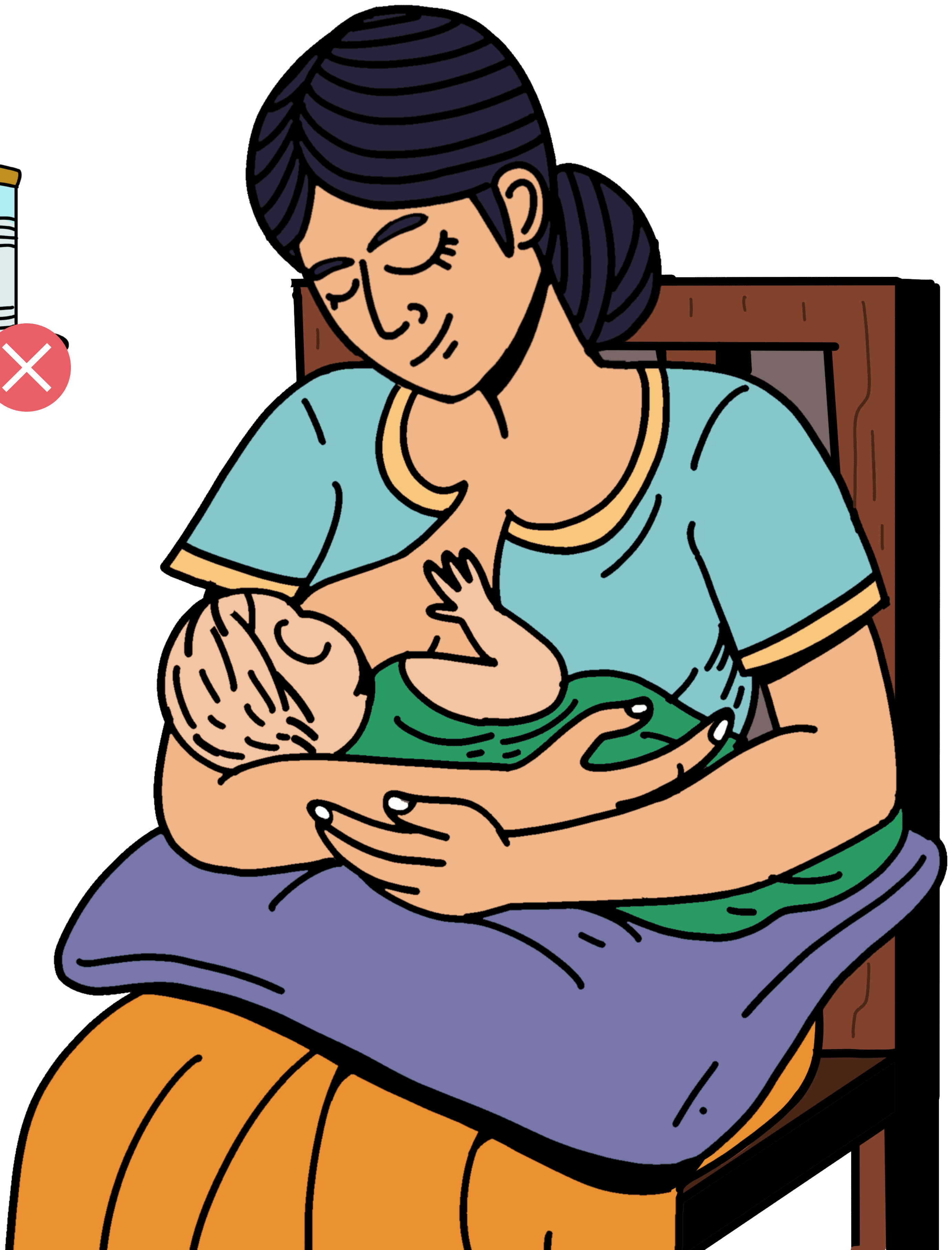

1. Breastfeeding

Feed only breast milk to your baby from birth till 6 months.

Why is breastfeeding important?

For baby it

- Provides all nutrients for growth
- Is easy to digest
- Provides immunity

When to breastfeed?

Start breastfeeding immediately after delivery, if the baby and mother are healthy. The baby must be **given mother's first thick yellow milk.**

Breastfeed the baby **on demand or at least every 2- 3 hours,** even during the night time. Awaken the baby if it sleeps in between.

Don't think the milk is not sufficient. Feed confidently! Most mothers produce as much as the baby needs.

The more the baby sucks, the more milk is produced.

Breast milk is the safest, cheapest and the best food for the baby.

Question

Can you breastfeed the baby if the mother or the baby is sick?

Yes. It is important to continue to breastfeed the baby.

Harmful practices
(Explain using the pictures)

- **Do not feed cow's milk, honey, gripe water** or sugar water
- Giving anything else may lead to infections
- Powder milk given incorrectly causes malnutrition

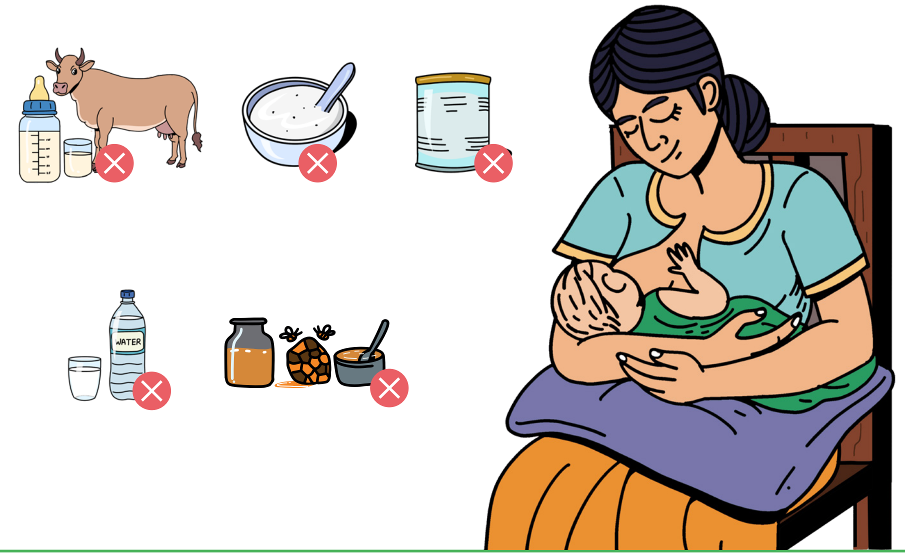

2. Burping

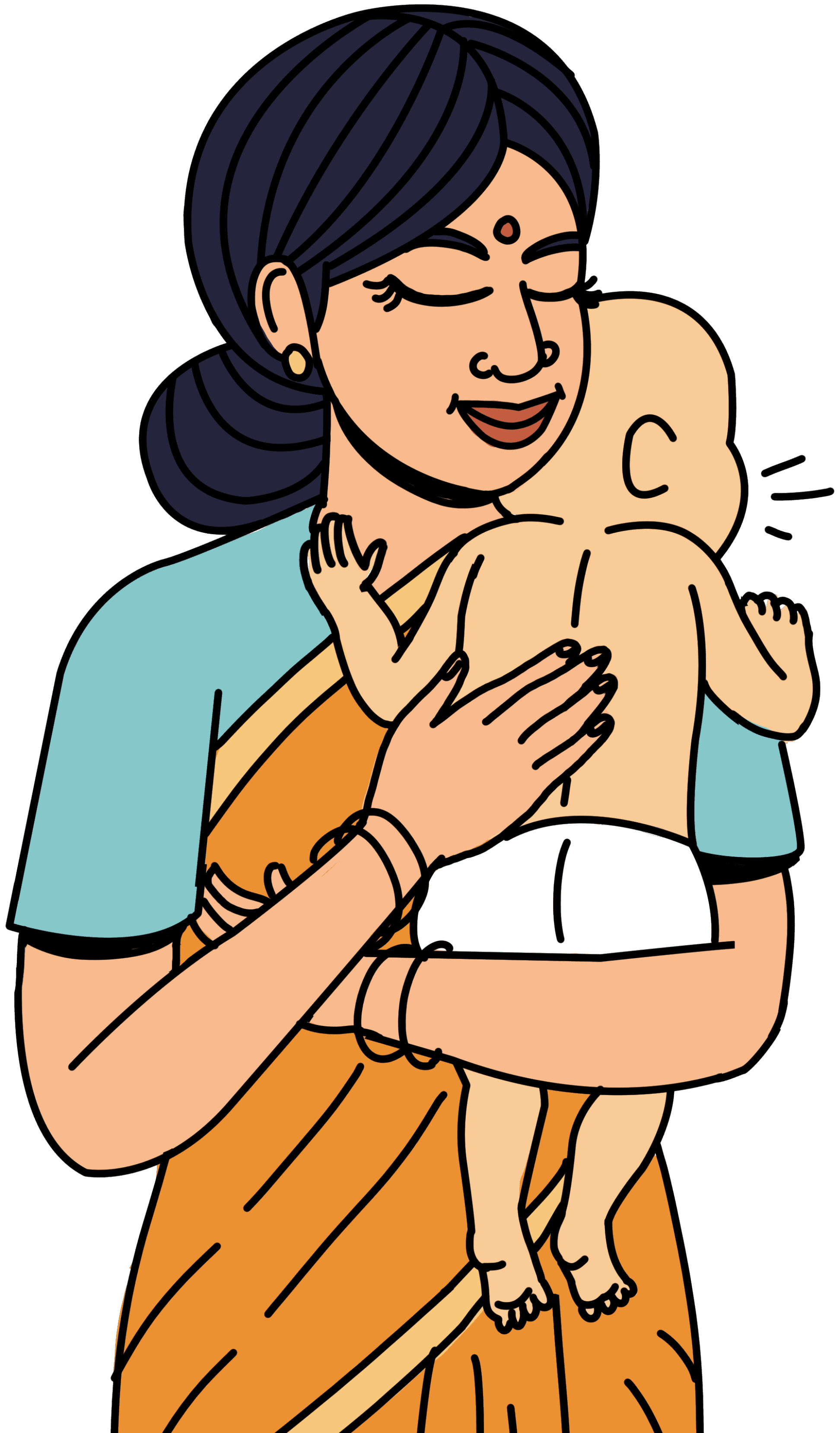

2. Burping

NOORA HEALTH

Why burping is important?

Air gets accumulated in the baby's stomach while feeding.

It is essential to remove this air.

Burping

- Reduces the risk of **vomiting and choking** and associated danger to life
- Baby continues to feed well
- Makes the baby comfortable

Tips for burping

- **Do not put down the baby immediately after breastfeeding**
- Burp the baby after each feed and also in between changing the breasts while feeding.
- It prevents the milk from going inside the breathing pipe and prevents choking.

Burp the baby every time after feeding.

Demonstration

(Demonstrate using a doll)

Steps of burping

1. Hold the baby over your shoulder.
2. Turn the head of the baby outwards so that the baby can breathe properly.
3. Pat over the back gently with your cupped palm or rub the back.
4. Keep in this position for a few minutes till the baby is able to burp.

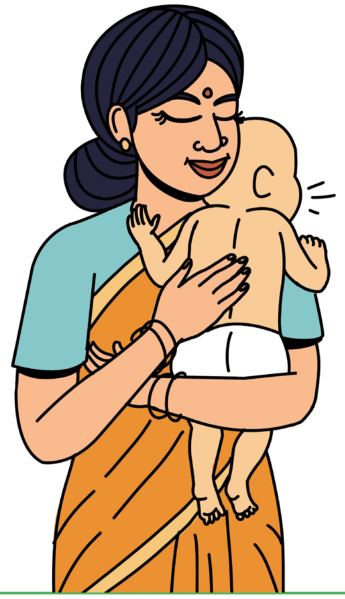

3. List of Danger Signs in Baby

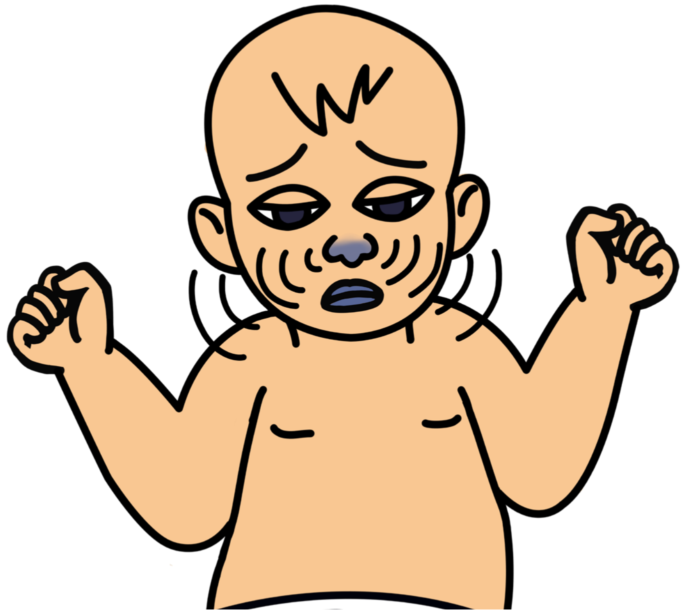

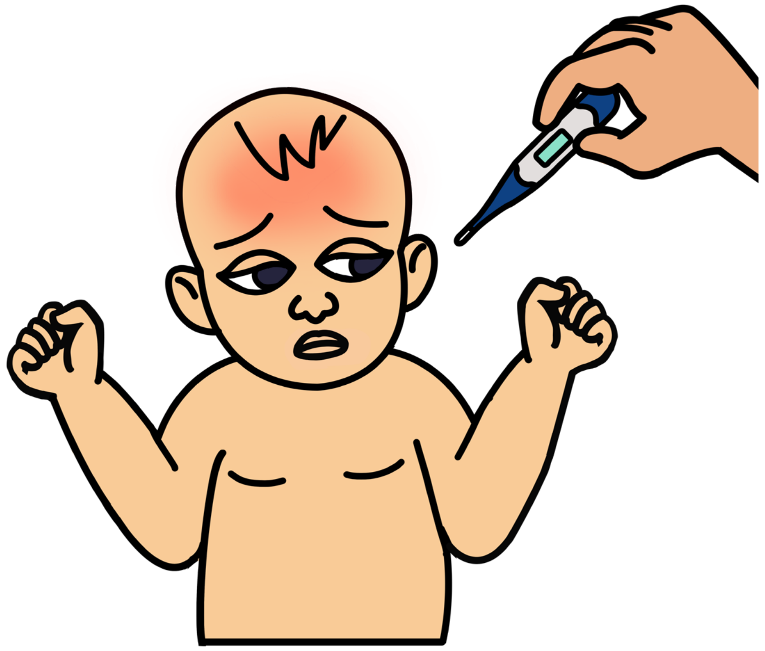

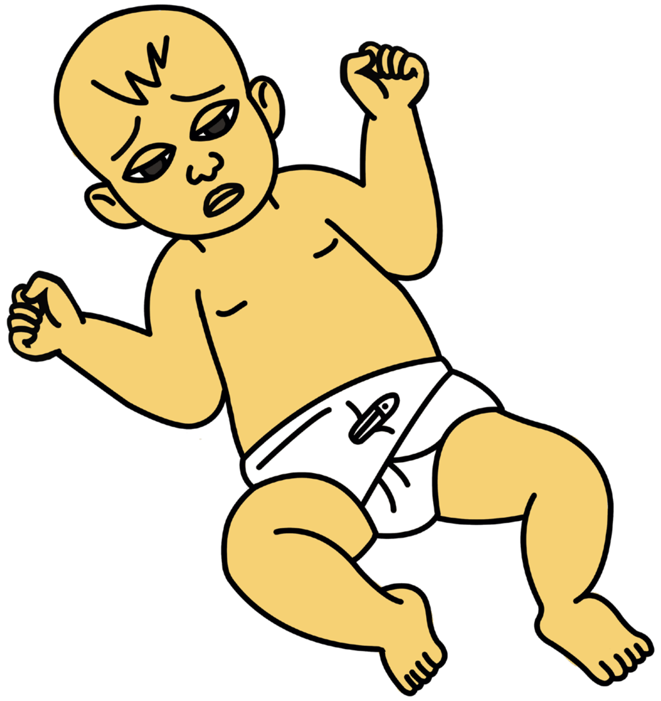

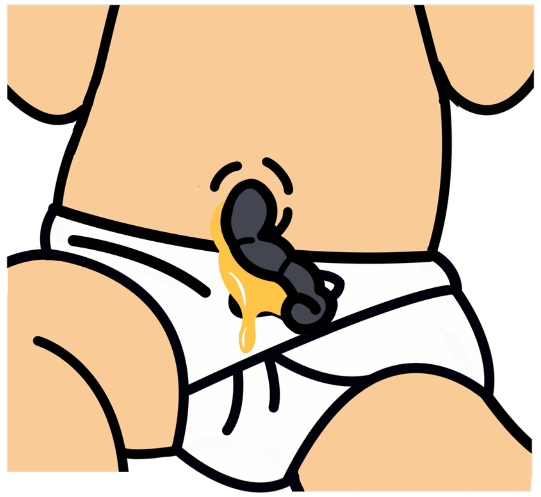

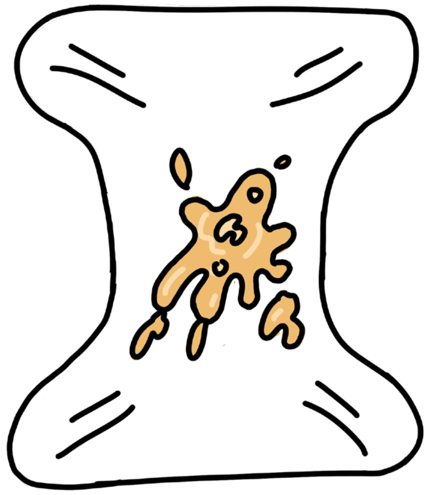

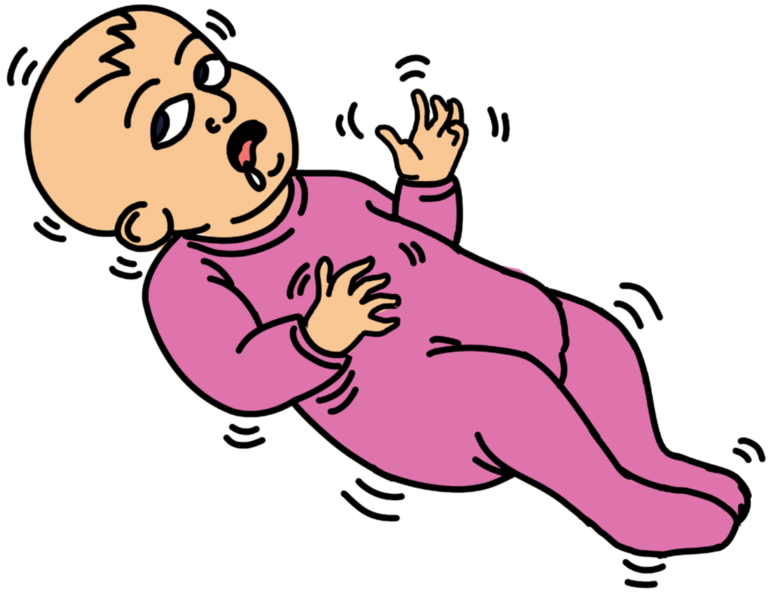

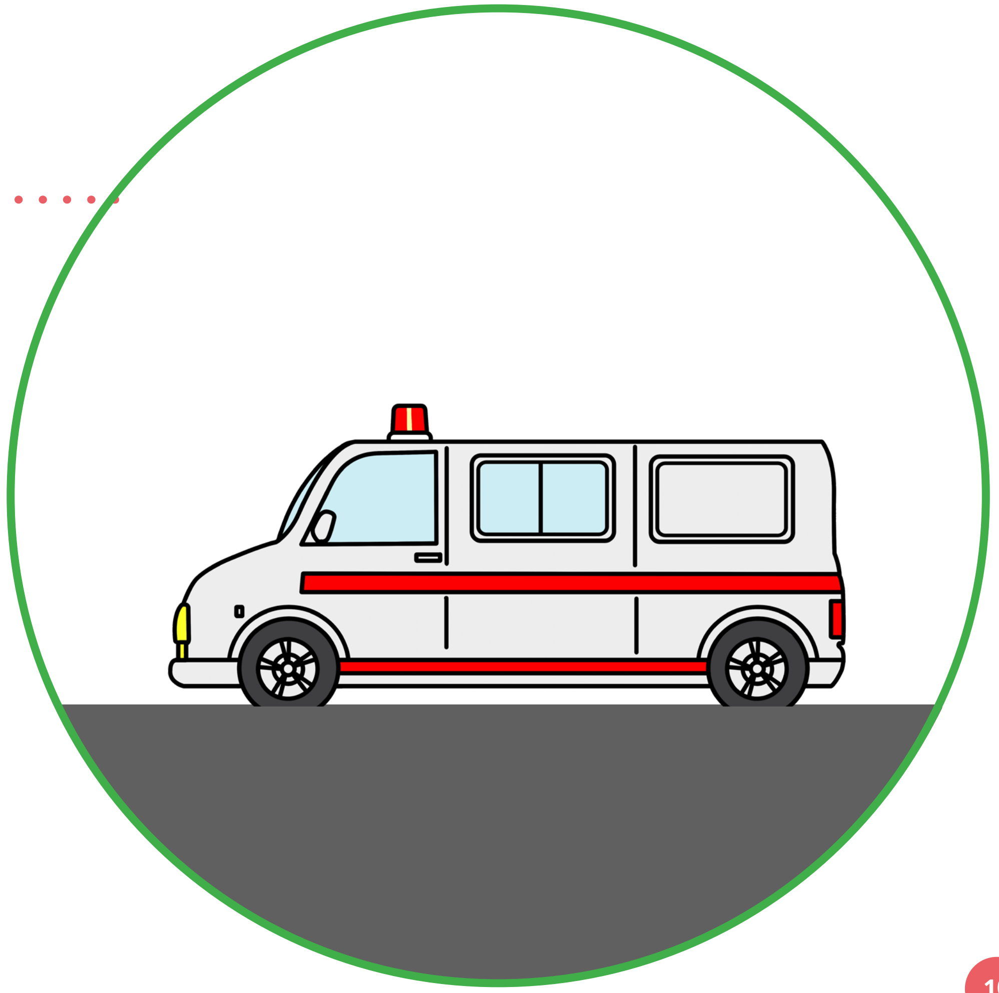

3. List of Danger Signs in Baby

Danger signs are like red traffic lights meaning
'stop, there is danger ahead'
identifying them early can save lives.

How to check

(Demonstrate using a doll)

Starting from top, look for these danger signs in your baby: 3+3+3

Face
1. Breathing difficulty or rapid breathing
2. Inability to suck at breast or not feeding properly
3. Fever- High fever can cause fits

Body (Chest, hands and abdomen)
1. Jaundice- yellow body, yellow palms and soles
2. Coldness of body
3. Pus or redness around cord

Whole
1. Passing frequent loose stools or blood in stools
2. Lethargic or weak
3. Tightness of the body/fits or any other problems in your baby

Early identification and right treatment can save a baby!

Activity

(Ask participants to re-list all the danger signs using the baby doll)

Starting from top, 3+3+3

What to do?

- Identify and rush the baby to the hospital
- Do not ignore such signs
- Do not give any medicine on your own
- The doctor will examine the baby and give medicines accordingly

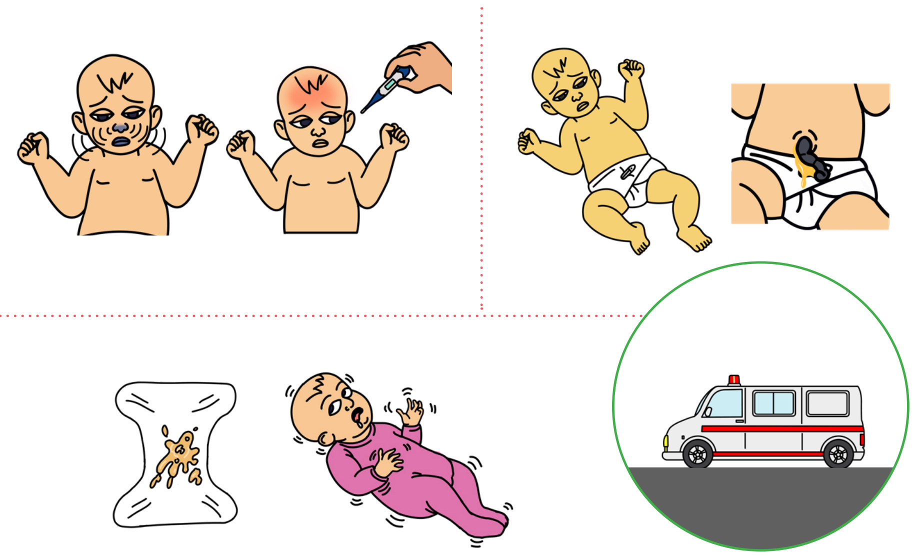

4. Breathing Difficulty in Baby

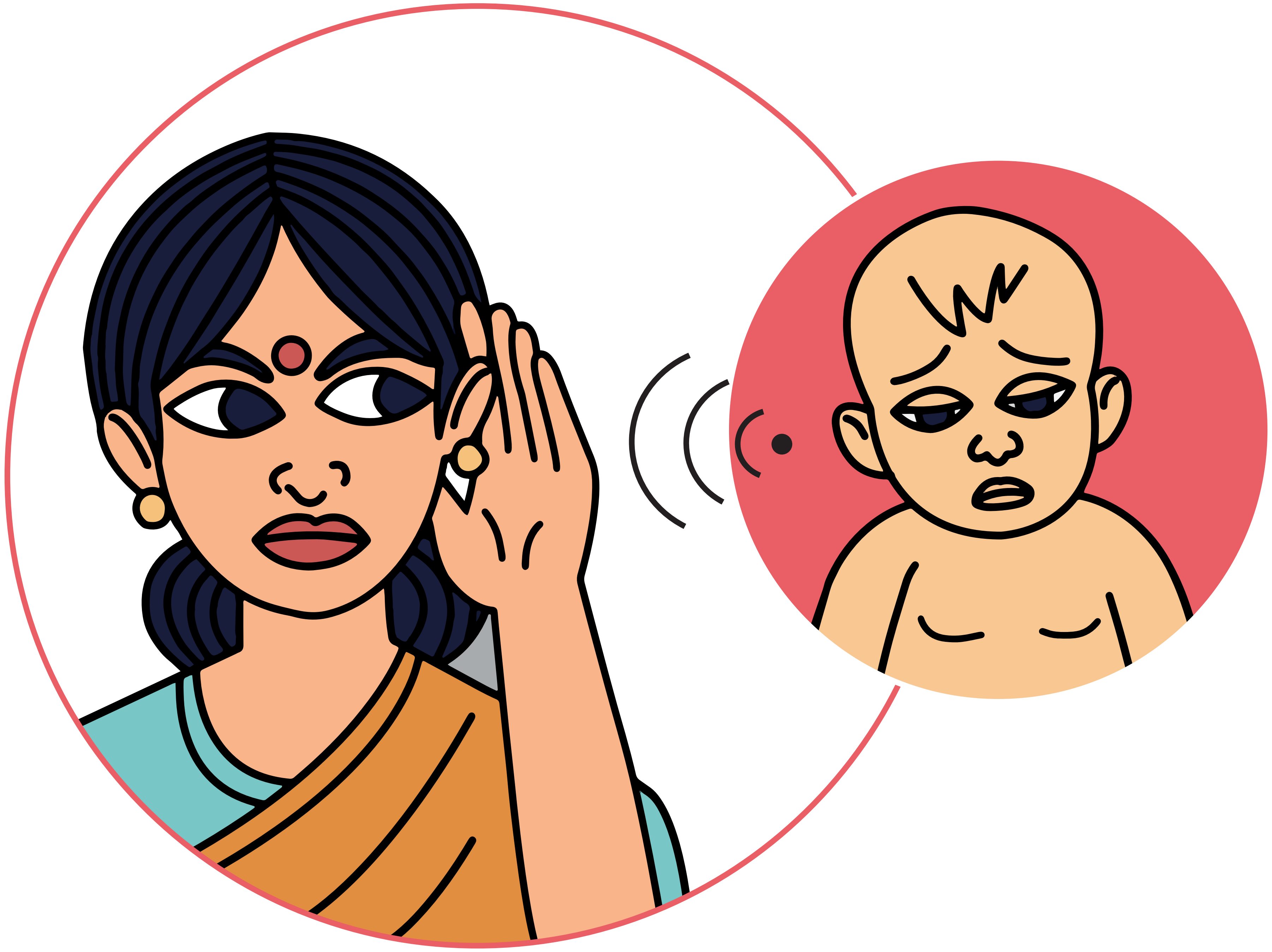

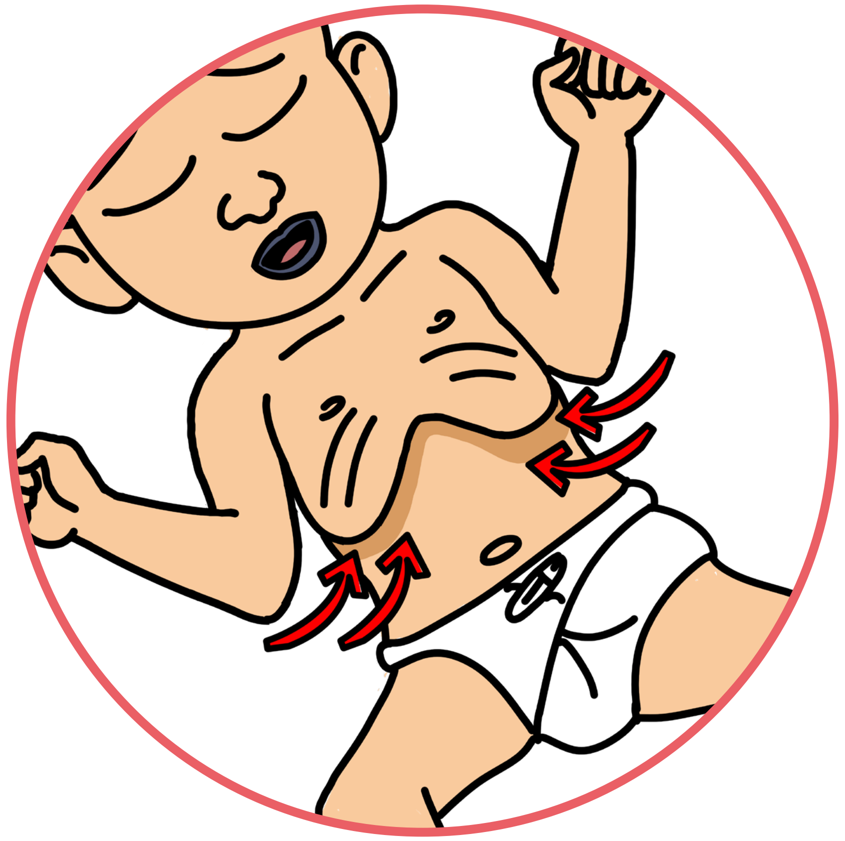

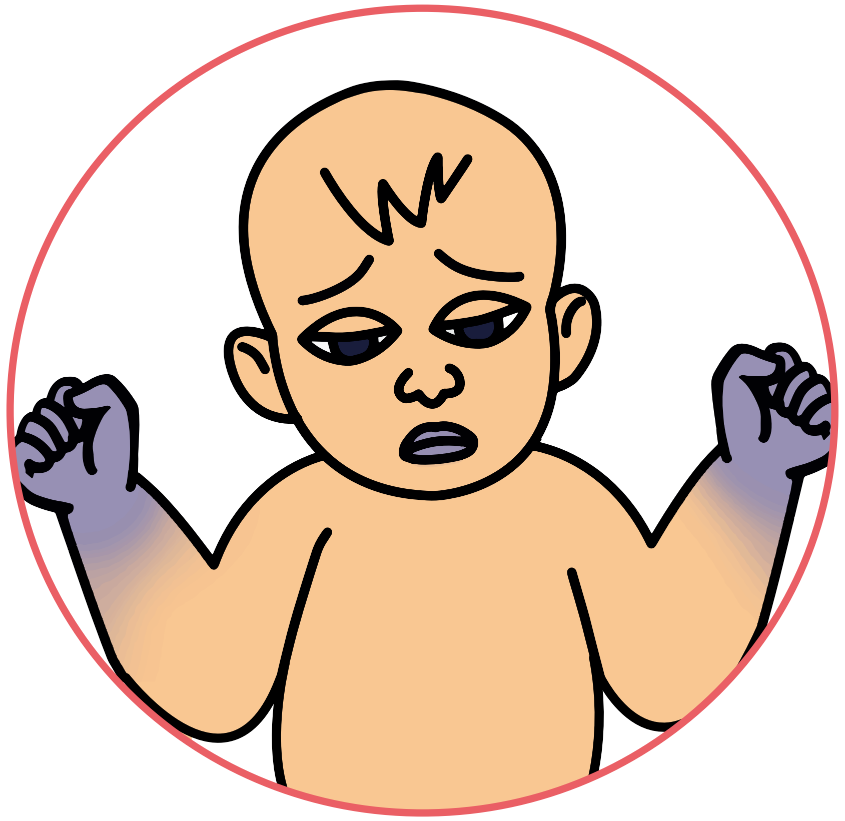

4. Breathing Difficulty in Baby

Breathing problems in a newborn may be because of pneumonia, fever or heart problems.

This is a common cause of death in babies.

Learn to recognise it early.

If you notice any of these signs, **rush the baby to the hospital immediately.**

Recognise early and save the baby's life!

Demonstration

(Show in a doll and say)

How can you check?

Look

- Is the baby breathing with difficulty?

Listen

- Do you hear a grunting noise while breathing out?

Look at the face

- Are lips blue?
- Are baby's nostrils opening wide while breathing in?

Look at the naked chest

- Is the baby breathing rapidly?
- Is the lower chest wall, just below ribs going in while breathing in?

Look at palms and soles

- Is it blue?

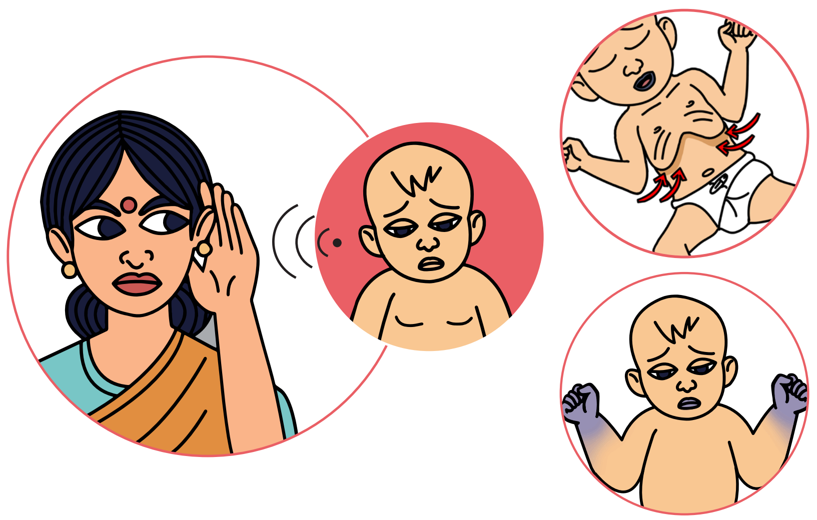

5. Umbilical Cord Care and Infection

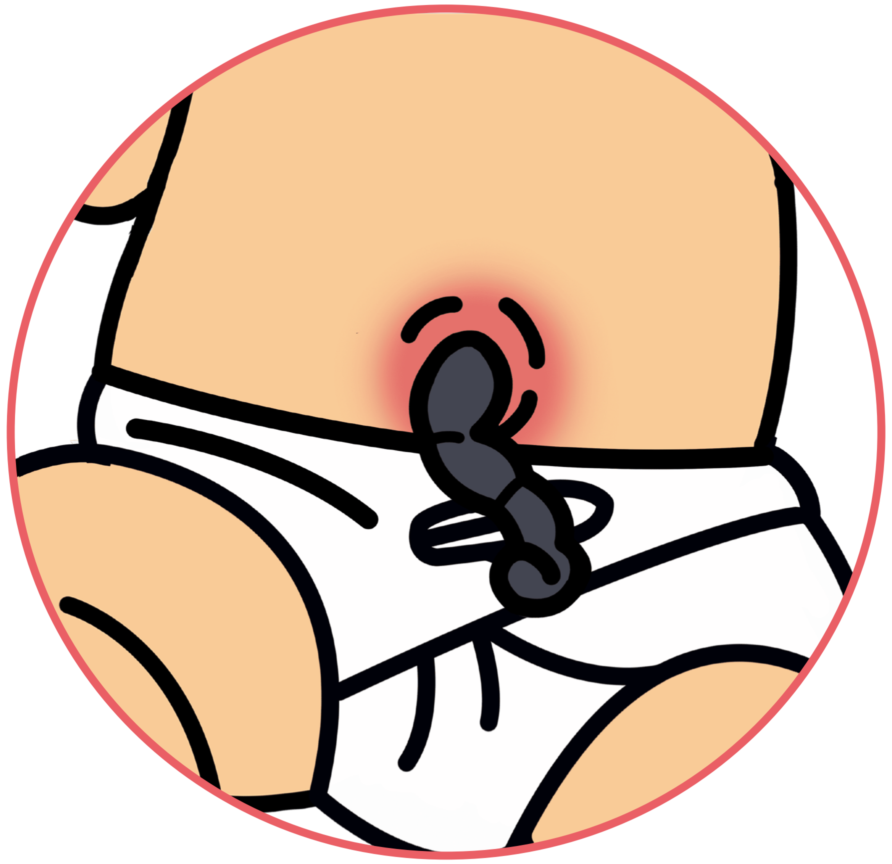

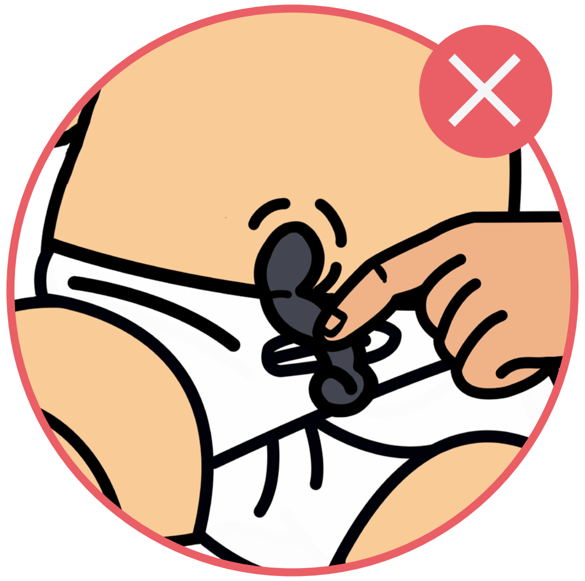

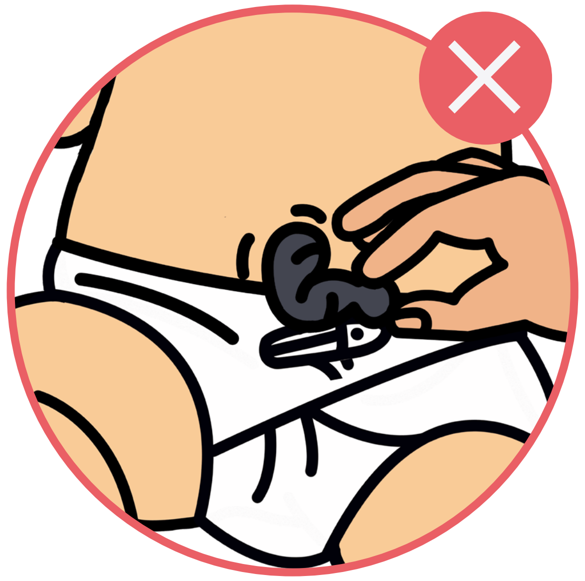

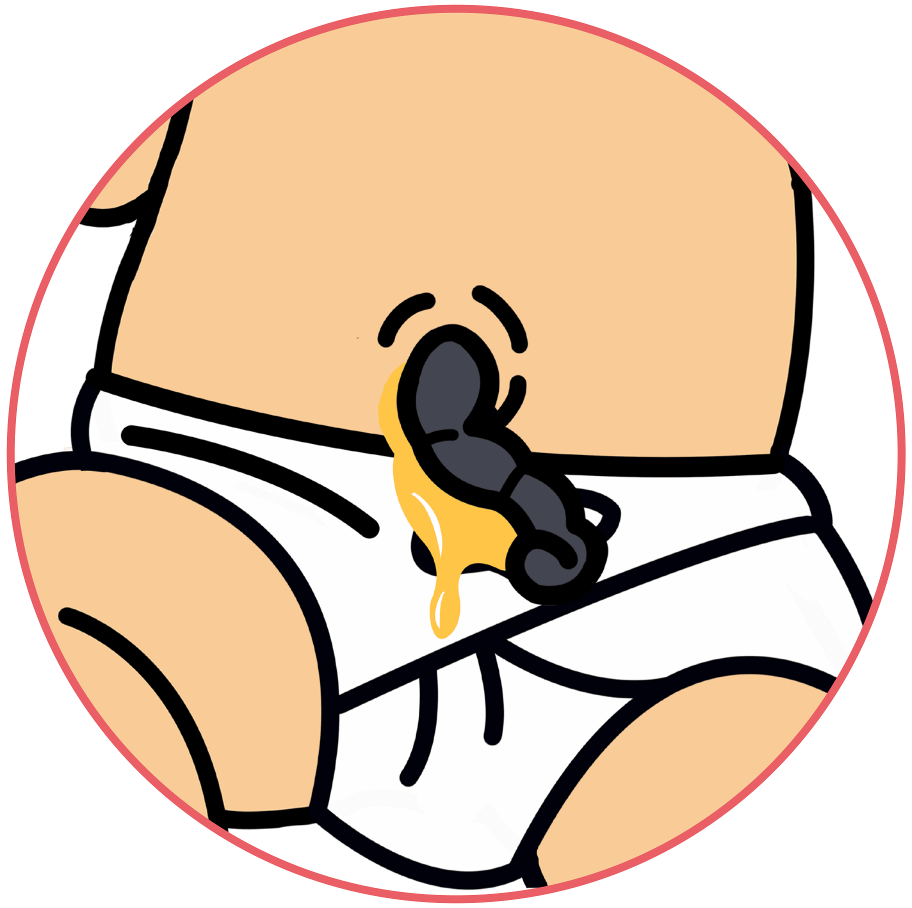

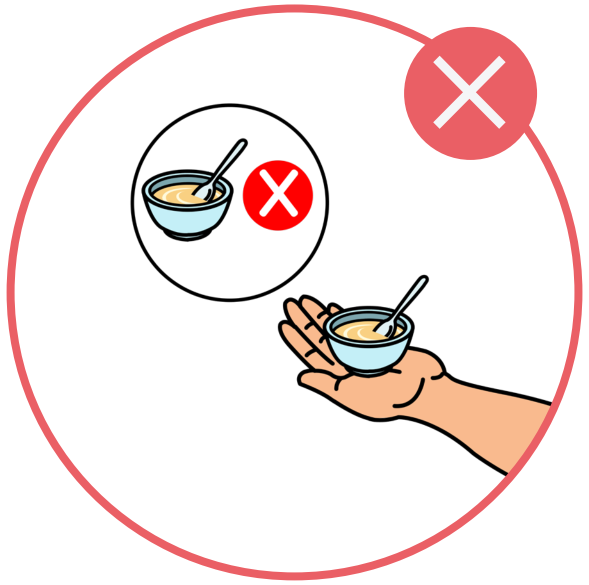

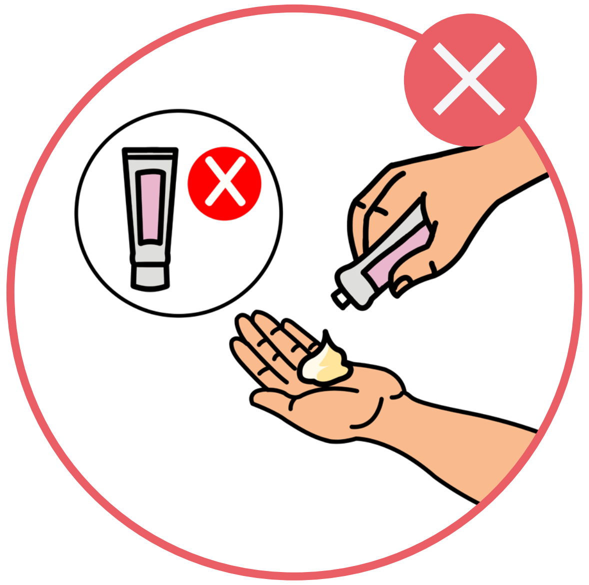

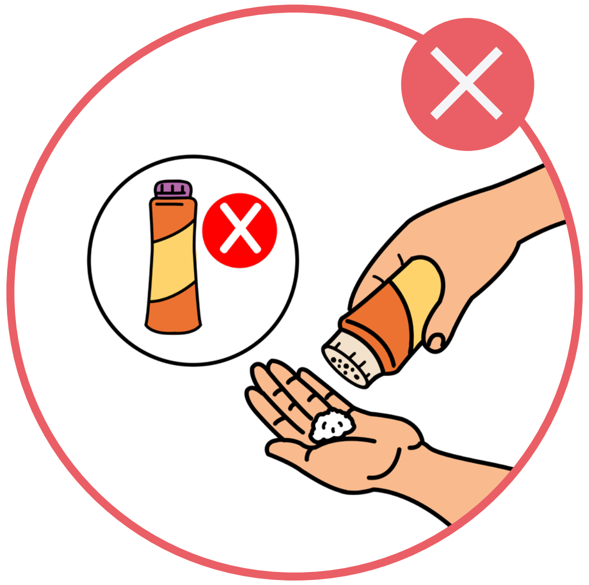

5. Umbilical Cord Care and Infection

Umbilical cord usually dries and falls off on its own in 1 to 2 weeks. Be patient even if delayed

How should you care for the cord?

- Keep the cord and area around it **dry and clean** at all times
- After bath, dry the cord area with a clean cotton cloth

- **Do not apply anything (powder/ oil/ ghee/ ash/ turmeric/ cow dung)**
- Don't touch it unnecessarily.
- Dont pull it out

Applying anything on the cord can make it infected and make the baby very sick!

Be patient! Don't touch! Let the cord fall off by itself!

Question

Do you know how to recognise cord infection?

Look for

- Redness around cord
- Swelling
- Pus with foul smell
- Baby cries if you touch the cord

What can you do?

- Continue to breastfeed
- Go to a hospital immediately

- Do not apply any medicine or herbs on your own

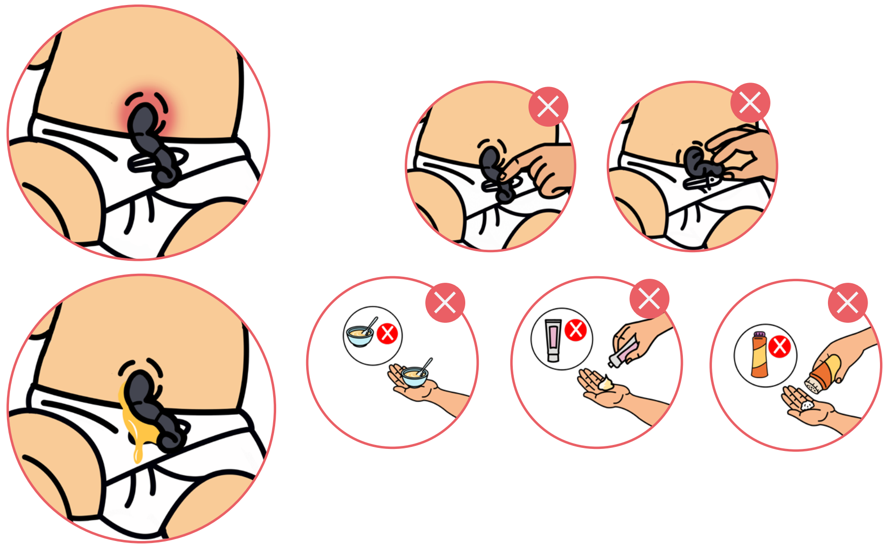

6. Jaundice in Newborns

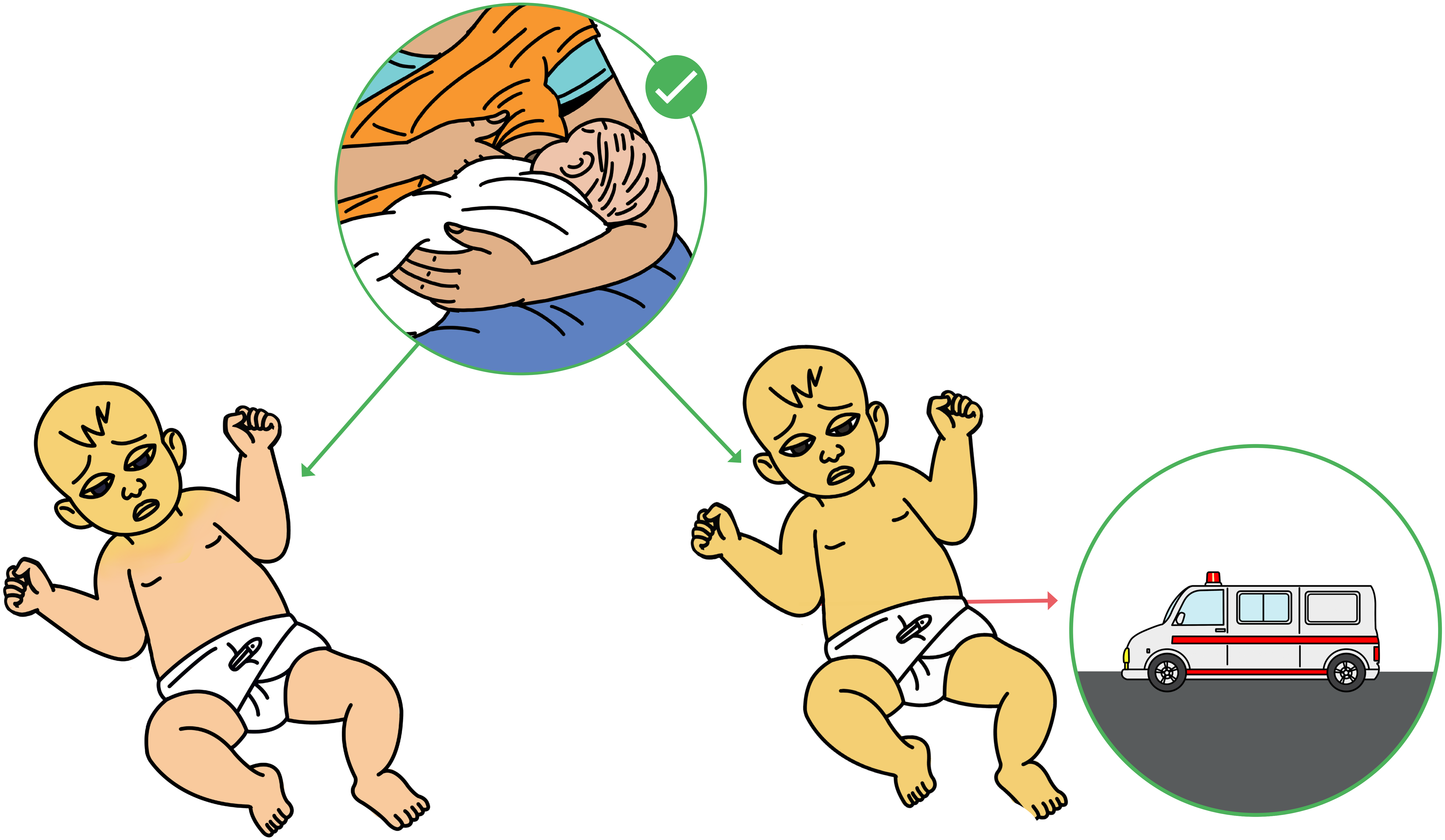

6. Jaundice in Newborns

NOORA HEALTH

What is jaundice?

- Jaundice or yellowness of the body is a common condition in newborns
- Mostly it becomes better by itself
- In some cases, it becomes severe affecting the brain, spine and hearing

Jaundice mostly starts from the face and spreads downwards.

How to check for jaundice?

(Demonstrate using a doll)

For mild jaundice

- Check baby's eyes, face and lips
- Press the baby's nose tip for a few seconds with your finger
- The skin looks yellow after removing the finger

What to do?

- **Breastfeed frequently**
- **Take the baby to the hospital. Doctor may do a blood test**

Identify jaundice at the earliest and take the baby for treatment!

Question

For severe jaundice

- **Palms and soles become yellow or the skin deep yellow**

What to do?

- Breastfeed frequently
- Take the baby to the hospital without delay
- **Baby may need admission and treatment**

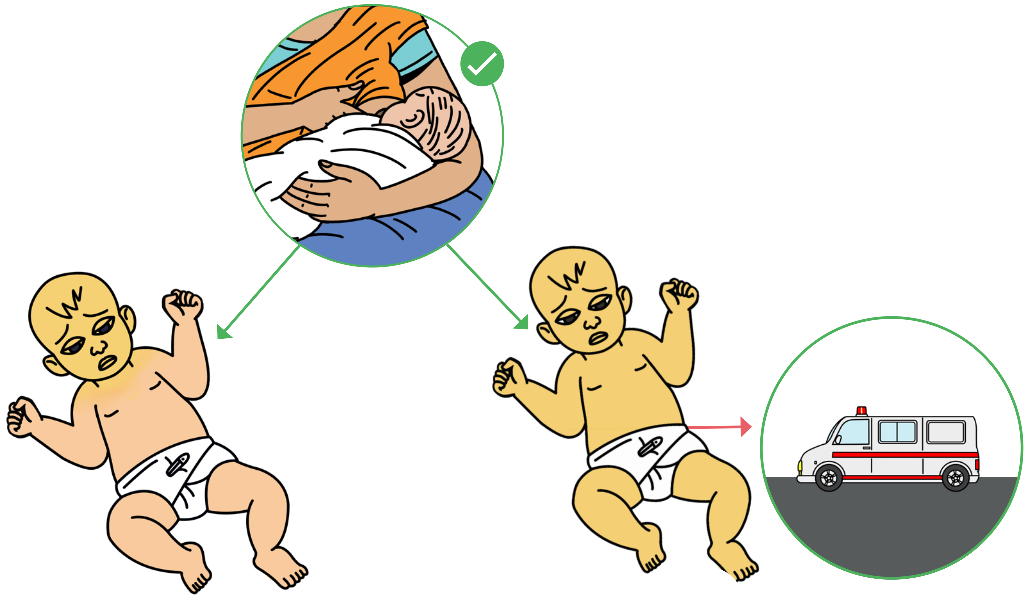

7. Diarrhea and Water Loss in Baby

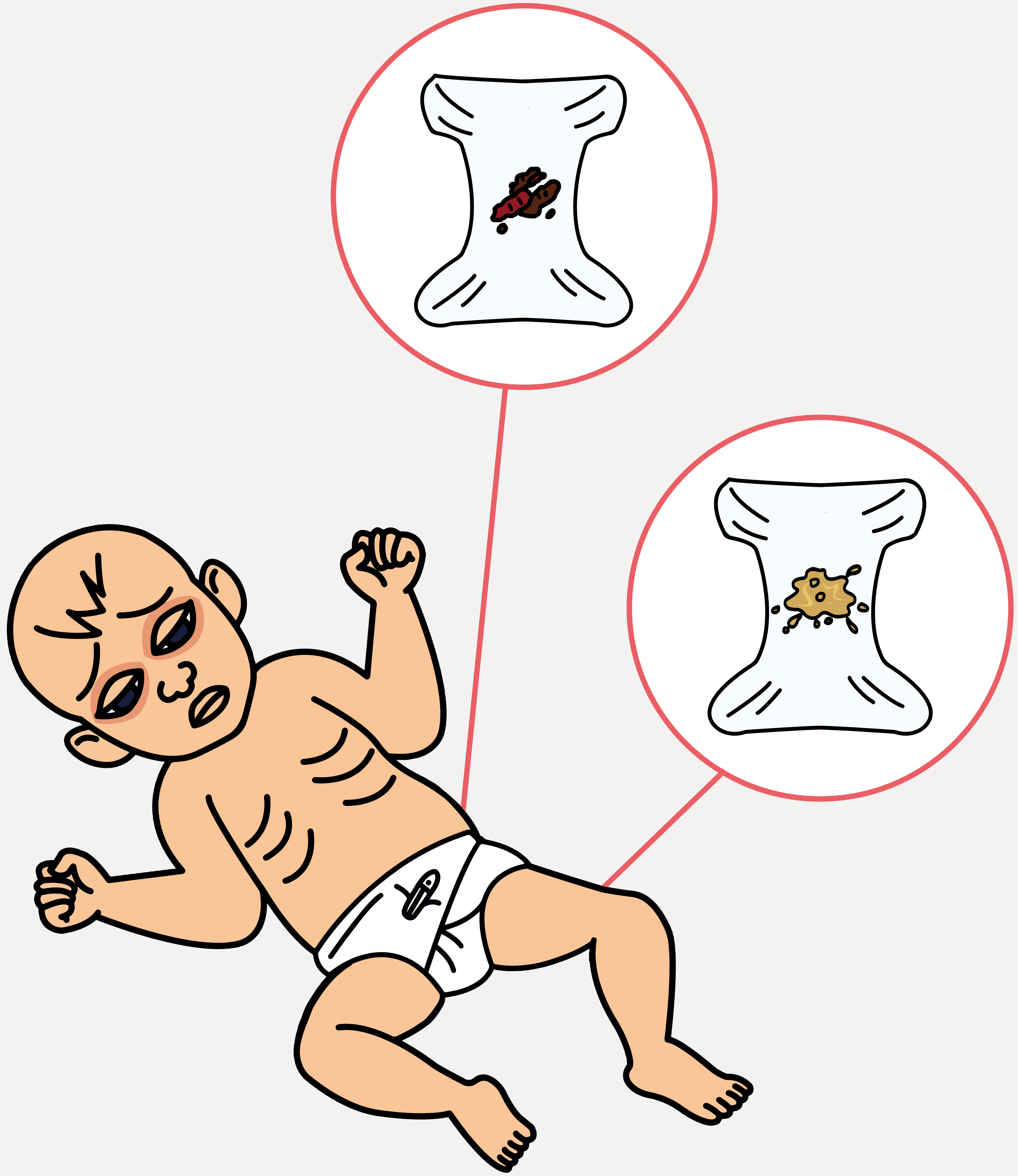

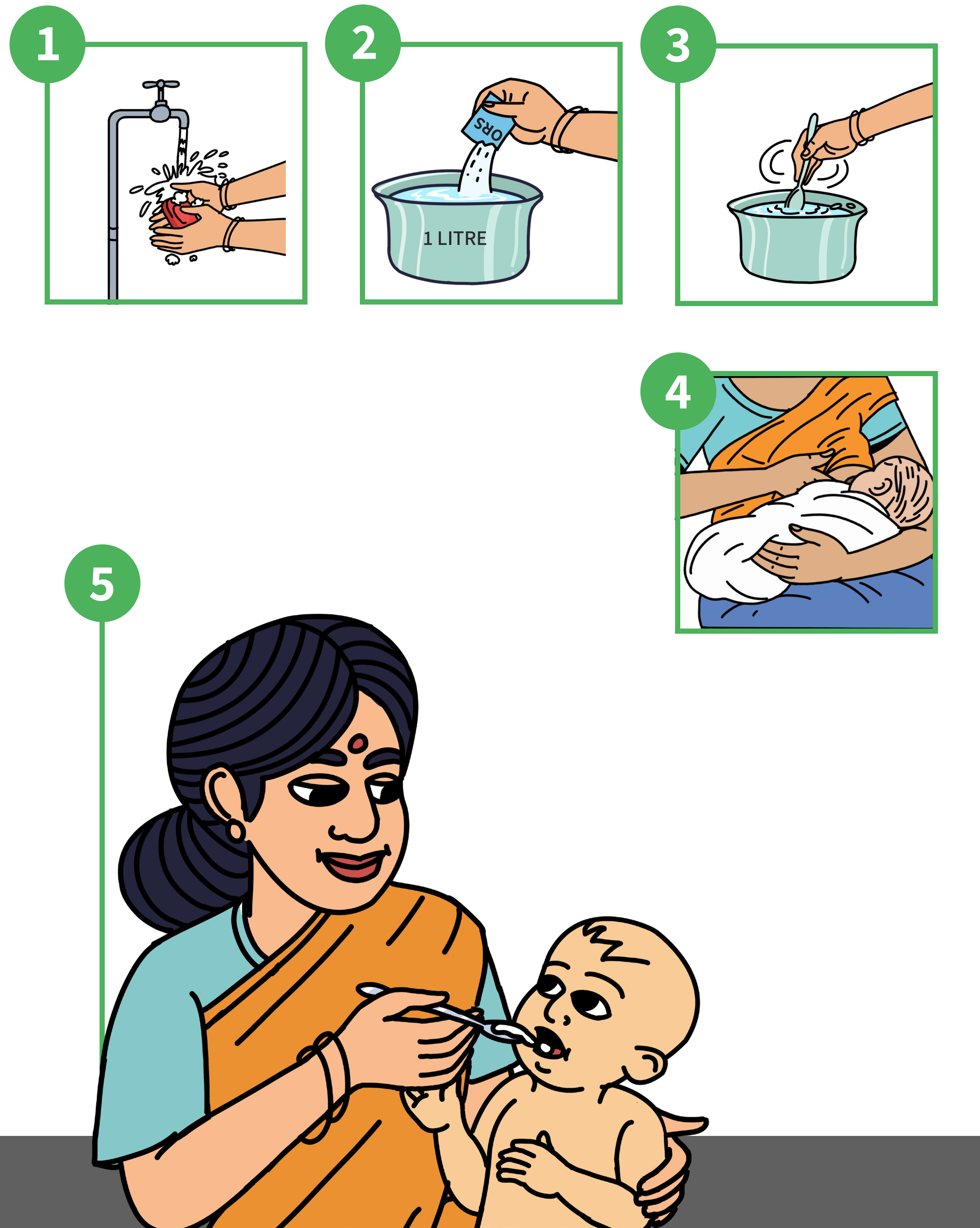

7. Diarrhea and Water Loss in Baby

Diarrhea is when there **is a change in consistency** of the stools. The baby passes **loose or watery stools (more water than fecal matter), 3 times or more** in a day.

Breastfeeding babies passing usual consistency stools or pasty stools frequently is not diarrhea.

How to manage at home?
- Increase breastfeeding
- Give ORS after breastfeeding

When to go to a hospital?
- Blood in stools
- Develops fever
- Diarrhea continues beyond 2 weeks

Rush to a hospital if the baby has water loss. Give ORS on the way.

Identifying water loss in the baby
- Sunken eyes
- Restless or irritable
- Passing less urine (less than 6 wet nappies in a day)
- Not sucking at breast or drinking poorly
- Lethargic or unconscious

Breastfeed frequently. Prevent water loss in your baby!

Question

How to prepare ORS?
- Wash hands
- Empty ORS packet into a clean container
- Add 1 liter boiled and cooled water
- Mix, keep it covered and use for a day

How much ORS to give?
After every loose stool:
- <2 months baby, give 5 spoons of ORS.
- 2-24 months baby, give quarter to half glass of ORS + Zinc tablets for 14 days, as suggested by ASHA.

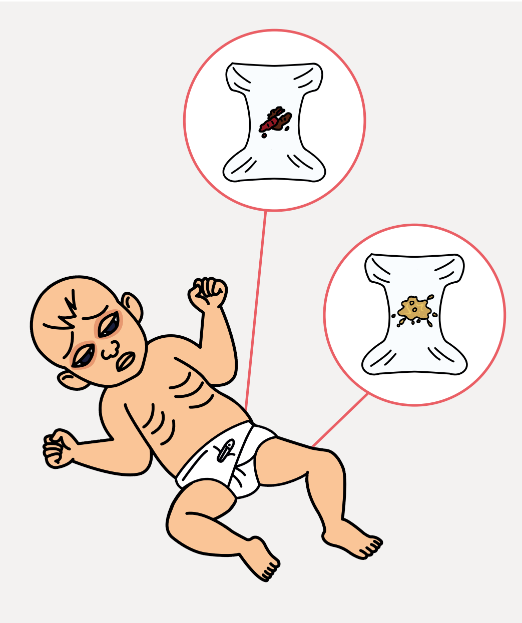

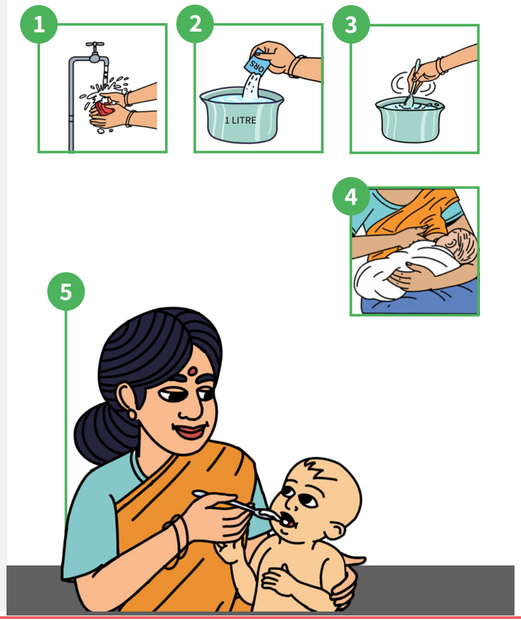

8. Handwashing

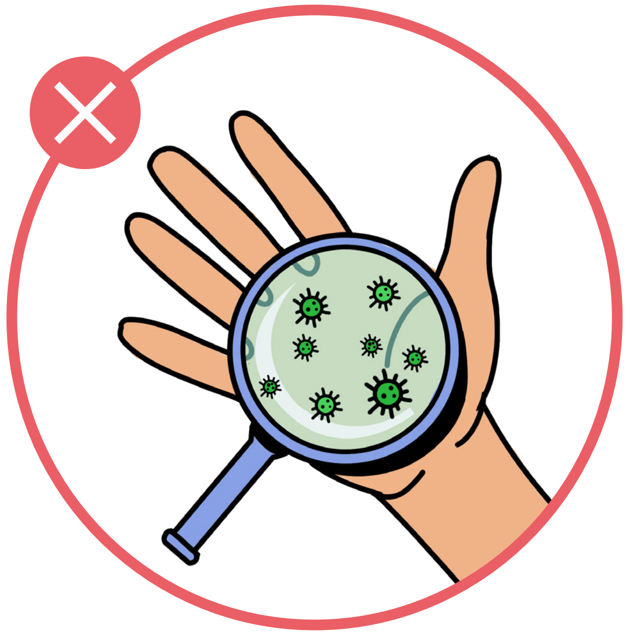

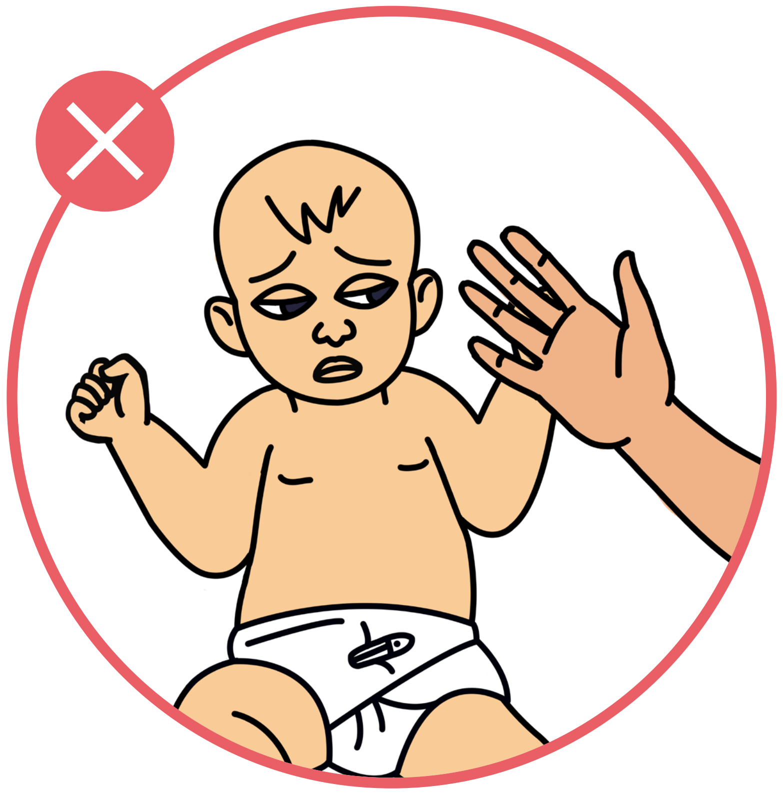

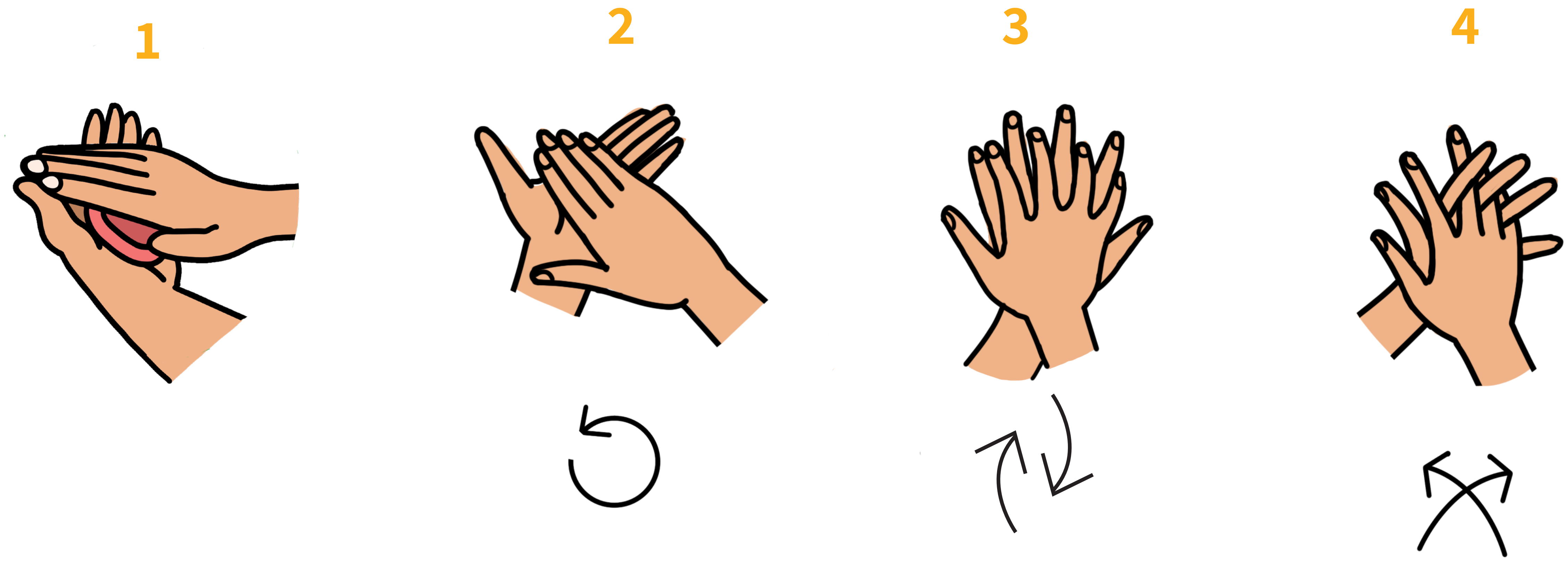

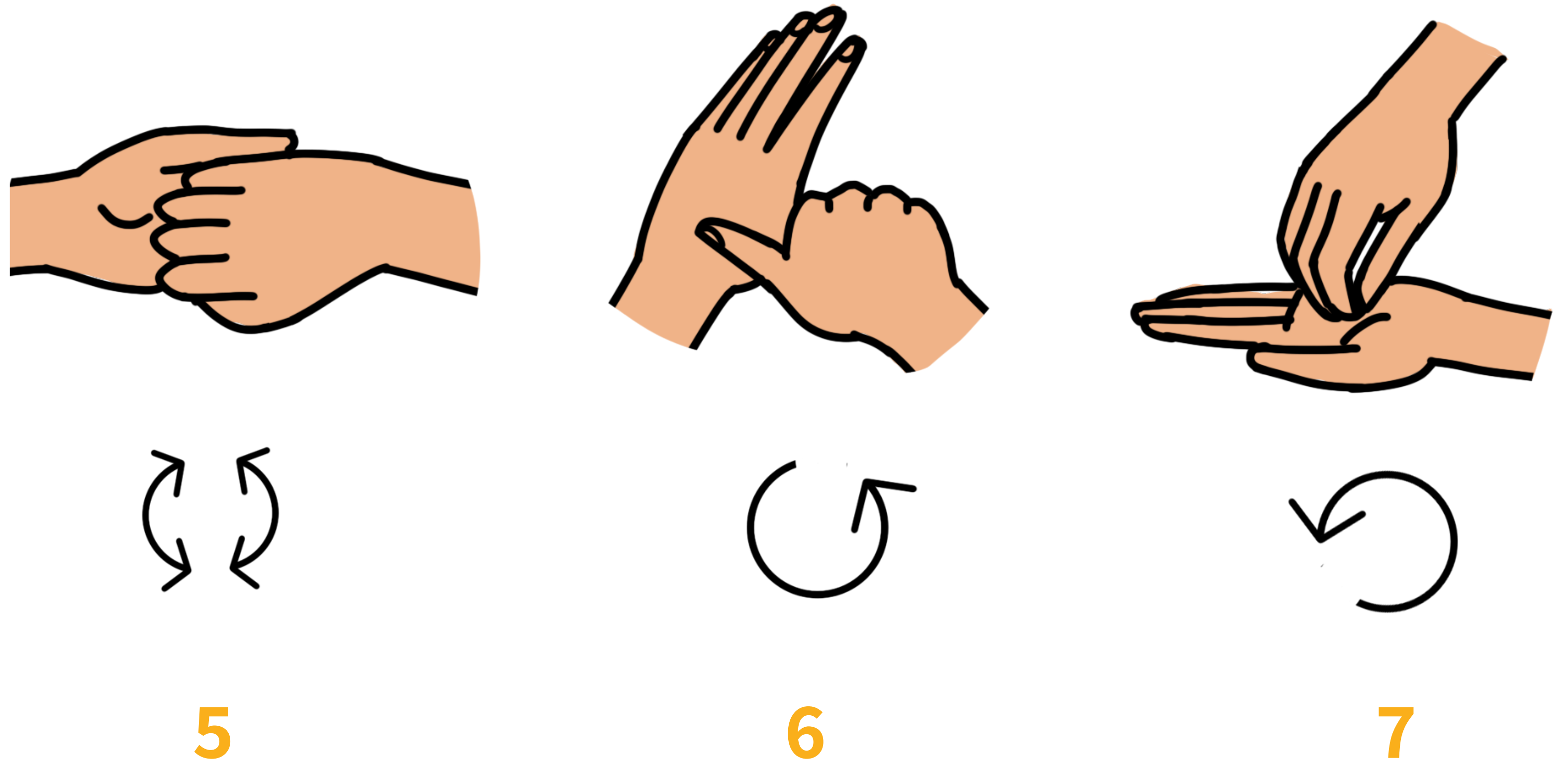

8. Handwashing

NOORA HEALTH

Can your hands make the baby sick?

- Yes. You touch people and surfaces throughout the day. Invisible germs may stick onto your hands
- Diseases can spread to the baby through your dirty hands
- The immunity of babies is not fully developed yet. They are at a higher risk of becoming sick

To prevent infections

- Wash your hands regularly with soap and water for at least 20 seconds

When to wash hands

Before

- Touching your baby
- Feeding your baby
- Cooking and eating

After

- Cleaning the baby
- Using the toilet
- Coughing or sneezing

Handwashing is the best and cheapest weapon to prevent infections.

Your baby's health is in your hands!

Demonstration

(Ask participants to follow the steps)

Rub soap and water to make foam.
Use this short form to remember the steps:
S U M A N -K (सुमन-क)

S = seedha
U = ulta
M = madhya and mutthi
A = angootha
N = nakhoon
K = kalai

Wash your hands. Dry them with your own clean towel or dry in the air.

(Ask participantsto re-demonstrate)

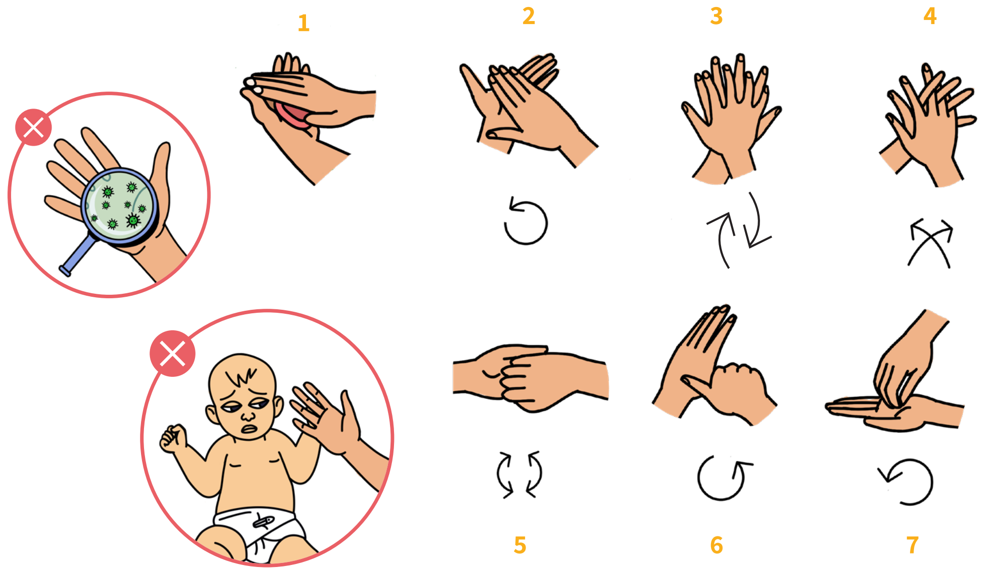

9. KMC / Skin to skin care

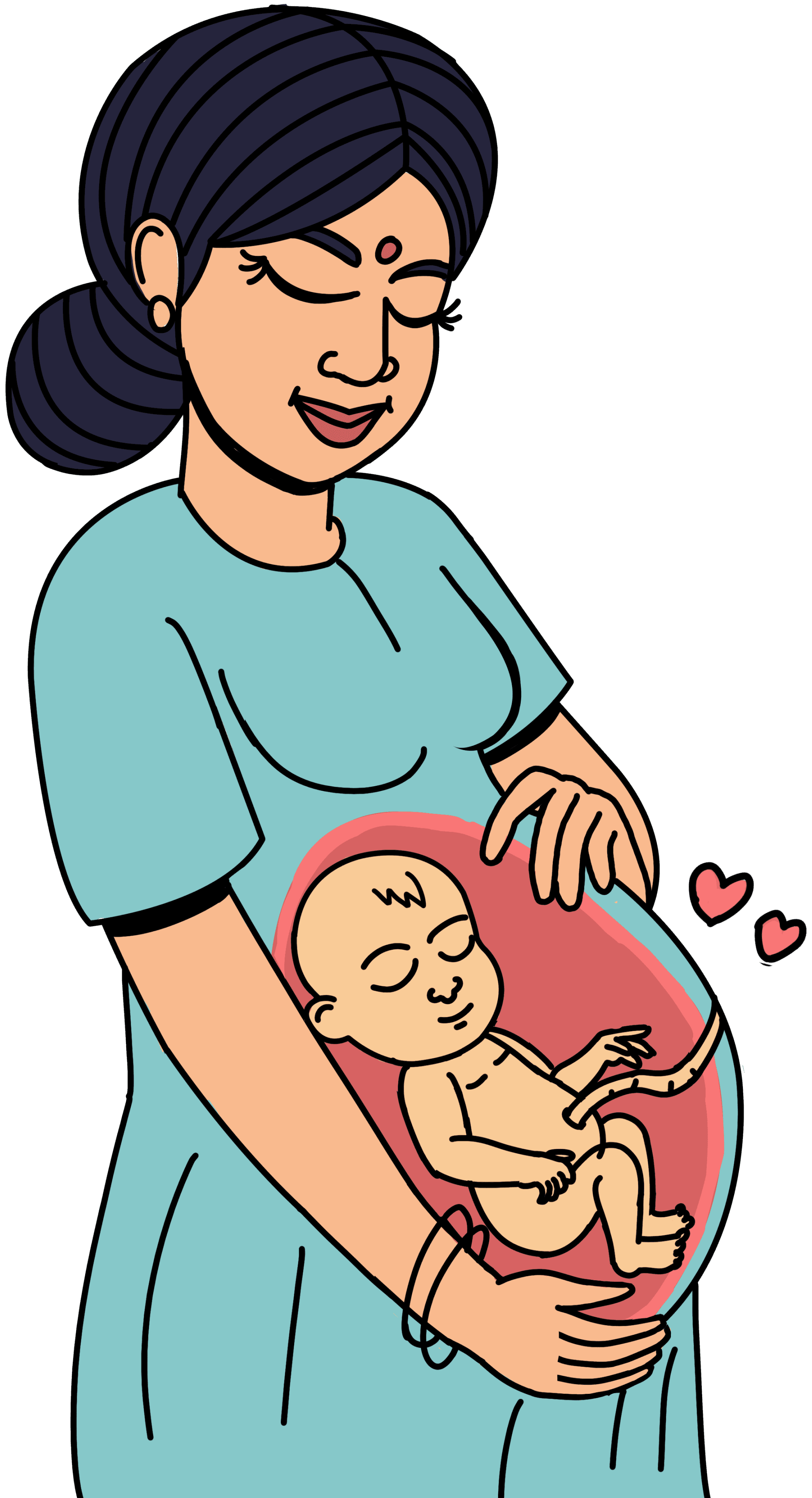

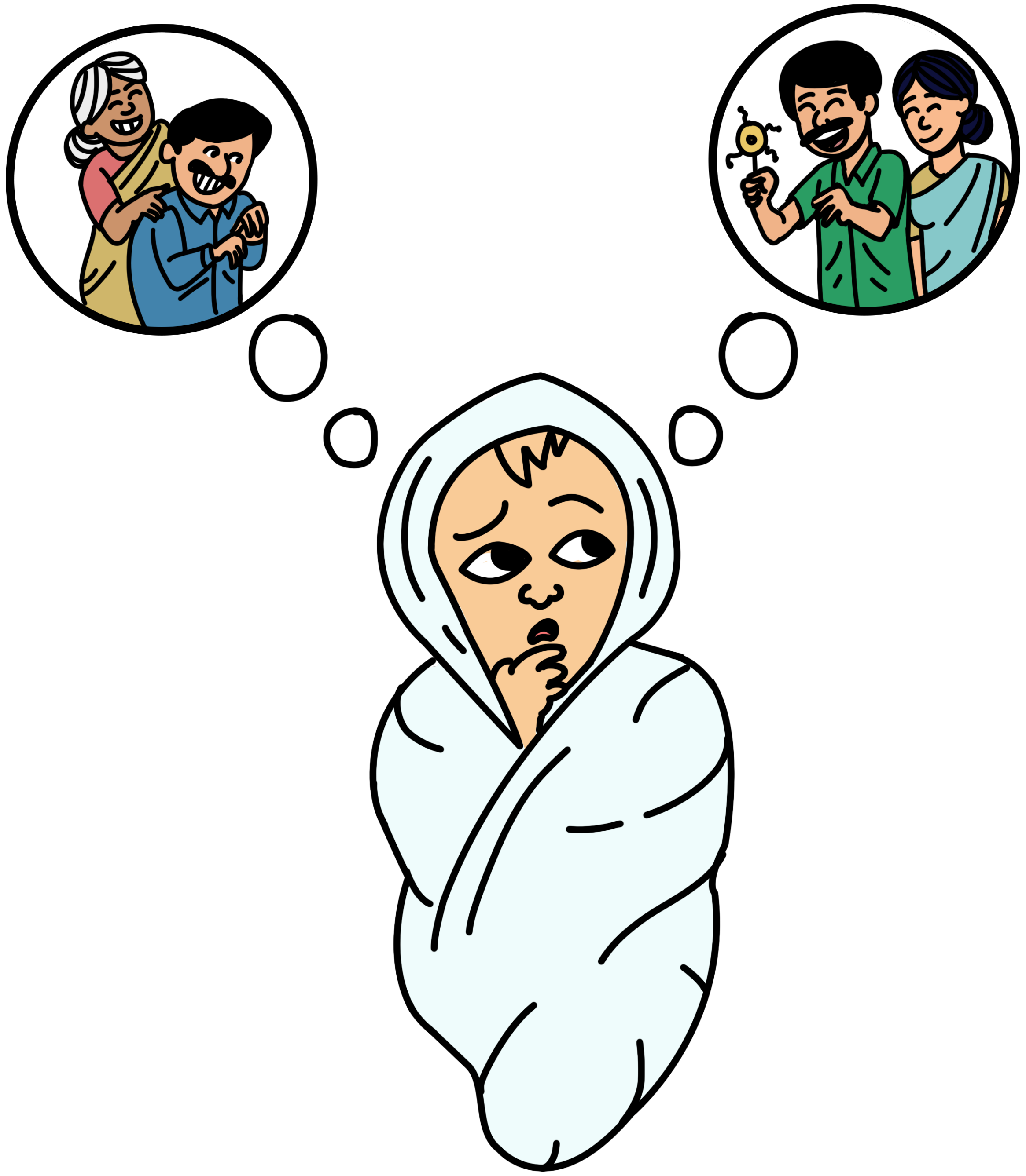

9. KMC / Skin to skin care

Before we move on to the next topic,

Please take a deep breath and close your eyes till I ask you to open it. I'll also do this with you. Close your eyes and focus on your breathing.

Wait For 20 Seconds

Now slowly open your eyes.
When I opened my eyes after shutting it for some time, light hit my eyes suddenly. I felt uncomfortable.
How many of you felt the same? Lift your hands.

Wait for the patients to answer. It's okay if they don't

Now imagine your baby, who was in your womb for 9 months.
Your baby was used to the sound of your heartbeat.

Point at a comfortable baby in the womb illustration

All of a sudden it is introduced to the cold outside world.
Your baby experiences everything for the first time, new smell, new people, new air, new food.

Points at baby outside the womb picture

Suddenly, the environment changes and your baby isn't comfortable.
When we are uncomfortable and a loved one holds our hand or hugs us or pats our back, it gives us confidence, makes us feel loved. It's the same with your baby. They need our **human touch** as well. You can give that comfort by doing skin to skin care or KMC helps your baby feel safe and warm.

By holding your baby naked close to your heart helps create an environment similar to the womb.

Now a question for everybody,
Who do you think should provide KMC to the baby?

Wait for the patients to answer. It's okay if they don't say anything

KMC can be provided by any adult in your family, for example the baby's father, grandmother, grandfather, uncle, aunt, the list goes on.

It's a responsibility that should be shared amongst everybody.

It helps the baby bond with other people in the family.

9. KMC / Skin to skin care

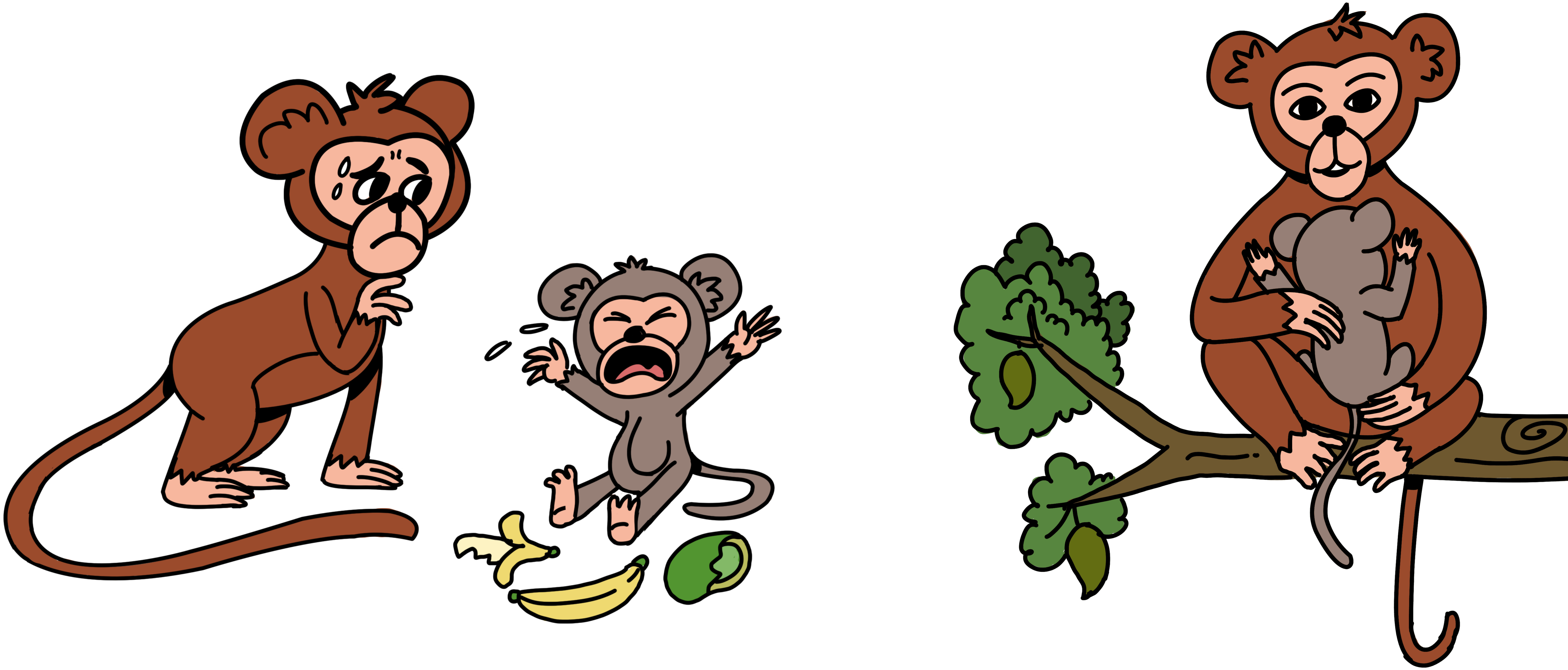

9. KMC / Skin to skin care

Long long time ago, our ancestors used to practice KMC with their babies to help them survive better.

So this is not a new technique I'm introducing you to.
With a lot of changes in our society and lifestyle we forgot about this.
But don't worry, we will bring this back. Together.

Point out at the mama monkey and baby monkey sitting picture

As you can see in this picture
that the mother is wondering what to do with her baby

Point out at the mama monkey and baby monkey hugging picture

Here in the next picture, you can see how the baby and mother feel when they are doing skin to skin care.

You must have noticed monkeys usually have their babies close to their bodies to give the babies and themselves a sense of comfort and security.

So are you ready to see how it is done?

Who is ready to volunteer for a demonstration?

How to prepare?

1. Find a quiet and separate place
2. Sit comfortably on a chair and recline your back
3. Open the front buttons of your gown/blouse/shirt/kurta
4. Keep a shawl to cover yourself

Demonstrate using a doll

Steps of KMC

1. Keep a cap, socks and nappy on the baby
2. Hold her/him on the middle of your chest
   Remember- Baby's skin should touch your skin
3. Turn the baby's head inwards and facing you. Make eye contact
4. Spread out Baby's arms and legs
5. Put your arm below the baby to support
6. Wrap both you and the baby for more support

When to practice skin-to-skin care?

Practice daily for as long as you can, upto 20 hours in a day
Family members can do it anytime

Mothers can try doing it after every breastfeeding for half an hour.

KMC helps all babies. Do Skin to Skin Care to make your baby happy and smart! This is the best gift a family can give the baby!

10. Diet and Healthy Behaviors for The Mother

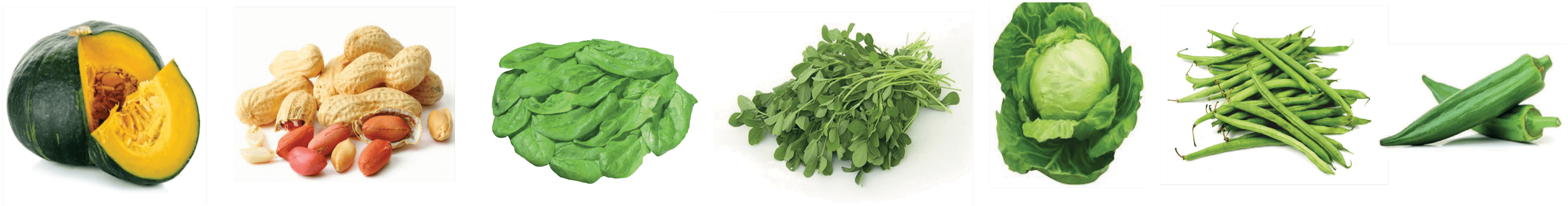

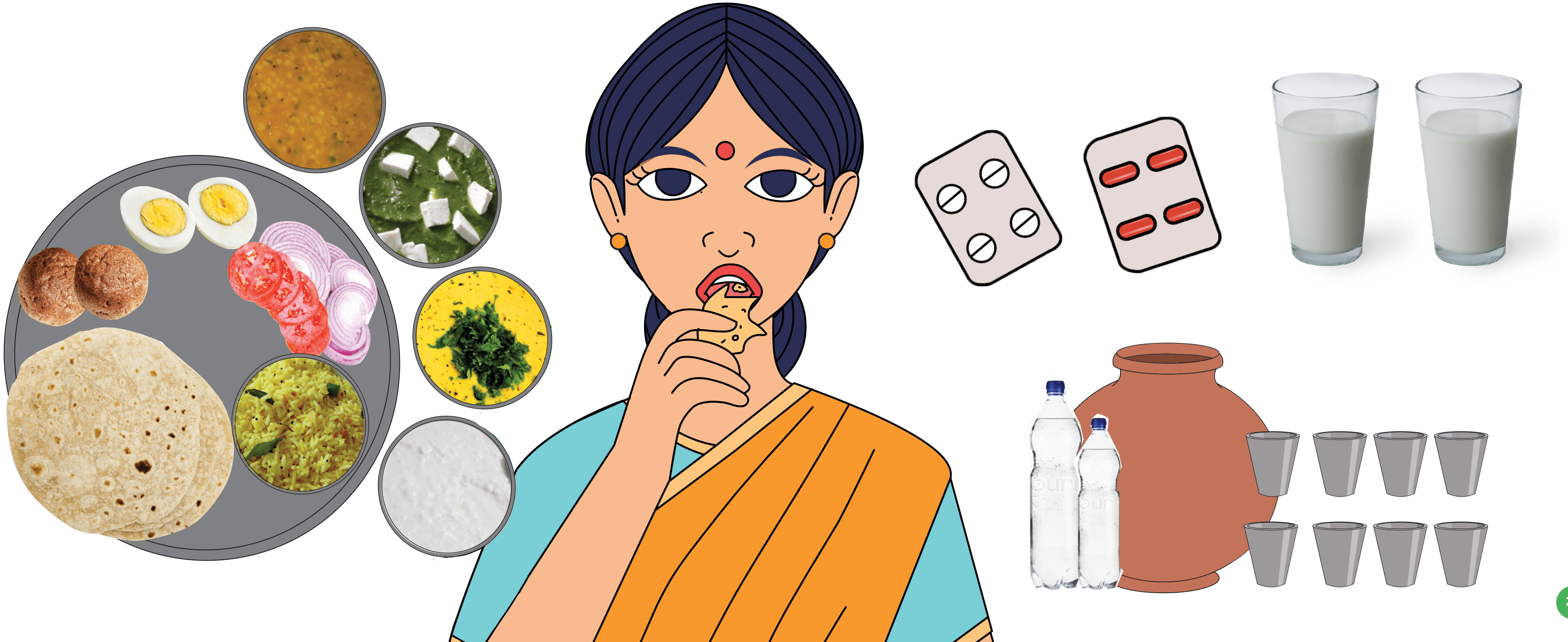

10. Diet and Healthy Behaviors for the Mother

NOORA HEALTH

(Pass the thali to participants)

What should the mother eat?

- Eat a nutritious diet having vegetables, fruits, chapati, rice, dal, rajma, milk, curd, paneer or egg
- Drink **at least 2 litres (8-10 glasses) of water** in addition to other liquids
- Adequate food and water is important for milk production
- Take **iron and calcium** tablets till 6 months after delivery

Healthy practices for better recovery

- Take a bath daily
- Take adequate rest
- Use sanitary pads and change regularly
- Keep the wound area clean and dry
- Get check-ups as advised

Mother needs more food than what she was eating during her pregnancy.

Question

(Read and ask participants to raise their hand)

If a mother drinks more water, will her milk get watery?

We understand there are several customs about mothers' diet.

What we are telling you, are based on scientific facts and are good for mother's and baby's health.

- Don't restrict the quantity of food
- Don't restrict water
- Don't restrict any particular food

Follow these proudly and confidently!

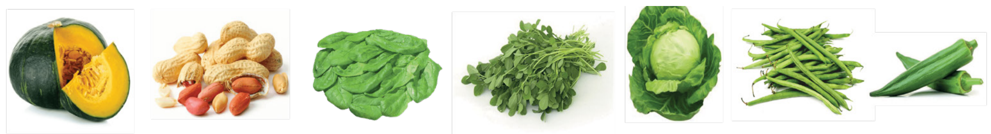

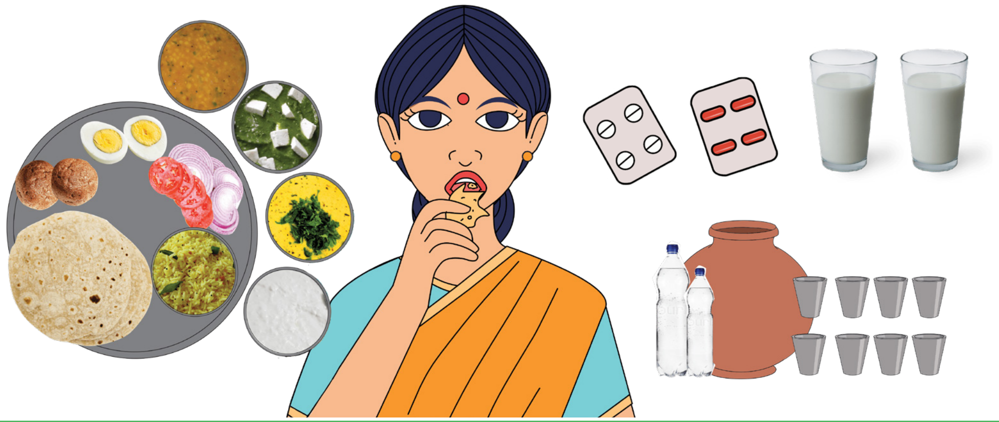

11. Follow Up for Mother and Baby

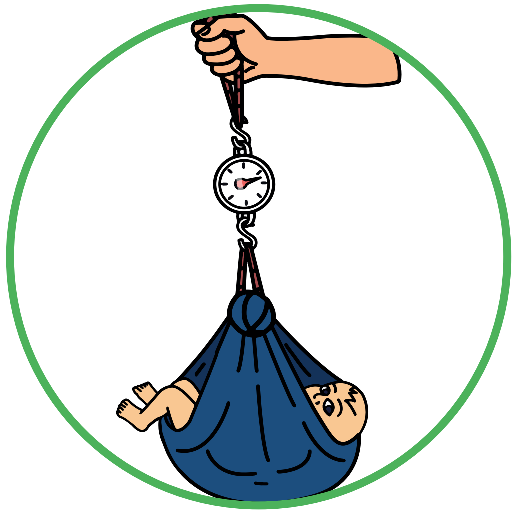

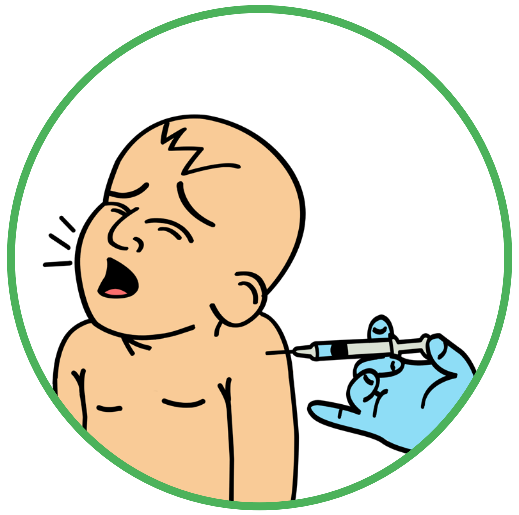

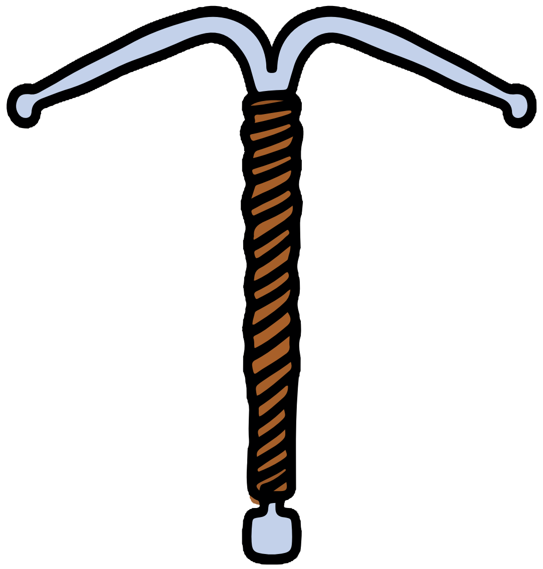

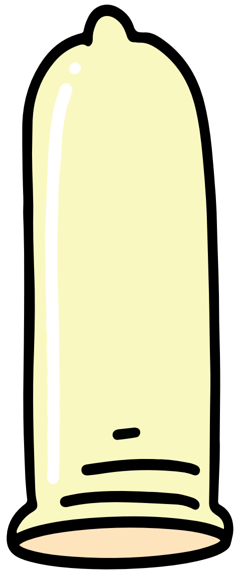

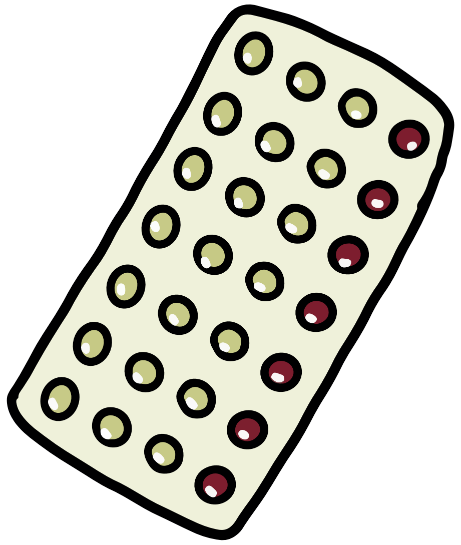

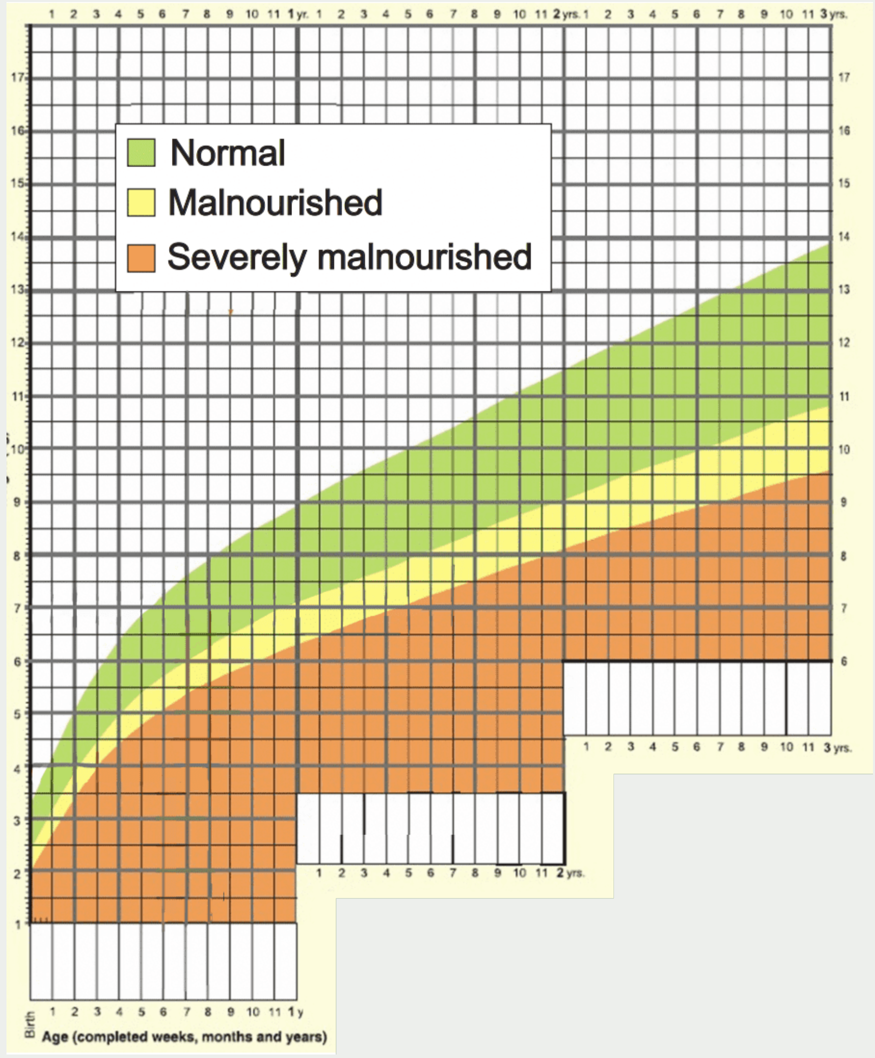

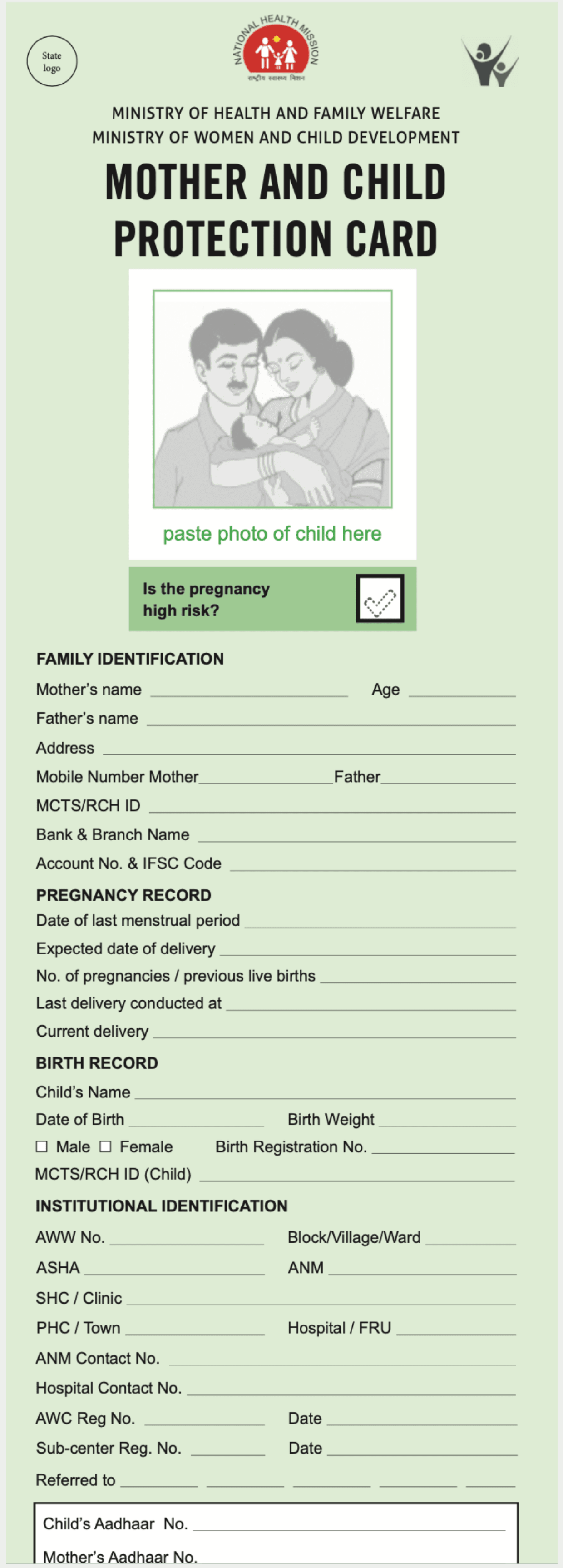

State logo

MINISTRY OF HEALTH AND FAMILY WELFARE
MINISTRY OF WOMEN AND CHILD DEVELOPMENT

MOTHER AND CHILD PROTECTION CARD

paste photo of child here

Is the pregnancy high risk?

FAMILY IDENTIFICATION

Mother's name ______ Age ______
Father's name ______
Address ______
Mobile Number Mother ______ Father ______
MCTS/RCH ID ______
Bank & Branch Name ______
Account No. & IFSC Code ______

PREGNANCY RECORD

Date of last menstrual period ______
Expected date of delivery ______
No. of pregnancies / previous live births ______
Last delivery conducted at ______
Current delivery ______

BIRTH RECORD

Child's Name ______
Date of Birth ______ Birth Weight ______
☐ Male ☐ Female Birth Registration No. ______
MCTS/RCH ID (Child) ______

INSTITUTIONAL IDENTIFICATION

AWW No. ______ Block/Village/Ward ______
ASHA ______ ANM ______
SHC / Clinic ______
PHC / Town ______ Hospital / FRU ______
ANM Contact No. ______
Hospital Contact No. ______
AWC Reg No. ______ Date ______
Sub-center Reg. No. ______ Date ______
Referred to ______

Child's Aadhaar No. ______
Mother's Aadhaar No.

Follow Up for Mother and Baby

Why are follow ups needed?

- For early detection of problems
- Giving vaccines, medicines and family planning advice
- Checking growth and weight of baby

Where to go?

Either ASHA/ ANM visits your home or you can go to the hospital.

When to go?

- Day 3
- Day 7
- 1.5 months

Additional visits are needed in LBW, premature and other problems with mother or baby.

Family planning

- Do you know that you can conceive even if you are breastfeeding!
- Wait for atleast 6 weeks before sexual activity
- Use contraceptive methods

Why vaccinate?

- Vaccination protects your baby against many diseases
- They are safe
- **Available free of cost in nearby government centers**

Vaccination saves your baby

Demonstration

(Ask participants to open the MCP card)

- Show the date for next vaccination
- Show growth chart. Understand if your baby is growing well

Green - Growing well
Yellow- Some problem
Orange- Severe problem

Keep it safe and with you during follow up.

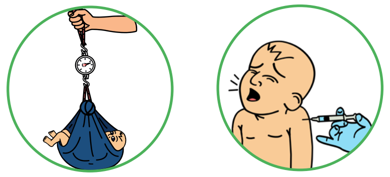

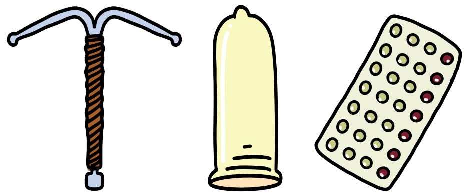

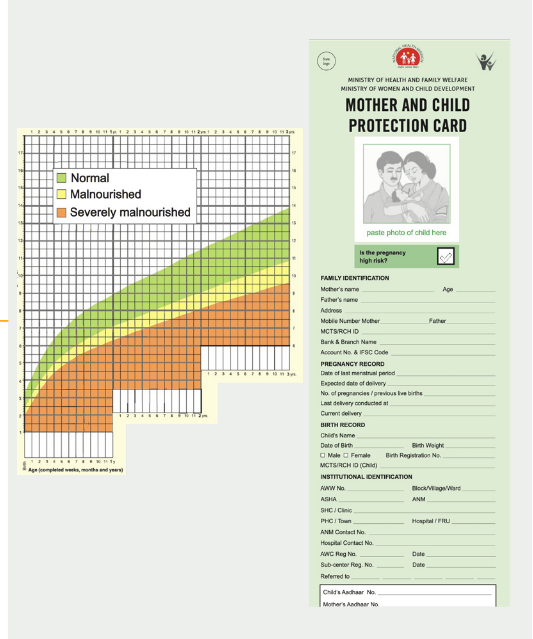

WhatsApp Onboarding

NOORA HEALTH

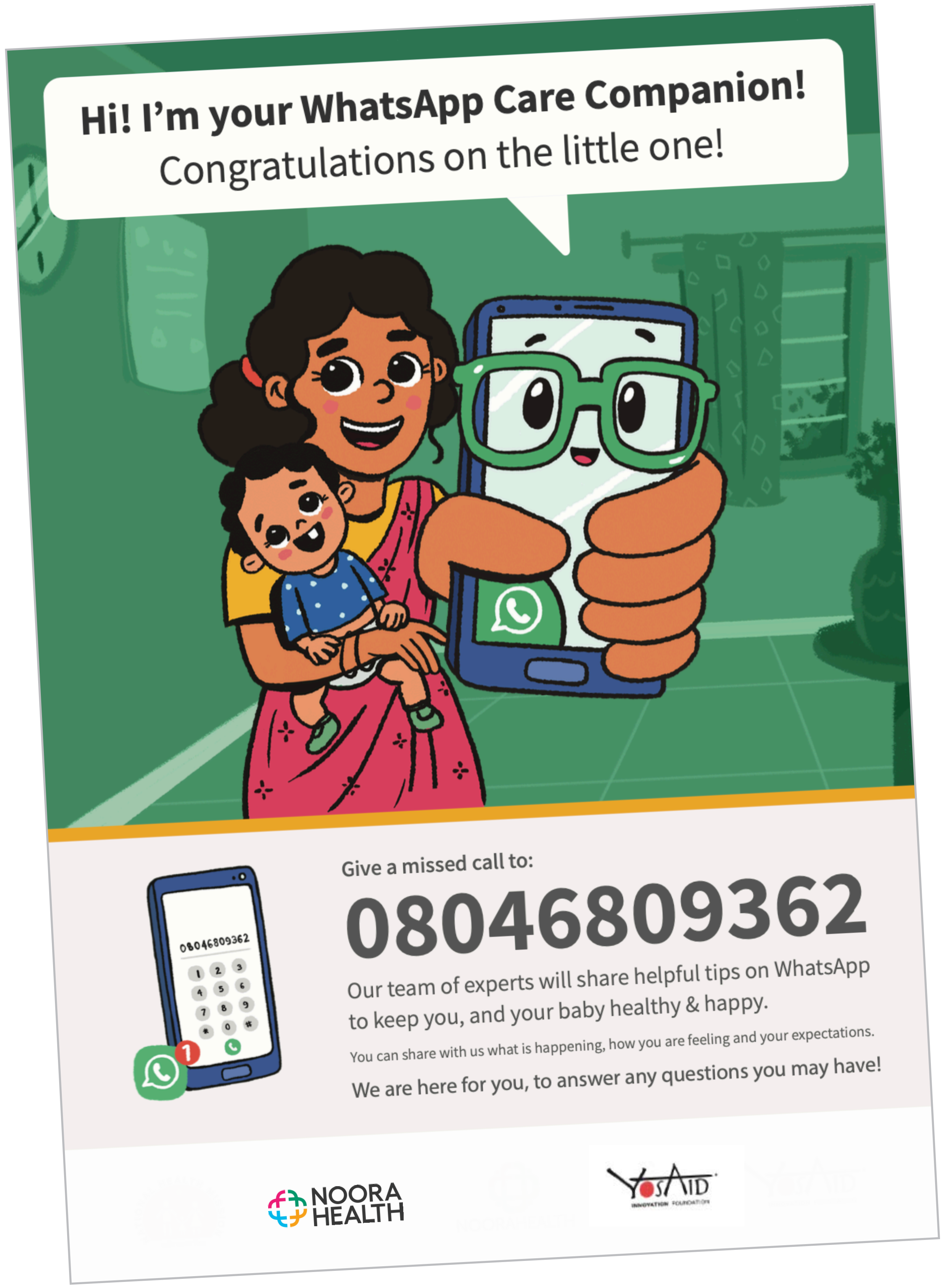

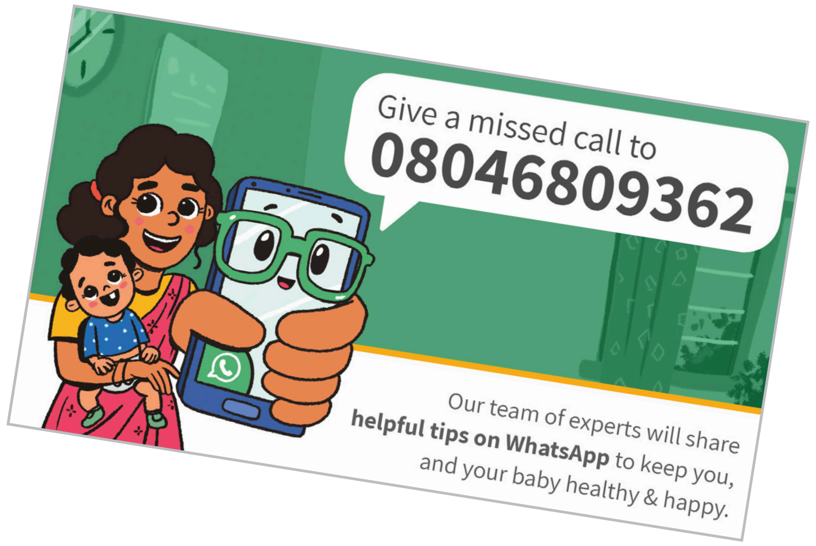

WhatsApp Onboarding

So far we have discussed the importance of giving only breastmilk till 6 months and breastfeeding even if mother or baby is sick, burping, keeping the cord dry, hand washing before touching the baby, how to identify danger signs and the importance of follow up.

Now, we have something more!

Do you want to receive
health advice about mother and baby's health?

Do you want any of your questions
answered after you go home?

We are here to support the free Whatsapp service.

This is not for emergencies

How to avail?

Step 1
Call 08046809362 from your registered WhatsApp number

Step 2
Wait for 3 sec

Step 3
Check for SMS

Step 4
Check for Whatsapp message

Ask your questions on whatsapp!

You will receive our health messages every alternate day for 27 days.

There will now be a demonstration and discussion around breastfeeding and other complications mothers may have after delivery. Request all male members to leave and mothers and female caregivers to stay back.

DISCUSSION SECTION

DISCUSSION SECTION

Maternal Complications and Less Milk

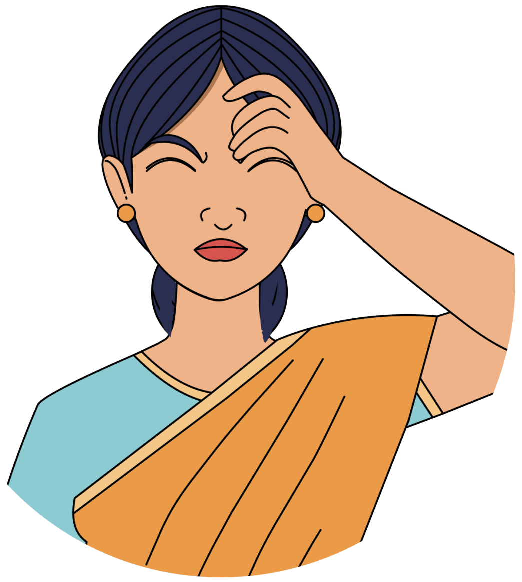

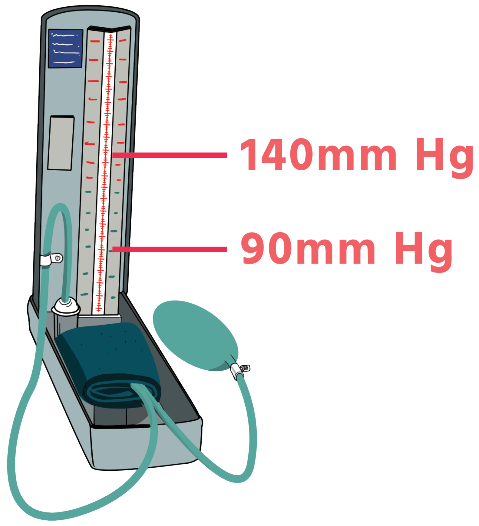

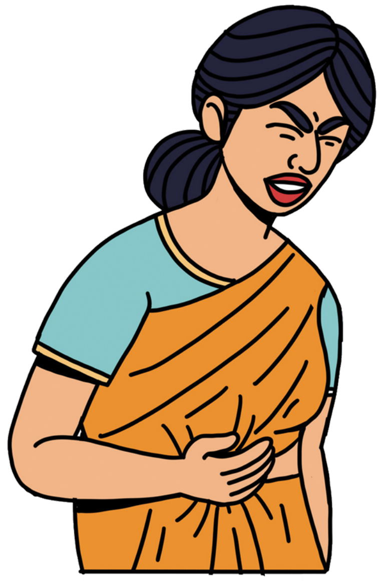

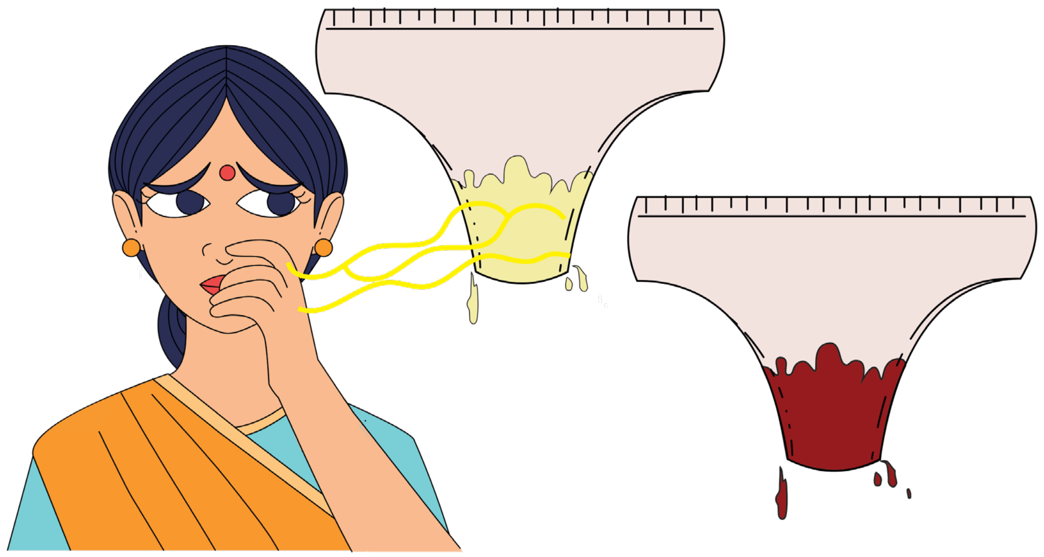

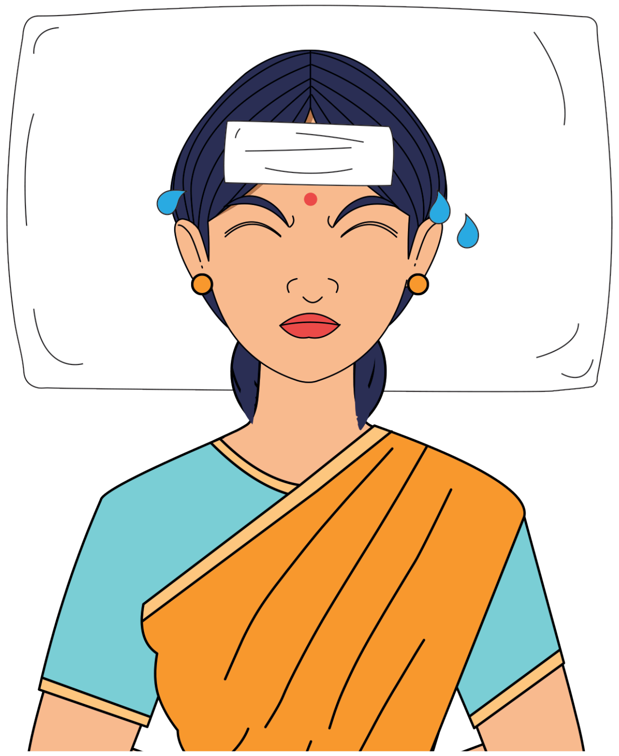

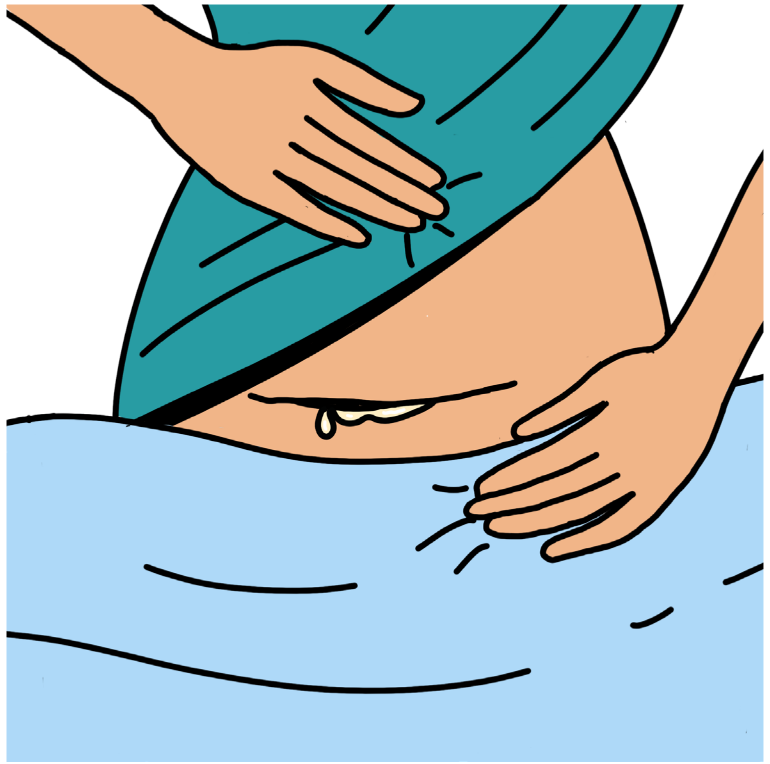

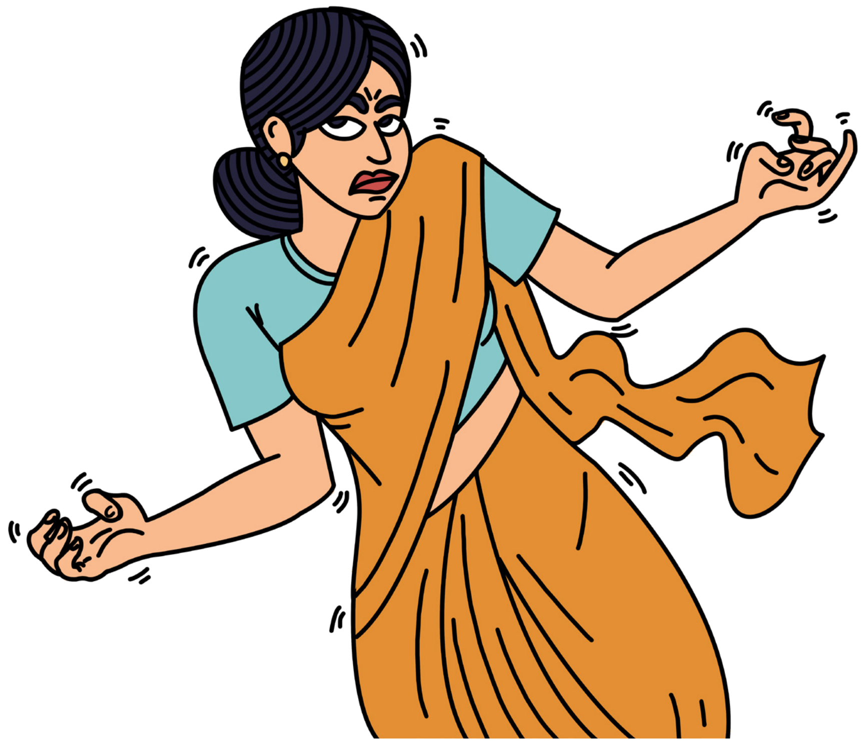

1. Maternal Complications and Less Milk

NOORA HEALTH

A. Maternal Complications: Show the pictures and ask participants to identify these danger signs one by one.

1. Excessive vaginal bleeding
2. Severe abdominal pain
3. Headache, dizziness,
4. Fits
5. Fever
6. Breast problems
7. Problem in stitches - Pain, redness, pus discharge, gaping, swelling.
8. Vomiting
9. Burning sensation in urination
10. Excessive tiredness, loss of appetite

- **Many mothers feel sad, have mood swings, and difficulty sleeping after childbirth**
- **You should not hide this**
- **Please discuss this during follow up visits**

B. Less Milk: Breast milk flow starts at delivery and increases by 3-4 days.

For good milk production

- Breastfeed frequently looking at baby's eyes while feeding
- Feed using the correct technique
- Be relaxed while feeding
- Feed from one breast fully before shifting to another breast
- Do skin to skin care frequently
- Eat well and drink **at least 2 litres (8-10 glasses) of water** in addition to other liquids

Consult the doctor if there is any problem.

Remember:

✗
- Don't worry unnecessarily. Milk will increase as the baby sucks
- Don't think that milk is not enough

✓
- The amount of milk produced by every mother is usually sufficient for the baby
- Being confident is important
- If your baby is active and growing well, you are breastfeeding successfully. **Congratulations!**

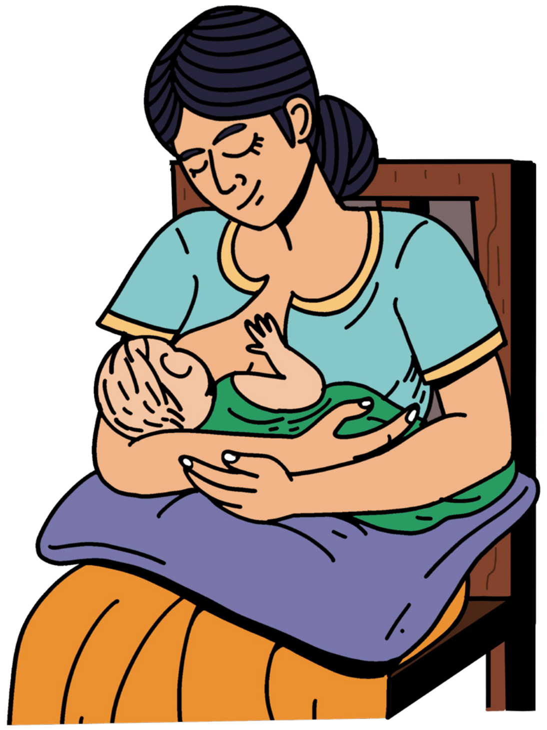

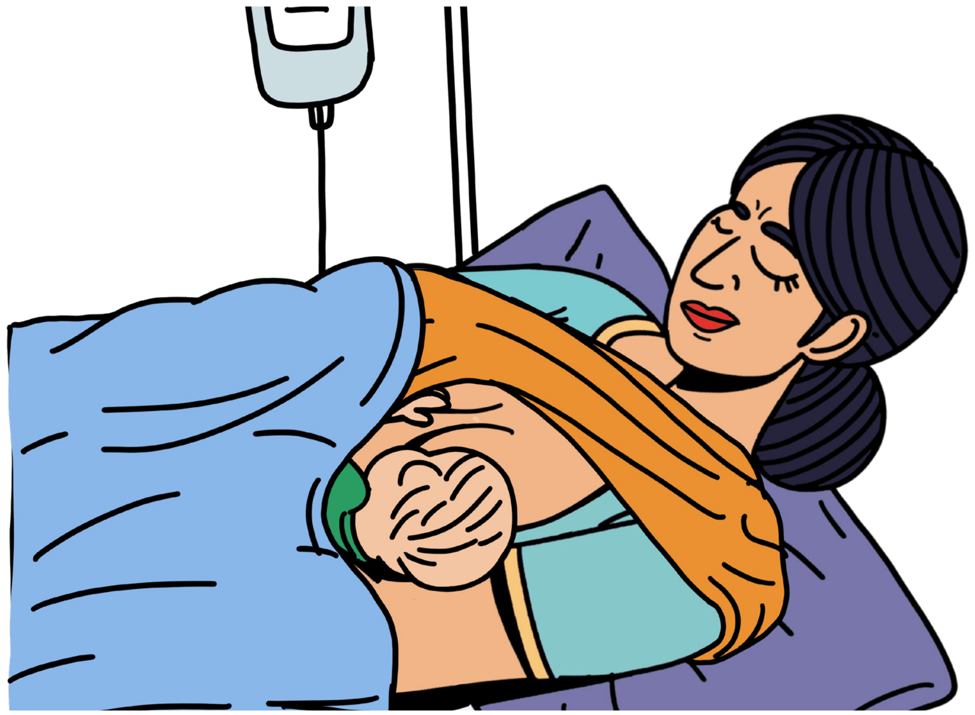

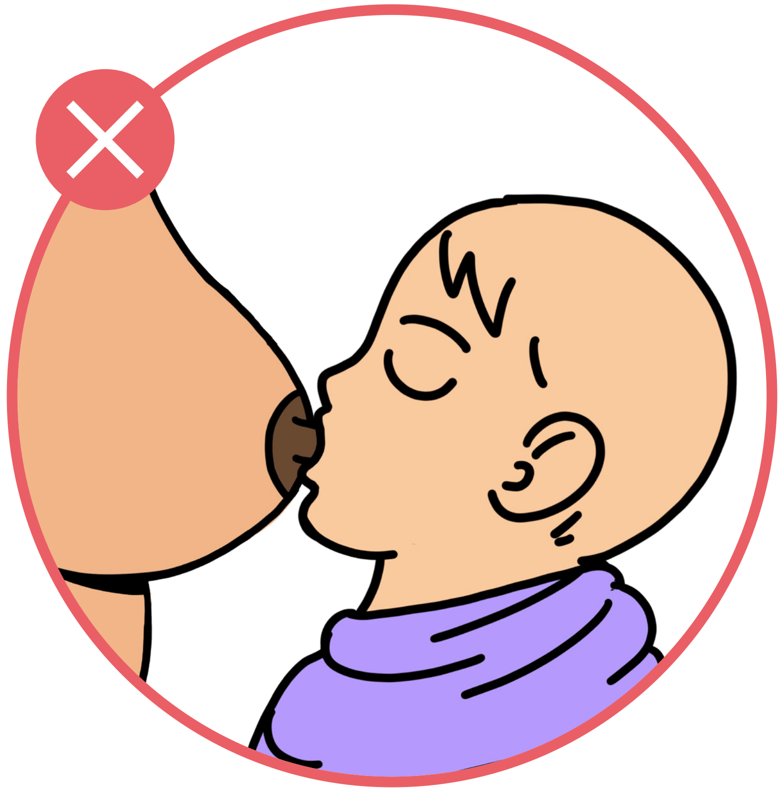

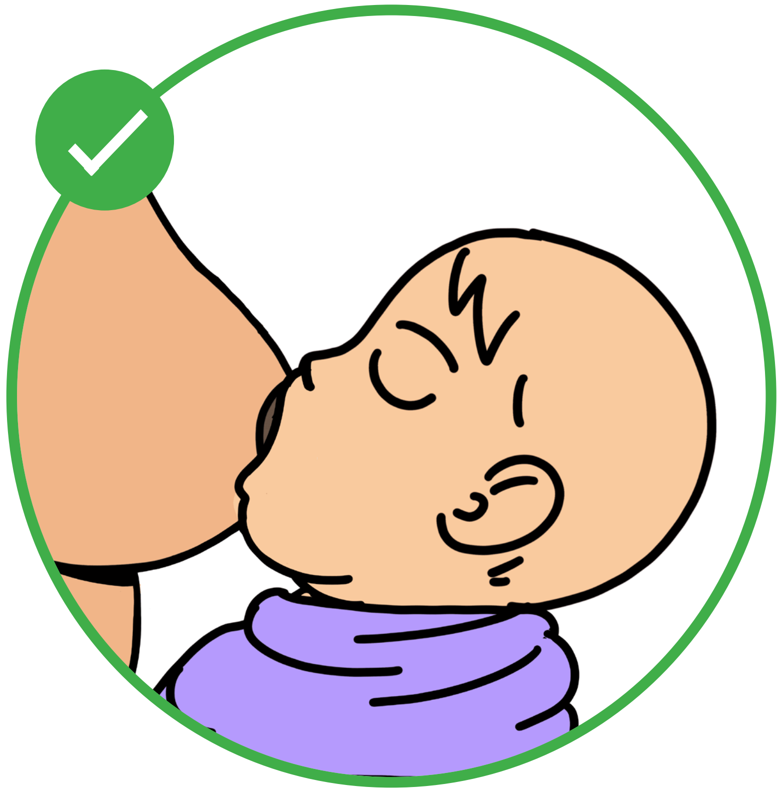

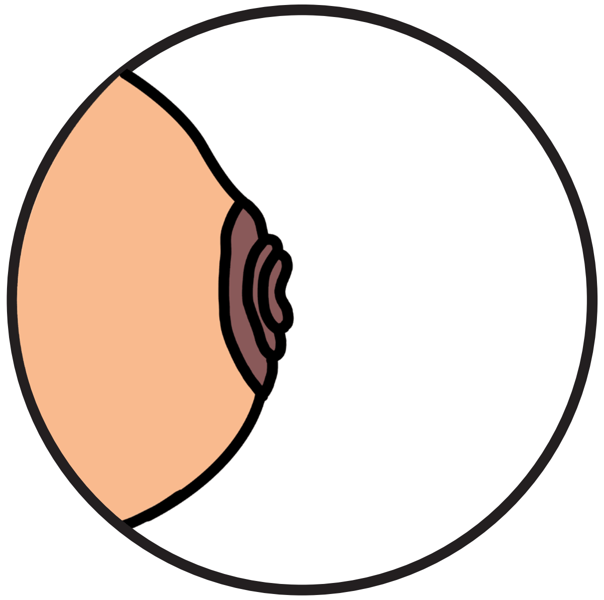

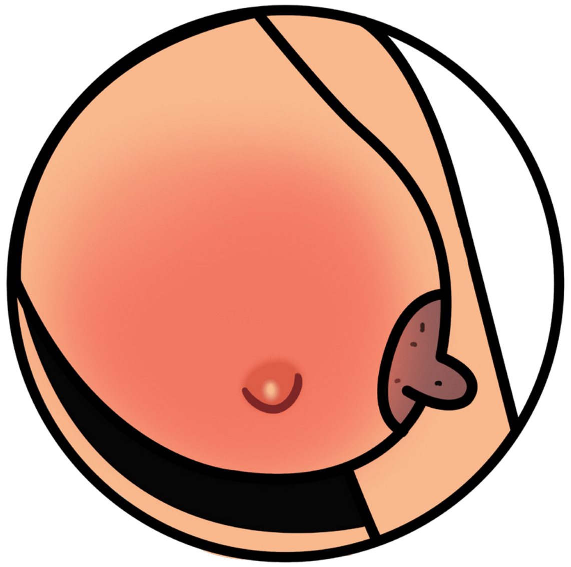

2. Correct Way to Breastfeed and Breast Problems

C. Correct Technique for Breastfeeding

Demonstrate using breast model /pictures/both

✓ **Baby holds the mother's breast with**

- Mouth **wide open**
- **Nipple and areola is inside** baby's mouth
- Lower **lip is outwards**
- Nose **is free**

✗ **If the areola of breast is seen**

- Baby will suck the nipple only and **will not get milk properly**
- Mother's nipple may get injured

Points to remember

- **Feed from both breasts each time**
- Feed from one breast till it is empty, then switch
- Burp the baby before switching

D. Correct Position for Breastfeeding

Demonstrate using doll/ pictures/ both

✓ **Correct position**

- Sit comfortably on a chair or bed
- Be relaxed and comfortable
- Put pillow on your lap
- Support baby's neck with arm, while baby lies on the pillow

✗ **Do not bend forward over the baby**

Points to remember

- **Feed the baby either sitting or lying down**
- A sitting position is preferable
- If feeding while lying down, **keep the baby's head up**

E. Problems in Breast

Examine your breasts daily to see if they are soft without any lumps and there are no cracks in the nipples.

1. Inverted nipples

- Nipples may naturally be flat or inverted
- Difficult to feed the baby

What to do?

- Use your hand to gently pull out your nipples, while taking bath
- Use a cut syringe and gently pull out the nipple for a few days

2. Cracked, painful or bleeding nipples

Nipples can get injured if using the wrong technique for breastfeeding

What to do?

- Feed using correct technique
- Don't let the baby continue to suck after the breast is empty. Release suction by pressing down the baby's chin
- Apply some breast milk on the nipples and let it dry
- If it is very painful, stop breastfeeding for 1 day. Hand express and feed
- Consult a doctor, if severe

3. Engorged breasts

- Breasts become full, tight and painful. There may be hard lumps or fever
- Happens when the baby's feeding is reduced

What to do?

- Apply a towel soaked in warm water over breasts
- Hand express some milk before feeding
- Feed fully from both breasts
- Feed when baby is hungry using correct technique
- Consult a doctor, if severe

3. Hand Expression of Breastmilk

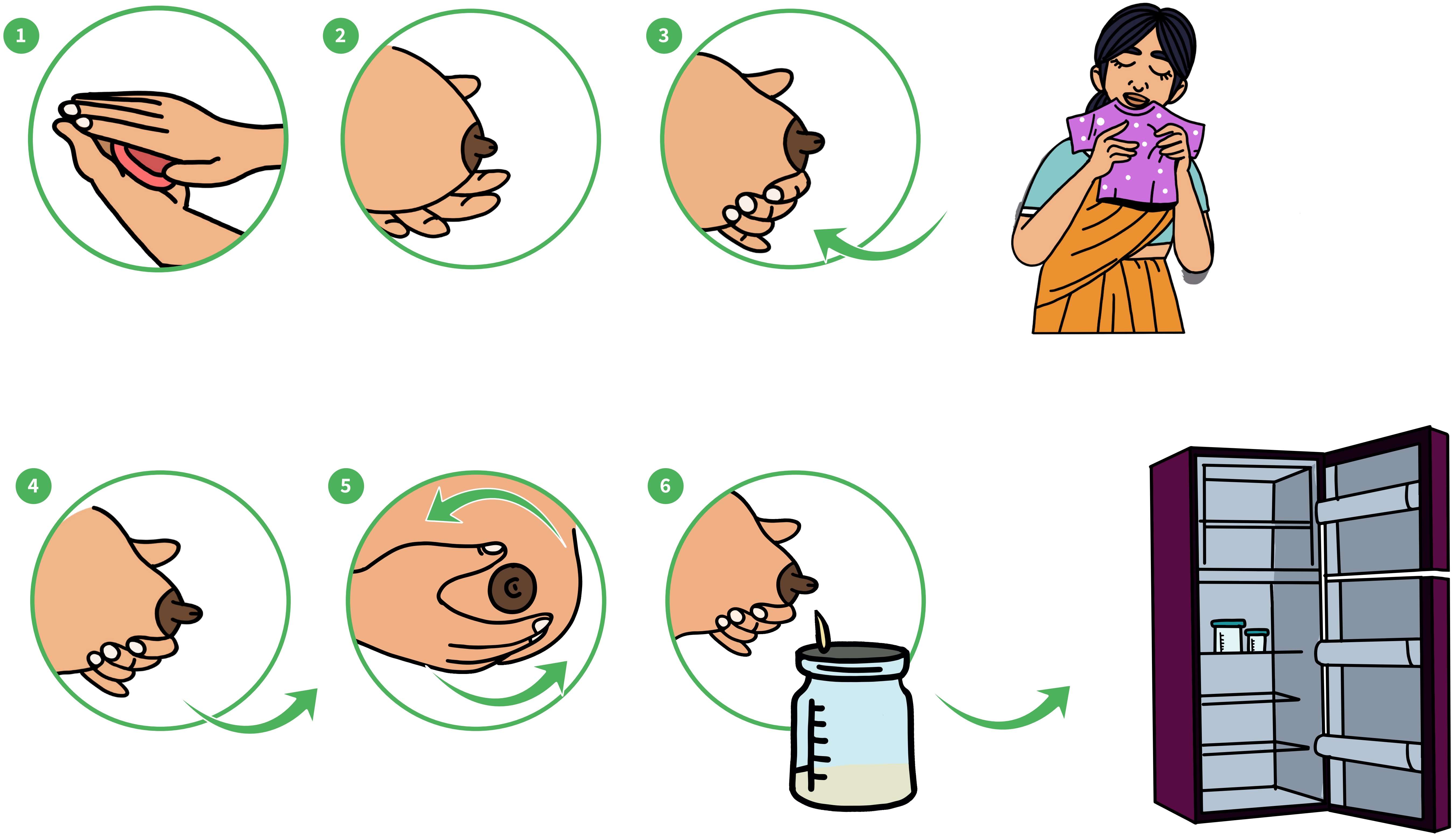

3. Hand Expression of Breastmilk

F. Hand expression of breastmilk:

When?

- Baby cannot suck or very sick
- Milk flow not yet started

What?

- Simple process
- Can be done by mother without a machine
- Do this gently to avoid pain

Prepare

- Wash hands with soap and water
- Take a clean container
- Look at the baby's photo or smell its clothes

Points to remember:

- **Do this 6- 8 times every day. At night too**
- Express the milk from all parts of the breasts

- If you let milk collect in breasts, you may have severe pain and infection
- If you delay starting milk flow from breasts, you may have no milk flow later

Emptying the breast will help the breast to produce milk again and again.

Demonstrate

Use a breast model and show the steps

1. Place your thumb above the nipple and the first two fingers below, in a 'half moon' shape
2. Push your thumb and fingers backwards
3. Bring them forwards
4. Then press them and squeeze the milk into the clean container
5. Do this rhythmically (backwards- forwards - press)

Feed this to baby within 6 hours, using bowl and spoon
