# Supplementary material for: Qualitative assessment of family caregiver-centered neonatal education program in Karnataka, India
Source: PLOS Glob Public Health. 2023 Feb 14;3(2):e0000524. doi: 10.1371/journal.pgph.0000524 (PMC10022017; doi:10.1371/journal.pgph.0000524)
Supplement: S2 Text — (PDF) [file pgph.0000524.s002.pdf]

Interviewer Guide: Women

ಸಂದರ್ಶಕರ ಸೂಚಿ: ಮಹಿಳೆಯರಿಗೆ

Interviewer:<br>ಸಂದರ್ಶಕರು:	Note taker:<br>ಬರೆದುಕೊಳ್ಳುವವರು :
Start time (hh:mm): ಆರಂಭದ ಸಮಯ	End time (hh:mm): ಮುಕ್ತಾಯದ ಸಮಯ
Recorder file name: ರಿಕಾರ್ಡರ್ ಫೈಲ್ ಸಂ.	Date (dd/month/yyyy): ದಿನಾಂಕ: (ದಿ/ತಿಂ/ವ)

Introduction: ಪರಿಚಯ

Hello, and thank you for meeting with us today. My name is_________ [INTERVIEWER NAME] and I will be guiding our discussion today, I'm here with ________[NOTETAKER NAME] who will be taking notes so we can remember what was said.
We are here to talk about your experience with the group session you participated in at {hospital} and caring for your newborn baby. You have really important information and experience about caring for newborn babies and we want to learn from you. This conversation will help us understand and develop better education programs for taking care of babies and mothers.

During this discussion we might talk about personal issues, if you do not want to answer these questions, you do not have to. Please remember that there are no right or wrong answers, we just want to hear about your experiences and opinions. If a question or word is unclear, please let me know and I will explain it. Today's discussion will last around 90 minutes and it will be audio recorded. We will not use your name or information about you on any documents. If you are not comfortable being audio recorded, please let us know.

ನಮಸ್ಕಾರ, ನಿಮ್ಮನ್ನು ಭೇಟಿಯಾಗುತ್ತಿರುವುದಕ್ಕೆ ಸಂತೋಷವೆನಿಸುತ್ತಿದೆ. ಈ ಭೇಟಿಗೆ ಅವಕಾಶ ಕೊಟ್ಟಿದ್ದಕ್ಕಾಗಿ ನಿಮಗೆ ಧನ್ಯವಾದಗಳು. ನನ್ನ ಹೆಸರು - [ಸಂದರ್ಶಕರ ಹೆಸರು]. ನಾನು ಇಂದು ನಿಮ್ಮ ಹಾಗೂ ನಿಮ್ಮ ಮಗುವಿನ ಆರೋಗ್ಯಕ್ಕೆ ಸಂಬಂಧಿಸಿದಂತೆ ಕೆಲವು ವಿಷಯಗಳನ್ನು ನಿಮ್ಮೊಂದಿಗೆ ಚರ್ಚೆ ಮಾಡಲು ಬಂದಿದ್ದೇನೆ. ಇಲ್ಲಿನ

ಚರ್ಚೆಯ ವಿಷಯಗಳನ್ನು ಬರೆದುಕೊಳ್ಳಲು ನನ್ನೊಂದಿಗೆ -[ಬರೆದುಕೊಳ್ಳುವವರ ಹೆಸರು] ಇದ್ದಾರೆ. ಇವರು ನಾವು ಹೇಳಿದ್ದನ್ನು ಬರೆದುಕೊಳ್ಳುತ್ತಾರೆ. ಇದರಿಂದ ನೀವು ಹೇಳಿದ್ದನ್ನು ನಾವು ನೆನಪಿಸಿಕೊಳ್ಳಬಹುದು

ಎಳೆಮಗು ಆರೈಕೆಯ ಬಗ್ಗೆ ಆಸ್ಪತ್ರೆಯಲ್ಲಿ ನೀವು ಭಾಗವಹಿಸಿದ್ದ ಕ್ಲಾಸ್ ಬಗ್ಗೆ ನಿಮ್ಮ ಅನುಭವ ಹೇಗಿತ್ತು ಎಂಬುದನ್ನು ತಿಳಿದುಕೊಳ್ಳಲು ನಾವು ಇಲ್ಲಿ ಬಂದಿದ್ದೇವೆ. ಎಳೆಮಕ್ಕಳ ಆರೈಕೆಯ ಬಗ್ಗೆ ನಿಮಗೆ ಇರುವ ಅನುಭವವನ್ನು ಮತ್ತು ಅದರ ಬಗ್ಗೆ ನಿಮಗೆ ಗೊತ್ತಿರುವ ಮುಖ್ಯವಾದ ಮಾಹಿತಿಯನ್ನು ತಿಳಿಯಲು ನಾವು ಬಯಸುತ್ತೇವೆ. ನಿಮ್ಮ ಅನುಭವ ಹಾಗೂ ನೀವು ನೀಡುವ ಮಾಹಿತಿಗಳ ಆಧಾರದ ಮೇಲೆ ಎಳೆಮಕ್ಕಳನ್ನು ಮತ್ತು ಬಾಣಂತಿಯನ್ನು ಯಾವ ರೀತಿ ನೋಡಿಕೊಳ್ಳಬೇಕು ಎನ್ನುವ ಬಗ್ಗೆ ಉತ್ತಮ ಶಿಕ್ಷಣವನ್ನು ಆಸ್ಪತ್ರೆಯಲ್ಲಿ ಆಯೋಜಿಸಲು ಸಹಾಯವಾಗುತ್ತದೆ.

ಈ ಚರ್ಚೆಯಲ್ಲಿ ನಾವು ಕೆಲವು ವೈಯಕ್ತಿಕ ಪ್ರಶ್ನೆಗಳನ್ನು ಕೇಳಬಹುದು. ಇದಕ್ಕೆ ಉತ್ತರಿಸಲು ನಿಮಗೆ ಇಷ್ಟವಿಲ್ಲದಿದ್ದಲ್ಲಿ, ನೀವು ಉತ್ತರ ಹೇಳಬೇಕಿಲ್ಲ. ಇಲ್ಲಿ ಯಾವುದೇ ಸರಿ ಅಥವಾ ತಪ್ಪು ಎನ್ನುವ ಉತ್ತರಗಳಿಲ್ಲ. ನಮಗೆ ನಿಮ್ಮ ಅನುಭವ ಮತ್ತು ಅಭಿಪ್ರಾಯ ಮಾತ್ರ ಮುಖ್ಯ. ಇದು ಸುಮಾರು 90 ನಿಮಿಷಗಗಳನ್ನು ತಗೆದುಕೊಳ್ಳುತ್ತದೆ ಮತ್ತು ಇದನ್ನು ರೆಕಾರ್ಡ್ ಮಾಡಿಕೊಳ್ಳುತ್ತೇವೆ. ನಿಮ್ಮ ಹೆಸರು ಅಥವಾ ಮಾಹಿತಿಯನ್ನು ನಾವು ಎಲ್ಲಿಯೂ ಪ್ರಕಟಿಸುವುದಿಲ್ಲ. ನಿಮ್ಮ ಮಾತುಗಳನ್ನು ರೆಕಾರ್ಡ್ ಮಾಡುವುದು ನಿಮಗೆ ಇಷ್ಟವಿಲ್ಲದಿದ್ದರೆ, ದಯವಿಟ್ಟು ನಮಗೆ ಹೇಳಿ,

Are there any questions before we start? I'm going to begin recording now.
ಈ ಸಂದರ್ಶನ ಪ್ರಾರಂಭಿಸುವುದಕ್ಕಿಂತ ಮುಂಚೆ ನಿಮಗೇನಾದರೂ ಅನುಮಾನಗಳಿದ್ದರೆ ದಯವಿಟ್ಟು ಕೇಳಿ, ಈಗ ನಾನು ರೆಕಾರ್ಡ್ ಮಾಡಲು ಶುರು ಮಾಡುತ್ತೇನೆ.

SECTION I ಭಾಗ I

First, I want to ask you some questions about how you felt before and after you gave birth at __________ [*insert name of hospital where mother gave birth*].<br>ಮೊದಲಿಗೆ, ನಿಮಗೆ ಹೆರಿಗೆಯಾದ .... ಆಸ್ಪತ್ರೆಯಲ್ಲಿ [ಹೆರಿಗೆಯಾದ ಹಾಸ್ಪಿಟಲ್ ಹೆಸರು ನಮೂದಿಸಿ] ಹೆರಿಗೆಗೆ ಮೊದಲು ಮತ್ತು ಹೆರಿಗೆಯ ನಂತರ ನಂತರ ನಿಮಗೆ ಹೇಗೆ ಅನಿಸಿತು ಎನ್ನುವ ಬಗ್ಗೆ ಕೆಲವು ಪ್ರಶ್ನೆಗಳನ್ನು ಕೇಳುತ್ತೇನೆ.
Question 1: Do you remember participating in any group sessions with a flipchart, videos, or dolls at [*hospital name*] after [*baby name*] was born?

ಮಗು ಹುಟ್ಟಿದ ನಂತರ .... ಆಸ್ಪತ್ರೆಯಲ್ಲಿ ಚಾರ್ಟ್‌ಗಳನ್ನು, ವೀಡಿಯೋಗಳು ಅಥವಾ ಗೊಂಬೆಗಳನ್ನು ಇಟ್ಟುಕೊಂಡು ನಡೆಸಲಾದ ಯಾವುದಾದರೂ ಗುಂಪು ಕ್ಲಾಸ್ ಲ್ಲಿ ಭಾಗವಹಿಸಿದ್ದೀರಾ?

- If participant needs a reminder: Do you remember if the nurse spoke about a whatsapp number you could give a missed call to?
  ಭಾಗವಹಿಸುವವರಿಗೆ ನೆನಪಿಸಬೇಕಾದರೆ: ಆಸ್ಪತ್ರೆಯ ನರ್ಸ್ ಯಾವುದಾದರೂ ವಾಟ್ಸ್ ಅಪ್ ನಂಬರ್‌ಗೆ ನೀವು ಮಿಸ್ ಕಾಲ್ ಕೊಡಲು ಹೇಳಿದ್ರಾ?

Question 2: What do you remember from the group session?
ಗುಂಪು ಕ್ಲಾಸ್ ನಲ್ಲಿ ಕಲಿತ ವಿಷಯಗಳ ಬಗ್ಗೆ ನಿಮಗೆ ಏನು ನೆನಪಿದೆ?

Question 3: What did you think about the group session? ಈ ಕ್ಲಾಸ್ ಬಗ್ಗೆ ನಿಮಗೆ ಏನನ್ನಿಸಿತು?

- [PROBES: What did you like/not like? How did you feel about having other members of your family in the session?]
  [ಕೇಳಿ: ನಿಮಗೆ ಏನು ಇಷ್ಟವಾಯಿತು? / ಇಷ್ಟವಾಗಲಿಲ್ಲ? ನಿಮ್ಮ ಮನೆಯ ಇತರ ಸದಸ್ಯರು ಈ ಕ್ಲಾಸ್ ನಲ್ಲಿ ಭಾಗವಹಿಸಿದ ಬಗ್ಗೆ ನಿಮಗೆ ಏನನ್ನಿಸಿತು?

Jump to complication and probe fully if participant begins talking them. For behaviors probe later.(ಭಾಗವಹಿಸುವವರು ಮಗುವಿನ ಆರೋಗ್ಯದ ತೊಂದರೆಗಳ ಬಗ್ಗೆ ಮಾತನಾಡಲು ಪ್ರಾರಂಭಿಸಿದರೆ, ಮಗುವಿನ ಆರೋಗ್ಯದ ತೊಂದರೆ ಸಂಪೂರ್ಣವಾಗಿ ತನಿಖೆ ಮಾಡಿ. ನಡವಳಿಕೆಗಳ ಬಗ್ಗೆ ನಂತರ ಕೇಳಿ)

Question 4: Did you receive a handout that was given to you at the group session? ಗುಂಪು ಕ್ಲಾಸ್ನಲ್ಲಿ ನೀವು ಭಾಗವಹಿಸಿದಾಗ ನೀಡಲಾದ ಆರೋಗ್ಯ ಮಾಹಿತಿ ಇದ್ದ ಚೀಟಿ ನೀವು ಪಡೆದುಕೊಂಡಿರ ?

[If yes]: Do you still have it? Why or why not? How have you used the handout since you've returned to the hospital? ಹೌದಾದರೆ, ಆ ಆರೋಗ್ಯ ಮಾಹಿತಿ ಇದ್ದ ಚೀಟಿ ಇನ್ನೂ ನಿಮ್ಮ ಹತ್ತಿರ ಇದೆಯಾ? ಏಕೆ ಅದನ್ನು ನಿಮ್ಮ ಹತ್ತಿರ ಇಟ್ಟುಕೊಳ್ಳಬೇಕು ಎಂದು ಅನಿಸಿತು? ಇಲ್ಲವಾದರೆ ಅವಶ್ಯಕತೆ ಇಲ್ಲ ಎಂದು ಏಕೆ ಅನಿಸಿತು? ನೀವು ಆಸ್ಪತ್ರೆಯಿಂದ ವಾಪಸ್ಸು ಬಂದಾಗಿನಿಂದ ಆ ಆರೋಗ್ಯ ಮಾಹಿತಿ ಇದ್ದ ಚೀಟಿಯನ್ನು ಹೇಗೆ ಬಳಸಿದ್ದೀರಿ?

Question 5: Have you used the whatsapp service mentioned in the class? Why or why not? ಗುಂಪು ಕ್ಲಾಸ್ನಲ್ಲಿ ತಿಳಿಸಿದ ವಾಟ್ಸಾಪ್ ಸೇವೆಯನ್ನು ನೀವು ಬಳಸಿದ್ದೀರಾ? ಬಳಸಿದ್ದರೆ ಏಕೆ ಬಳಸಿದಿರಿ? ಅಥವಾ ಇಲ್ಲವಾದರೆ ಏಕೆ ಬಳಸಲಿಲ್ಲ?

If yes, how have you used the whatsapp service? What are your thoughts about the whatsapp service? ಹೌದಾದರೆ, ನೀವು ವಾಟ್ಸಾಪ್ ಸೇವೆಯನ್ನು ಹೇಗೆ ಬಳಸಿದ್ದೀರಿ? ವಾಟ್ಸಾಪ್ ಸೇವೆಯ ಬಗ್ಗೆ ಏನು ಅನಿಸಿತು?

Question 6: Did they show videos as part of the class? ಅವರು ಕ್ಲಾಸ್ ನ ಭಾಗವಾಗಿ ವೀಡಿಯೊಗಳನ್ನು ತೋರಿಸಿದ್ರಾ?

[If yes] what do you remember from those videos? What did you think of those videos? [ಹೌದು ಅಂದ್ರೆ] ಆ ವೀಡಿಯೊಗಳಲ್ಲಿ ನಿಮಗೆ ಏನು ನೆನಪಿದೆ? ಆ ವೀಡಿಯೊಗಳ ಬಗ್ಗೆ ನಿಮ್ಮ ಅಭಿಪ್ರಾಯವೇನು?

Question 7: If you had a friend who just had a baby, would you recommend that she attend this group session? ಇತ್ತೀಚೆಗೆ ನಿಮ್ಮ ಆತ್ಮೀಯರಿಗೆ ಅಥವಾ ಪರಿಚಯದವರಿಗೆ ಯಾರಿಗಾದರೂ ಹೆರಿಗೆ ಆಗಿದ್ರೆ ಅವರಿಗೂ ಈ ಗುಂಪು ಕ್ಲಾಸ್ ನಲ್ಲಿ ಭಾಗವಹಿಸಲು ಹೇಳುತ್ತೀರಾ?

- [PROBE: Why or why not? ಯಾಕೆ ಭಾಗವಹಿಸಲು ಹೇಳುತ್ತೀರಾ ಅಥವಾ ಹೇಳುವುದಿಲ್ಲ?]

Question 8: In the group sessions, the Nurses talked about a lot of things that could go wrong for a baby. How did hearing about these things make you feel? Why? ಗುಂಪು ಕ್ಲಾಸ್ ನಲ್ಲಿ ನರ್ಸ್ ಮಗುವಿಗೆ ಉಂಟಾಗಬಹುದಾದ ಹಲವಾರು ತೊಂದರೆಗಳ ಬಗ್ಗೆ ಬಹಳಷ್ಟು ಮಾಹಿತಿ ನೀಡಿದರು. ಇದನ್ನು ಕೇಳಿ ನಿಮಗೆ ಏನನ್ನಿಸಿತು? ಮತ್ತು ಯಾಕೆ ಹಾಗನ್ನಿಸಿತು?

Question 9: Is there anything that you wanted to know about caring for [*baby name]* that you weren't taught in the group session? ಮಗುವನ್ನು ನೋಡಿಕೊಳ್ಳುವ ಬಗ್ಗೆ ಈ ಗುಂಪು ಕ್ಲಾಸ್ ನಲ್ಲಿ ಹೇಳಿಕೊಡದ ಬೇರೆ ಯಾವುದಾದರೂ ವಿಷಯದ ಬಗ್ಗೆ ತಿಳಿದುಕೊಳ್ಳಬೇಕಿತ್ತು ಅಂತ ನಿಮಗೆ ಅನ್ನಿಸ್ತಾ?

- [PROBES: What did you want to know? Why did you want to learn about this? ಏನು ತಿಳಿದುಕೊಳ್ಳಬೇಕು ಎಂದು ನಿಮಗೆ ಅನ್ನಿಸಿತು? ಇದರ ಬಗ್ಗೆ ಯಾಕೆ ತಿಳಿದುಕೊಳ್ಳಬೇಕು ಎನ್ನಿಸಿತು?]

Question 10: Have you had any other experience prior to the birth of [*baby name*] with other newborn babies? (*this includes any experience with previous children of their own or children of friends, family, or employers)*

ಈ ಮಗು ಹುಟ್ಟುವ ಮುಂಚೆ [ಮಗುವಿನ ಹೆಸರು] ನೀವು ಬೇರೆ ಎಳೆ ಮಕ್ಕಳನ್ನು ನೋಡಿಕೊಂಡಿರುವ ಅನುಭವವನ್ನು ಹೊಂದಿದ್ದೀರಾ? *(ಅಂದ್ರೆ, ಇದು ಅವರ ಹಿಂದಿನ ಮಕ್ಕಳು ಅಥವಾ ಸ್ನೇಹಿತರು ಮಕ್ಕಳು, ಕುಟುಂಬದ ಅಥವಾ ಉದ್ಯೋಗದಲ್ಲಿ ಯಾವುದಾದರು ಮಕ್ಕಳನ್ನು ನೋಡಿಕೊಡಿರುವ ಅನುಭವವನ್ನು ಒಳಗೊಂಡಿದೆ)*

· [PROBE: *ask for details on what kind of experience they have with newborn babies*
[PROBE:ಎಳೆಮಗುವಿನ ಆರೈಕೆಯಲ್ಲಿ *ಅವರು ಯಾವ ರೀತಿಯ ಅನುಭವವನ್ನು ಹೊಂದಿದ್ದಾರೆ ಎಂದು ವಿವರವಾಗಿ ಕೇಳಿ].*

Question 11: Have you attended any other information sessions that provide education on newborn health and maternal health?
ಎಳೆಮಗುವಿನ ಆರೋಗ್ಯ ಮತ್ತು ಬಾಣಂತಿಯ ಆರೋಗ್ಯದ ಬಗ್ಗೆ ಮಾಹಿತಿ ಕೊಡುವ ಬೇರೆ ಯಾವುದೇ ಅಧಿವೇಶನ ಅಥವಾ ಚರ್ಚೆಗಳಲ್ಲಿ ನೀವು ಭಾಗವಹಿಸಿದ್ದೀರಾ?

· [PROBES: When did you attend that other session? Where did you attend that other session? (*ask for specific facility*) What did you learn during the session?]
[PROBES:ಆ ಇತರ ಅಧಿವೇಶನದಲ್ಲಿ ಅಥವಾ ಚರ್ಚೆಗಳಲ್ಲಿ ನೀವು ಯಾವಾಗ ಭಾಗವಹಿಸಿದ್ದೀರಿ ? ಮತ್ತು ಎಲ್ಲಿ ಭಾಗವಹಿಸಿದ್ದೀರಿ? (ಯಾವ ಆರೋಗ್ಯ ಕೇಂದ್ರ, ಅಥವಾ ಯಾವ ಜಾಗದಲ್ಲಿ ಭಾಗವಯಿಸಿದ್ದರು ಎಂದು ನಿರ್ದಿಷ್ಟವಾಗಿ ಕೇಳಿ) ಅದರಲ್ಲಿ ನೀವು ಏನು ಕಲಿತಿದ್ದೀರಿ?]

Question 12: Before [*baby name*] was born, how did you feel about your ability to take care of your baby?ಈ ಮಗು ಹುಟ್ಟುವ ಮುಂಚೆ, ನಿಮ್ಮ ಮಗುವನ್ನು ನೋಡಿಕೊಳ್ಳುವ ನಿಮ್ಮ ಸಾಮರ್ಥ್ಯದ ಬಗ್ಗೆ ನಿಮಗೆ ಏನು ಅನ್ನಿಸಿತ್ತು?

- [PROBES: Were you worried or concerned? *If so, ask for details on what they w*orried about and why ನಿಮಗೆ ಏನಾದರೂ ಚಿಂತೆಯಾಗಿತ್ತಾ ಅಥವಾ ಅಥವಾ ಕಾಳಜಿಗಳು ಇದ್ದವಾ? ಹೌದಾದರೆ, ಯಾವ ವಿಷಯದ ಬಗ್ಗೆ ಚಿಂತೆ ಅಥವಾ ಅಥವಾ ಕಾಳಜಿಗಳು ಇತ್ತು ?ಯಾಕೆ ?]

If participant expresses worry/concerns ask Question 7. If no concerns, skip to Section II. ಭಾಗವಹಿಸುವವರು ಚಿಂತೆ / ಆತಂಕ ವ್ಯಕ್ತಪಡಿಸಿದರೆ ಪ್ರಶ್ನೆ 7ನ್ನು ಕೇಳಿ. ಇಲ್ಲವಾದರೇ, ಭಾಗ II ಕ್ಕೆ ಮುಂದುವರಿಯಿರಿ.<br><br>Question 13: Did the group sessions change your concerns ? ಈ ಕ್ಲಾಸ್ನಲ್ಲಿ ನೀವು ಭಾಗವಹಿಸಿದ್ರಿಂದ ನಿಮ್ಮ ಚಿಂತೆ ಅಥವಾ *ಆತಂಕ* ಬದಲಾಯಿತಾ?<br>● If Yes: What about the sessions caused your concerns to change? (probe if concerns increased or decreased) ಈ ಗುಂಪು ಕ್ಲಾಸ್ ನಲ್ಲಿ ಹೇಳಿಕೊಟ್ಟ ಯಾವ ವಿಷಯ ನಿಮ್ಮ ಚಿಂತೆ /*ಆತಂಕ*ಗಳನ್ನು ಬದಲಾಯಿಸಿತು? (ಚಿಂತೆ ಅಥವಾ *ಆತಂಕ* ಕಡಿಮೆಯಾದವಾ ಅಥವಾ ಹೆಚ್ಚಾದವಾ ಎಂದು ಕೇಳಿ)<br>● If No: Since [*baby name*] was born, has anything (else) caused your concerns to change? Why? ಇಲ್ಲವಾದರೆ: ಮಗು ಹುಟ್ಟಿದ ಮೇಲೆ ನಿಮ್ಮ ಆತಂಕಗಳು ಬದಲಾಗಲು ಬೇರೆ ಯಾವುದಾದರೂ ಕಾರಣಗಳಿತ್ತಾ? ಯಾಕೆ?

SECTION II ಭಾಗ II

I'd like to hear about what happened when you went home from the hospital with [*baby name*]. I'm going to ask you about different things that you may or may not have done for [*baby name*].ಮಗು ಜೊತೆ ಆಸ್ಪತ್ರೆಯಿಂದ ಮನೆಗೆ ಹೋದ ಮೇಲೆ ನಡೆದ ವಿಷಯಗಳ ಬಗ್ಗೆ ಕೆಲವು ಪ್ರಶ್ನೆಗಳನ್ನು ಕೇಳುತ್ತೇನೆ. ನೀವು ಮಗುವಿಗೆ ಮಾಡಿರಬಹುದಾದ ಅಥವಾ ಮಾಡದಿರುವ ವಿಷಯಗಳ ಬಗ್ಗೆ ನಿಮ್ಮನ್ನು ಕೇಳುತ್ತೇನೆ.
Question 14: First I want to ask you about <u>feeding</u> [*baby name*] . Sometimes people feed their babies other things other than breastmilk such as cow's milk and ragi [*interviewer can also use other examples listed below*] We're interested in better understanding when and why people give these things to their babies. I'm going to read you a list of things that some people feed their babies and I'd like you to tell me if you ever feed your baby any of these, even if you have only given it to your baby one time.<br>ಮೊದಲಿಗೆ ಮಗುವಿಗೆ ಏನೇನು ಕೊಡುತ್ತೀರಾ ಅಂತ ಕೆಲವು ಪ್ರಶ್ನೆಗಳನ್ನು ಕೇಳ್ತೀನಿ . ಕೆಲವೊಮ್ಮೆ ಜನರು ತಮ್ಮ ಎಳೆಮಗುವಿಗೆ ಎದೆಹಾಲು ಹೊರತುಪಡಿಸಿ ಹಸುವಿನ ಹಾಲು ಮತ್ತು ರಾಗಿ ಮುಂತಾದ ಆಹಾರವನ್ನು

ಕೊಡುತ್ತಾರೆ [ಸಂದರ್ಶಕರು ಕೆಳಗೆ ಪಟ್ಟಿ ಮಾಡಲಾದ ಇತರ ಆಹಾರಗಳ ಉದಾಹರಣೆಗಳನ್ನು ಸಹ ಹೇಳಬಹುದು] ಜನರು ಹೀಗೆ ತಮ್ಮ ಎಳೆಮಗುವಿಗೆ ಯಾವಾಗ ಮತ್ತು ಏಕೆ ಈ ವಸ್ತುಗಳನ್ನು ಕೊಡುತ್ತಾರೆ ಎಂಬುದನ್ನು ಚೆನ್ನಾಗಿ ಅರ್ಥಮಾಡಿಕೊಳ್ಳಲು ನಾವು ಆಸಕ್ತಿ ಹೊಂದಿದ್ದೇವೆ. ಕೆಲವು ಜನರು ತಮ್ಮ ಎಳೆಮಗುವಿಗೆ ಆಹಾರವನ್ನು ನೀಡುವ ವಸ್ತುಗಳ ಪಟ್ಟಿಯನ್ನು ನಾನು ನಿಮಗೆ ಓದುತ್ತೇನೆ. ನೀವು ನಿಮ್ಮ ಮಗುವಿಗೆ ಇವುಗಳಲ್ಲಿ ಯಾವುದಾದರೂ ಆಹಾರವನ್ನು ಕೊಟ್ಟಿದ್ದೀರಾ ಎಂದು ನನಗೆ ತಿಳಿಸಿ, ನೀವು ಅದನ್ನು ನಿಮ್ಮ ಮಗುವಿಗೆ ಒಂದು ಬಾರಿ ಮಾತ್ರ ಕೊಟ್ಟಿದ್ದರೂ ಸಹ ನನಗೆ ಹೇಳಿ

Question 15: Please tell me if you have ever fed your baby any of the following: *For each item mentioned, ask for details on why, when, how frequently they feed their baby X?*
ನಿಮ್ಮ ಮಗುವಿಗೆ ನೀವು ಈ ಕೆಳಗಿನ ಯಾವುದನ್ನಾದರೂ ಕೊಟ್ಟಿದ್ದೀರಾ ಎಂದು ದಯವಿಟ್ಟು ನನಗೆ ತಿಳಿಸಿ: ಮೇಲೆ ತಿಳಿಸಲಾದ ವಸ್ತುಗಳನ್ನು, ಅವರು ತಮ್ಮ ಮಗುವಿಗೆ ಯಾವಾಗ, ಏಕೆ ಮತ್ತು ಎಷ್ಟು ಬಾರಿ ಕೊಟ್ಟಿದ್ದಾರೆ ಎಂಬುದರ ವಿವರಗಳನ್ನು ಕೇಳಿ

- Jaggery Water ಬೆಲ್ಲದ ನೀರು
- Cow's milk ಹಸು ಹಾಲು
- Pasteurized milk ಪಾಶ್ಚರೀಕರಿಸಿದ ಹಾಲು
- Honey ಜೇನುತುಪ್ಪ
- Ragi ರಾಗಿ
- Gripe Water, ಗ್ರಾಯ್ಪ್ ವಾಟರ್
- Nutmeg paste ಜಾಯಿಕಾಯಿ ಪೇಸ್ಟ್
- Castor oil ಹರಳೆಣ್ಣೆ
- Any other animal milk ಇತರ ಪ್ರಾಣಿಗಳ ಹಾಲು

Question 16: Do you ever breastfeed [*baby name*]? ಮಗುವಿಗೆ ನೀವು ಎದೆಹಾಲು ಕುಡಿಸಿದ್ದೀರಾ?

- If YES: how many times in a day? Do you find it easy/ difficult? Why? ದಿನಕ್ಕೆ ಎಷ್ಟು ಸಲ ಕುಡಿಸಿದ್ದೀರಾ? ನಿಮಗೆ ಇದು ಸುಲಭ / ಕಷ್ಟ ಅನ್ನಿಸತ್ತಾ? ಯಾಕೆ?
- If difficult: is there anything you tried to help with the problem? Where did you learn or hear about doing that? ಕಷ್ಟವಾದರೆ:ಈ ಸಮಸ್ಯೆ ಪರಿಹರಿಸಲು ನೀವೇನಾದರೂ ಮಾಡಿದಿರಾ? ? ಹೀಗೆ ಮಾಡುವುದನ್ನು ಎಲ್ಲಿಂದ ಕಲಿತಿರಿ ಅಥವಾ ಕೇಳಿದಿರಿ?

- If NO: There are many reasons why women do not breastfeed. Please tell me about why you do not breastfeed.ಎದೆ ಹಾಲು ಕುಡಿಸುತ್ತಿಲ್ಲವಾದರೆ: ಹಾಲು ಕುಡಿಸದಿರಲು ಬೇರೆ ಬೇರೆ ಕಾರಣಗಳಿರುತ್ತದೆ. ನೀವು ಯಾಕೆ ಹಾಲು ಕುಡಿಸುವುದಿಲ್ಲ ಅಂತ ತಿಳಿಸಿ.

If participant does not breastfeeds, move to Question 11:
ಭಾಗವಹಿಸುವವರು ಏನನ್ನು ಕೊಟ್ಟಿಲ್ಲ ಅಂತ ಹೇಳಿದ್ರೆ, ಪ್ರಶ್ನೆ 11 ಕ್ಕೆ ಹೋಗಿ.

Question 17: How confident do you feel in your ability to breastfeed [*baby name*]? ಮಗುವಿಗೆ ಬೇಕಾದಷ್ಟು ಹಾಲು ಕುಡಿಸ್ತೀನಿ ಅಂತ ನಿಮಗೆ ನಿಮ್ಮ ಬಗ್ಗೆ ನಂಬಿಕೆ ಇದೆಯೇ?

- [PROBES: when you breastfeed, Did group session help with confidence? What helped? If no: did session cause you to feel less confident? Please explain ವಿಶ್ವಾಸವಿದ್ದರೆ :ಮಗುವಿಗೆ ಹಾಲು ಕುಡಿಸುವ ವಿಚಾರದಲ್ಲಿ ನಿಮ್ಮ ಬಗ್ಗೆ ನೀವು ವಿಶ್ವಾಸವನ್ನು ಹೊಂದಲು ಈ ಕ್ಲಾಸ್ ಎಷ್ಟರ ಮಟ್ಟಿಗೆ ಸಹಾಯ ಮಾಡಿದೆ? ಯಾವ ವಿಷಯ ಸಹಾಯವಾಯಿತು? ಇಲ್ಲವಾದರೆ: ಈ ಕ್ಲಾಸ್ ನಿಂದ ನಿಮ್ಮ ವಿಶ್ವಾಸದ ಮಟ್ಟ ಕಡಿಮೆಯಾಯಿತು ಅಂತ ಅನ್ನಿಸುತ್ತದೆಯಾ? ದಯವಿಟ್ಟು ವಿವರಿಸಿ.

Question 18: What do you remember about breastfeeding from the group session?
ಎದೆಹಾಲು ಕುಡಿಸುವುದರ ಬಗ್ಗೆ ಗುಂಪು ಕ್ಲಾಸ್ ನಲ್ಲಿ ಹೇಳಿಕೊಟ್ಟ ಯಾವ ಅಂಶಗಳು ನಿಮಗೆ ನೆನಪಿದೆ?

Question 19: How, if at all, do you think the group session affected the way you feed [*baby name*] at home? ಈ ತರಬೇತಿಯಿಂದ ಮಗುವಿಗೆ ನೀವು ಹಾಲು ಕುಡಿಸುವ ವಿಧಾನದಲ್ಲಿ ಏನಾದರೂ ಪರಿಣಾಮ ಬೀರಿದೆ ಎಂದು ನಿಮಗೆ ಅನಿಸುತ್ತದೆಯಾ?

The next few questions will ask you about handwashing, particularly when taking care of [*baby name*]. ಮುಂದಿನ ಕೆಲವು ಪ್ರಶ್ನೆಗಳು ಕೈತೊಳೆಯುವುದರ ಬಗ್ಗೆ ಅದರಲ್ಲೂ ಮಗುವನ್ನು ನೋಡಿಕೊಳ್ಳುವ ಸಮಯದಲ್ಲಿ ಕೈತೊಳೆಯುವುದರ ಬಗ್ಗೆ ಕೇಳುತ್ತೇವೆ.

Question 20: Can you describe how you wash your hands? ನೀವು ನಿಮ್ಮ ಕೈಯನ್ನು ಹೇಗೆ ತೊಳೆದುಕೊಳ್ಳುತ್ತೀರಿ ಎಂದು ಸ್ವಲ್ಪ ವಿವರಿಸಿ.

- [PROBES: Ask if they have soap at home/how often do you have soap at home. Access to clean water? How often?ನಿಮ್ಮ ಮನೆಯಲ್ಲಿ ಸೋಪ್ ಇದೆಯಾ? / ನಿಮ್ಮ ಮನೆಯಲ್ಲಿ ಯಾವಾಗ ಯಾವಾಗ ಸೋಪ್ ಇರುತ್ತದೆ ?ಶುದ್ಧ ನೀರು ನಿಮ್ಮ ಮನೆಗೆ ಬರುತ್ತಾ? ಎಷ್ಟು ದಿನಕ್ಕೊಮ್ಮೆ ನೀರು ಬರುತ್ತದೆ?]

Question 21: Do you think hand washing is important? Why/why not? ಕೈತೊಳೆದುಕೊಳ್ಳುವುದು ಮುಖ್ಯ ಅಂತ ನಿಮಗೆ ಅನ್ನುಸ್ತಾ ? ಯಾಕೆ ಮುಖ್ಯ ಅಂತ ಅನಿಸುತ್ತೆ | ಯಾಕೆ ಅನ್ನಿಸುವುದಿಲ್ಲ?

Question 22: Is there anything that makes handwashing hard for you? ಕೈತೊಳೆದುಕೊಳ್ಳುವುದಕ್ಕೆ ಏನಾದರೂ ತೊಂದರೆ ಇದೆಯಾ?

- If YES: What makes it hard? ತೊಂದರೆ ಇದ್ದರೆ :ಕೈತೊಳೆದುಕೊಳ್ಳುವುದಕ್ಕೆ ಏನು ತೊಂದರೆ ಇದೆ? * PROBE for times/events that may be difficult for handwashing. Some examples you can provide: before feeding baby? After changing diapers? ಕೈತೊಳೆದುಕೊಳ್ಳುವುದಕ್ಕೆ ಯಾವಾಗ ಕಷ್ಟ ಆಗುತ್ತೆ? ನೀವು ಈ ಉದಾಹರಣೆಗಳನ್ನು ಕೊಡಬಹುದು: ಮಗುವಿಗೆ ಹಾಲು ಕುಡಿಸುವ ಮೊದಲು? ಡಯಾಪರ್ ಬದಲಾಯಿಸಿದ ನಂತರ?]

Question 23: Did you wash your hands today? ನೀವು ಇವತ್ತು ನಿಮ್ಮ ಕೈತೊಳೆದುಕೊಂಡಿದ್ದಿರಾ?

- If YES: When? Why? Probe on the situation, any other times you washed your hands today? ಹೌದಾದರೆ: ಯಾವಾಗ? ಯಾಕೆ? ಸನ್ನಿವೇಶದ ಬಗ್ಗೆ ಕೇಳಿ, ಇವತ್ತು ಇನ್ನ್ಯಾವುದಾದರೂ ಸಂದರ್ಭದಲ್ಲಿ ಕೈ ತೊಳೆದುಕೊಂಡಿದ್ದಿರಾ?

Question 24: What do you remember about handwashing from the group session? ಕೈತೊಳೆಯುವುದರ ಬಗ್ಗೆ ಗುಂಪು ಕ್ಲಾಸ್ ನಲ್ಲಿ ಹೇಳಿಕೊಟ್ಟ ಯಾವ ಅಂಶಗಳು ನಿಮಗೆ ನೆನಪಿದೆ?

Question 25: Do you think the group session has affected your hand washing at home?ಕ್ಲಾಸ್ ನಂತರ ಮನೆಯಲ್ಲಿ ನೀವು ಕೈತೊಳೆಯುವ ವಿಧಾನದಲ್ಲಿ ಈ ಗುಂಪು ಕ್ಲಾಸ್ ಯಾವ ರೀತಿಯ ಪರಿಣಾಮ ಮಾಡಿದೆ?

- If YES: How so? ಹೌದಾದರೆ: ಹೇಗೆ?

Next, I'm going to ask you about ________ [*baby name*]'s umbilical cord. ಈಗ ನಾನು ಮಗುವಿನ ಹೊಕ್ಕಳಬಳ್ಳಿಯ ಬಗ್ಗೆ ಪ್ರಶ್ನೆಗಳನ್ನು ಕೇಳುತ್ತೇನೆ.
Question 26: Tell me about how you have cared for [*baby name*]'s umbilical cord area. ಮಗುವಿನ ಹೊಕ್ಕಳಬಳ್ಳಿಯನ್ನು ಹೇಗೆ ಆರೈಕೆಯನ್ನು ಮಾಡಿದ್ರಿ ಅಂತ ತಿಳಿಸಿ<br><br>● [PROBES: *Have them walk through what they did before the cord fell off.* Did you put anything on the cord? If so, what? and why? *Probe around coconut oil, powder, medicated cream, talcum powder, spices.* ಹೊಕ್ಕಳಬಳ್ಳಿ ಬೀಳುವ ಮೊದಲು ಏನೇನು ಮಾಡಿದ್ರು ಅಂತ ತಿಳಿದುಕೊಳ್ಳಿ. ನೀವು ಹೊಕ್ಕಳಬಳ್ಳಿಯ ಮೇಲೆ ಏನಾದರೂ ಹಾಕಿದ್ದಿರಾ? ಹೌದಾದರೆ, ಏನು? ಮತ್ತು ಯಾಕೆ? ಕೊಬ್ಬರಿ ಎಣ್ಣೆ ಪೌಡರ್, ಆಯಿಂಟ್ ಮೆಂಟ್, ಟಾಲ್ಕಮ್ ಪೌಡರ್, ಮಸಾಲೆಗಳು ಏನಾದರೂ ಹಾಕಿದ್ದರಾ ಕೇಳಿ]<br><br>Question 27: How confident did you feel about taking care of [baby name] umbilical cord? ಮಗುವಿನ ಹೊಕ್ಕಳಬಳ್ಳಿಯ ಆರೈಕೆಯ ಬಗ್ಗೆ ನಿಮಗೆ ಎಷ್ಟರ ಮಟ್ಟಿಗೆ ವಿಶ್ವಾಸ ಇದೆ?<br>● [PROBE: Why? ಯಾಕೆ?]<br><br>Question 28: What do you remember about dry cord care from the group session? ಹೊಕ್ಕಳಬಳ್ಳಿಯನ್ನು ಒಣಗಿರುವಂತೆ ಇಟ್ಟುಕೊಳ್ಳುವ ವಿಧಾನದ ಬಗ್ಗೆ ಗುಂಪು   ಕ್ಲಾಸ್ ನಲ್ಲಿ ಹೇಳಿದ ಯಾವ ವಿಷಯ ನಿಮಗೆ ನೆನಪಿದೆ?<br>● If mother does not remember anything about dry cord care, provide a definition: Do not put anything on cord or cord area; keep the area dry ತಾಯಿಗೆ ನೆನಪಿಲ್ಲದಿದ್ದರೆ , ವಿವರಣೆ ನೀಡಿ: ಹೊಕ್ಕಳಬಳ್ಳಿಯ ಮೇಲೆ ಅಥವಾ ಹೊಕ್ಕಳಬಳ್ಳಿಯ ಸುತ್ತಲೂ ಏನನ್ನೂ ಹಾಕಬಾರದು; ಇದರ ಸುತ್ತ ಒಣ ಇರಿಸಬೇಕು.<br><br>Question 29: How did the group session affect how you cared for [*baby name]* cord at home? ಮನೆಯಲ್ಲಿ ಹೊಕ್ಕಳ ಬಳ್ಳಿಯನ್ನು ನೀವು ಹೇಗೆ ನೋಡಿಕೊಂಡಿದ್ದೀರಿ ಎನ್ನುವುದರ ಬಗ್ಗೆ ಕ್ಲಾಸ್ ಹೇಗೆ ಪರಿಣಾಮ ಬೀರಿತು?

Now I want to ask you about skin to skin contact with [baby name].ಈಗ ನಾನು ಮಗುವಿಗೆ ಮಾಡುವ ಚರ್ಮದಿಂದ ಚರ್ಮದ ಆರೈಕೆಯ ಬಗ್ಗೆ ಕೆಲವು ಪ್ರಶ್ನೆಗಳನ್ನು ಕೇಳುತ್ತೇನೆ.

Question 30: What do you remember about skin to skin contact from the group session? ಚರ್ಮದಿಂದ ಚರ್ಮದ ಆರೈಕೆಯ ಬಗ್ಗೆ ಗುಂಪು ಕ್ಲಾಸ್ ನಲ್ಲಿ ಹೇಳಿಕೊಟ್ಟಿರುವ ಯಾವ ವಿಷಯ ನಿಮಗೆ ನೆನಪಿದೆ?

- If mother does not remember anything ask Question 24 ತಾಯಿ ಏನೂ ನೆನಪಿಲ್ಲದಿದ್ದರೆ, ಪ್ರಶ್ನೆ 24 ಕ್ಕೆ ಹೋಗಿ
- If mother remembers correctly skip to question 25 ತಾಯಿ ಸರಿಯಾಗಿ ನೆನಪಿಟ್ಟುಕೊಂಡಿದ್ದರೆ, ಪ್ರಶ್ನೆ 25 ಕ್ಕೆ ಹೋಗಿ

Question 31: Can you tell me what you think skin-to-skin contact is? ಚರ್ಮದಿಂದ ಚರ್ಮದ ಆರೈಕೆ ಅಂದರೆ ಏನು ಎಂದು ಹೇಳುತ್ತೀರಾ?

- If incorrect, provide definition: putting the bare baby on the bare chest of the mother to provide warmth ತಪ್ಪಾಗಿದ್ದರೆ, ವಿವರಣೆ ನೀಡಿ: ಮಗುವಿನ ಬರೀ ಮೈಯನ್ನು ತಾಯಿಯ ಬರೀ ಎದೆ ಮೇಲೆ ಹಾಕಿಕೊಂಡು ಬೆಚ್ಚಗೆ ಮಾಡುವ ವಿಧಾನ.

Question 32: Given how you (or I) have described skin-to-skin contact, do you do this with [*baby name*]?ನೀವು (ಅಥವಾ ನಾನು) ಚರ್ಮದಿಂದ ಚರ್ಮದ ಆರೈಕೆಯ ಬಗ್ಗೆ ನೀಡಿರುವ ವಿವರಣೆಯಂತೆ, ಆರೈಕೆ ಮಾಡುತ್ತೀರಾ?

- If YES: How long do you usually keep [*baby name*] on your chest? How often do you do this?ಹೌದಾದರೆ: ಮಗುವನ್ನು ನಿಮ್ಮ ಎದೆಯ ಮೇಲೆ ಎಷ್ಟು ಹೊತ್ತು ಇಟ್ಟುಕೊಂಡಿರುತ್ತೀರಾ? ದಿನಕ್ಕೆ ಈ ರೀತಿ ಎಷ್ಟು ಸಲ ಮಾಡುತ್ತೀರಾ?
- If NO : Have you ever had skin to skin contact with [*baby name*]? ಇಲ್ಲವಾದರೆ: ನೀವು ಯಾವಾಗಲಾದರೂ ಮಗುವಿನ ಜೊತೆ ಚರ್ಮದಿಂದ ಚರ್ಮದ ಆರೈಕೆ ಮಾಡಿದ್ದಿರಾ?
    - If yes, ಹೌದಾದರೆ:
        - Why did you stop?ಯಾಕೆ ನಿಲ್ಲಿಸಿದಿರಿ?

- When you did skin to skin, can you describe to me how you had skin-to-skin with [baby's name]? For how long/frequently? ಮುಂಚೆ ಚರ್ಮದಿಂದ ಚರ್ಮದ ಆರೈಕೆ ಮಾಡುತ್ತಿದ್ದಾಗ ನೀವು ಯಾವ ರೀತಿ ಮಾಡಿದ್ದೀರಿ ಎಂದು ವಿವರಿಸುತ್ತೀರಾ? ಎಷ್ಟು ಹೊತ್ತು/ ಎಷ್ಟು ಗಂಟೆಗೊಂದು ಸಲ ಹೀಗೆ ಮಾಡಿದ್ದೀರಿ?

Question 33 What is your understanding of the benefit about skin to skin contact with [baby name]?ಮಗುವಿನೊಂದಿಗೆ ಚರ್ಮದಿಂದ ಚರ್ಮದ ಆರೈಕೆ ಮಾಡುವುದರ ಉಪಯೋಗಗಳ ಬಗ್ಗೆ ನೀವು ಏನು ಅರ್ಥ ಮಾಡಿಕೊಂಡಿದ್ದೀರಿ?

Question 34: Did you ever feel unsure how or when to do skin-to-skin? Did group session help? ಚರ್ಮದಿಂದ ಚರ್ಮದ ಆರೈಕೆ ಮಾಡುವುದು ಹೇಗೆ ಅಥವಾ ಯಾವಾಗ ಮಾಡಬೇಕು ಎನ್ನುವ ಬಗ್ಗೆ ಖಚಿತವಾಗಿಲ್ಲ ಅನ್ಸುತ್ತಾ ? ಈ ಗುಂಪು ಕ್ಲಾಸ್‌ನಲ್ಲಿ ಖಚಿತವಾಯಿತಾ/ ಪರಿಹಾರವಾಯಿತಾ?]

I'm now going to ask you some questions about your diet since you've come home from the hospital. ನೀವು ಹಾಸ್ಪಿಟಲ್ ನಿಂದ ಮನೆಗೆ ಬಂದ ಮೇಲೆ ನೀವು ಏನೇನು ತಿನ್ನುತ್ತಿದ್ದೀರಿ ಎನ್ನುವ ಬಗ್ಗೆ ಕೆಲವು ಪ್ರಶ್ನೆಗಳನ್ನು ಕೇಳುತ್ತೇನೆ.

Question 35: Do you remember what was said in the group session about recommendations for what to eat and drink after having a baby? ಮಗು ಹುಟ್ಟಿದ ಮೇಲೆ ಏನೇನು ತಿನ್ನಬೇಕು ಮತ್ತು ಕುಡಿಯಬೇಕು ಎನ್ನುವ ಬಗ್ಗೆ ಗುಂಪು ಕ್ಲಾಸ್ ನಲ್ಲಿ ಹೇಳಿದ ವಿಷಯಗಳು ನಿಮಗೆ ನೆನಪಿದೆಯಾ?

- [PROBE: What do you remember?ನಿಮಗೆ ಏನೇನು ನೆನಪಿದೆ?]

Question 36: What do you typically eat and drink since giving birth to [*baby name*]? ಮಗು ಹುಟ್ಟಿದ ಮೇಲೆ ನೀವು ಸಾಮಾನ್ಯವಾಗಿ ಏನು ತಿನ್ನುತ್ತೀರಿ ಮತ್ತು ಕುಡಿಯುತ್ತೀರಿ?

- [PROBES: Why? Anything else? ಯಾಕೆ? ಇನ್ನೇನು ತಿನ್ನುತ್ತೀರಿ? ಏನು ಕುಡಿಯುತ್ತೀರಿ?]

Question 37: Are there any foods or liquids that you haven't eaten because you thought it would be bad for the baby? ಮಗುವಿಗೆ ಒಳ್ಳೆಯದಲ್ಲ ಎಂಬ ಕಾರಣಕ್ಕೆ ನೀವು ಯಾವುದಾದರೂ ಆಹಾರ ತಿನ್ನುವುದನ್ನು ಅಥವಾ ಪಾನೀಯವನ್ನು ಕುಡಿಯುವುದನ್ನು ಬಿಟ್ಟಿದ್ದೀರ?

- [PROBES: For example, fruits, vegetables, grains, etc. Why? Anything else?; ask for details on where they got that information from- the group session?, *advice/ recommendation from family or community?*ಉದಾಹರಣೆಗೆ, ಯಾವುದಾದರೂ ಹಣ್ಣುಗಳು, ತರಕಾರಿಗಳು, ಧಾನ್ಯಗಳು ಇತ್ಯಾದಿಗಳನ್ನು ಬಿಟ್ಟಿದ್ದೀರ? ಯಾಕೆ? ಇನ್ನೇನಾದರೂ ತಿನ್ನುವುದಿಲ್ಲವಾ/ ಕುಡಿಯುವುದಿಲ್ಲವಾ? ಈ ಮಾಹಿತಿ ಅವರಿಗೆ ಎಲ್ಲಿ ಸಿಕ್ಕಿತು ತಿಳಿಸಿ? ಗುಂಪು ಕ್ಲಾಸ್ ನಲ್ಲಾ? ಮನೆಯವರಿಂದ? ಅಥವಾ ಅಕ್ಕಪಕ್ಕದವರು ಅಥವಾ ಬೇರೆಯವರು ಹೇಳಿದ್ರಾ?]

I'm now going to ask you some questions about burping your baby
ನಿಮ್ಮ ಮಗುವಿಗೆ ಎದೆ ಹಾಲು ಕುಡಿಸಿ ತೇಗಿಸುವ ಬಗ್ಗೆ ನಾನು ಈಗ ಕೆಲವು ಪ್ರಶ್ನೆಗಳನ್ನು ಕೇಳುತ್ತೇನೆ.

Question 38: Do you burp [*baby name*]?
ಪ್ರಶ್ನೆ 35: ನೀವು [ಮಗುವಿನ ಹೆಸರನ್ನು] ಮಗುವನ್ನು ಎದೆ ಹಾಲು ಕುದಿಸಿದ ನಂತ್ರ ತೇಗಿಸ್ತಿರ?

- If Yes: When do you typically burp [*baby name*]?
  ಹೌದು ಅಂದ್ರೆ : ನೀವು ಸಾಮಾನ್ಯವಾಗಿ [ಮಗುವಿನ ಹೆಸರು] ಯಾವಾಗ ತೇಗಿಸ್ತಿರಿ?
- If No: Why do you not burp [*baby name*]?
  ಇಲ್ಲ ಅಂದ್ರೆ : ನೀವು [ಮಗುವಿನ ಹೆಸರನ್ನು] ಏಕೆ ತೇಗಿಸಲ್ಲ?

Question 39: What is your understanding of the benefits of burping [*baby name*]?
ಪ್ರಶ್ನೆ 36: [ಮಗುವಿನ ಹೆಸರು] ತೇಗಿಸುವುದರ ಉಪಯೋಗಗಳ ಬಗ್ಗೆ ನೀವು ಏನು ಅರ್ಥ ಮಾಡಿಕೊಂಡಿದ್ದೀರಿ?

Question 40: How confident did you feel about burping [*baby name*]?
[ಮಗುವಿನ ಹೆಸರು] ತೇಗಿಸುವದರ ಬಗ್ಗೆ ನಿಮಗೆ ಎಷ್ಟು ವಿಶ್ವಾಸವಿದೆ ಅಂತ ನಿಮಗೆ ಅನ್ಸುತ್ತೆ?

- [PROBE: Why?]
- [PROBE: ಯಾಕೆ ?]

Question 41: Do you remember what was said in the group session about burping?
ಪ್ರಶ್ನೆ 38: ತೇಗಿಸುವದರ ಬಗ್ಗೆ ಗುಂಪು ಕ್ಲಾಸ್ ನಲ್ಲಿ ಏನು ಹೇಳಿದ್ರು ಅಂತ ನಿಮಗೆ ನೆನಪಿದ್ದೀಯಾ?

- [PROBE: What do you remember?]
- [PROBE: ನಿಮಗೆ ಏನು ನೆನಪಿದೆ?]

SECTION III: For baby with reported complications *only*
ಭಾಗ III: ದಾಖಲಿತ ಸಮಸ್ಯೆಗಳಿರುವ ಮಕ್ಕಳಿಗಾಗಿ ಮಾತ್ರ

Question 42: In the survey, you said that [*baby name*] had a problem with [*type of complication*]. I'm going to ask you some questions about your experience. Please explain to me what happened. ಫೋನ್ ಸಮೀಕ್ಷೆಯಲ್ಲಿ ನೀವು ಮಗುವಿಗೆ ಸಮಸ್ಯೆ ಇತ್ತು ಎಂದು ಹೇಳಿದಿರಿ. ಈಗ ನಾನು ಈ ಸಮಸ್ಯೆ ಬಗ್ಗೆ ನಿಮ್ಮ ಅನುಭವವನ್ನು ಕೇಳುತ್ತೇನೆ. ಏನಾಗಿತ್ತು ಎಂದು ನನಗೆ ದಯವಿಟ್ಟು ವಿವರಿಸಿ.

- [PROBES: How/when did you realize there was a problem with [baby name]?ಮಗುವಿಗೆ ಸಮಸ್ಯೆಯಾಗಿದೆ ಎಂದು ಯಾವಾಗ ನಿಮಗೆ ಗೊತ್ತಾಯಿತು? ಹೇಗೆ ಗೊತ್ತಾಯಿತು?]

Question 43: What did you do after you realized [baby name] had this problem?ಮಗುವಿಗೆ ಈ ಸಮಸ್ಯೆ ಇದೆ ಎಂದು ಗೊತ್ತಾದಾಗ ನೀವು ಏನು ಮಾಡಿದಿರಿ?

- [PROBES: Did you go anywhere for help? Where did you go? Did someone come to your home, and if so, who? Was it easy or hard to get help?ನೀವು ಸಹಾಯಕ್ಕಾಗಿ ಎಲ್ಲಿಯಾದರೂ ಹೋಗಿದ್ದಿರಾ? ಎಲ್ಲಿ ಹೋಗಿದ್ದಿರಿ? ಯಾರಾದರೂ ನಿಮ್ಮ ಮನೆಗೆ ಬಂದಿದ್ದರಾ? ಹೌದಾದರೆ ಯಾರು ಬಂದಿದ್ದರು? ಸಹಾಯ ಸಿಗುವುದು ಸುಲಭವಾಗಿತ್ತಾ ಅಥವಾ ಕಷ್ಟವಾಗಿತ್ತಾ?]

Question 44: Did you learn anything in the group session that helped you know [*baby name*] had this problem? ಮಗುವಿಗೆ ಈ ಸಮಸ್ಯೆ ಇದೆ ಎಂದು ನೀವು ತಿಳಿದುಕೊಳ್ಳಲು ಗುಂಪು ಕ್ಲಾಸ್ ನಲ್ಲಿ ಕಲಿತಿದ್ದ ಯಾವುದಾದರೂ ವಿಷಯ ನಿಮಗೆ ಸಹಾಯ ಆಯ್ತಾ?

- If YES: What? *Probe for details around recognizing symptoms, danger signs etc.* ಹೌದು ಎಂದರೆ ಯಾವ ವಿಷಯದಿಂದ ಸಮಸ್ಯೆಯನ್ನು ಗುರ್ತಿಸಲು ಸಹಾಯ ಆಯ್ತು ? ಲಕ್ಷಣಗಳನ್ನು ಗುರುತಿಸುವುದು, ಅಪಾಯದ ಸೂಚನೆಗಳು ಗುರುತಿಸುವುದು -ವಿವರಗಳನ್ನು ಕೇಳಿ

Question 45: Did you learn anything in the group session that helped you know what to do to help with the problem? ಈ ಸಮಸ್ಯೆಗೆ ಏನು ಮಾಡಬೇಕು ಅಂತ ಗುಂಪು ಕ್ಲಾಸ್ನಲ್ಲಿ ಕಲಿತಿದ್ದು ಏನಾದರೂ ಸಹಾಯ ಆಯ್ತಾ?

- If YES: What? Was doing this difficult or easy? Why?, Did you call anyone for help or go to the clinic/hospital? Ask for details if mother called a doctor, healer, family member etc. and why.ಹೌದಾದರೆ : ಏನು ಕಲಿತಿದ್ದಿರಿ? ಇದನ್ನು ಮಾಡುವುದು ಸುಲಭವಾಗಿತ್ತಾ ಅಥವಾ ಕಷ್ಟವಾಗಿತ್ತಾ? ಯಾಕೆ? ನೀವು ಯಾರನ್ನಾದರೂ ಸಹಾಯಕ್ಕಾಗಿ ಕರೆದಿರಾ ಅಥವಾ ಕ್ಲಿನಿಕ್ / ಹಾಸ್ಪಿಟಲ್ ಗೆ ಹೋಗಿದ್ದಿರಾ?ಡಾಕ್ಟರ್, ಔಷಧಿ ಕೊಡುವವರು, ಕುಟುಂಬದ ಸದಸ್ಯರು ಇತ್ಯಾದಿ ಯಾರನ್ನು ಕರೆದರು ಎಂಬ ವಿವರಗಳನ್ನು ಕೇಳಿ.
- If NO: Where did you get your information for how to help [*baby name*] with this problem? ಇಲ್ಲವಾದರೆ: ಈ ಮಗುವನ್ನು ಹೇಗೆ ನೋಡಿಕೊಳ್ಳಬೇಕು ಎನ್ನುವ ಮಾಹಿತಿ ನಿಮಗೆ ಎಲ್ಲಿ ಸಿಕ್ಕಿತು?

Question 46: Did any of your family members think you should have handled the problem in a different way?

ಪ್ರಶ್ನೆ 46: ನಿಮ್ಮ ಮನೆಯವರು ಯಾರಾದರೂ ನೀವು ಬೇರೆ ರೀತಿಯಲ್ಲಿ ಈ ಸಮಸ್ಯೆಯನ್ನು ನಿಭಾಯಿಸಬೇಕಿತ್ತು ಎಂದು ಭಾವಿಸಿದರಾ?

- [PROBES: what did they advise? Did you take their advice? Why or why not?ಅವರು ಏನೆಂದು ಸಲಹೆ ನೀಡಿದರು? ನೀವು ಅವರ ಸಲಹೆಯನ್ನು ಒಪ್ಪಿಕೊಂಡಿರ? ಯಾಕೆ ? ಅಥವಾ ಯಾಕೆ ಒಪ್ಪಿಕೊಳ್ಳಲಿಲ್ಲ?]

Question 47: Did anyone else from your community think you should have handled the problem in a different way? ಮಗುವಿನ ಈ ಸಮಸ್ಯೆಯನ್ನು ನೀವು ಬೇರೆ ರೀತಿಯಲ್ಲಿ ನಿಭಾಯಿಸಬೇಕಿತ್ತು ಅಂತ ನಿಮ್ಮ ಪರಿಚಯವದರು ಏನಾದ್ರೂ ಹೇಳಿದ್ರಾ?

- [PROBES: what did they advise? Did you take their advice? Why or why not? ಅವರು ಏನೆಂದು ಸಲಹೆ ನೀಡಿದರು? ನೀವು ಅವರ ಸಲಹೆಯನ್ನು ಒಪ್ಪಿಕೊಂಡಿರ? ಯಾಕೆ ? ಅಥವಾ ಯಾಕೆ ಒಪ್ಪಿಕೊಳ್ಳಲಿಲ್ಲ?]

Closing remarks: ಮುಕ್ತಾಯದ ಮಾತುಗಳು:

ನಾನು ಕೇಳಬೇಕಾದ ಪ್ರಶ್ನೆಗಳೆಲ್ಲಾ ಆಯಿತು. ಈ ಗುಂಪು ಕ್ಲಾಸ್ ಬಗ್ಗೆ ಅಥವಾ ನೀವು ನಿಮ್ಮ ಮಗುವನ್ನು ಹೇಗೆ ನೋಡಿಕೊಳ್ಳುತ್ತಿದ್ದೀರಿ ಎನ್ನುವ ಬಗ್ಗೆ ಏನಾದರೂ ನನಗೆ ಹೇಳುವುದು ಇದೆಯಾ?

Is there anything else that you think is important for me to know?

ಇನ್ಯಾವುದಾದರೂ ಮುಖ್ಯವಾದ ವಿಷಯ ನೀವು ನನಗೆ ಹೇಳುತ್ತೀರಾ?

Thank you for speaking with me today.

ಇವತ್ತು ನನ್ನೊಂದಿಗೆ ಮಾತನಾಡಿದ್ದಕ್ಕಾಗಿ ಧನ್ಯವಾದಗಳು.
