# Supplementary material for: Qualitative assessment of family caregiver-centered neonatal education program in Karnataka, India
Source: PLOS Glob Public Health. 2023 Feb 14;3(2):e0000524. doi: 10.1371/journal.pgph.0000524 (PMC10022017; doi:10.1371/journal.pgph.0000524)
Supplement: S4 Text — (PDF) [file pgph.0000524.s004.pdf]

Qualitative Data Analysis: Codebook

Theme	Code ID	Description
Positive perceptions of the CCP session	CCP_pos_perc	Includes any positive perceptions of the CCP class from participants, for example what the participant liked about the CCP session. Does not include negative perceptions of the CCP class.
Negative perceptions of the CCP session	CCP_neg_perc	Includes any negative perceptions of the CCP class from participants, for example what the participant disliked about the CCP session. Does not include positive perceptions of the CCP class.
Usefulness of the CCP session	CCP_use	Includes usefulness of session, for example what was useful or helpful about the session; what was not useful about the session, how participant has used information from class, utility of the CPP. Also includes any descriptions or examples of the utility of having a caregiver at the session.
Worry or concern before the CCP session	worry_before	Any description of the extent to which there was worry or concern prior to the session. Includes if the participant is or is not worried.
Worry or concern after the CCP session	worry_after	Any description of the worry or concern after the session. Includes any increase, decrease or no change in worry after the session.
Other data/quotes about CCP	CCP_other	Any data or quotes about the CCP session that do not fit into the codes listed above.<br>Including suggestions for improving the CCP
Attitude (will be double coded with corresponding behavior)	Attitude	Attitudes, beliefs, social and cultural norms that exist for around a newborn or maternal health behavior or practice. This includes any value judgements and descriptions of the value of doing a particular behavior or practice
Knowledge (will be double coded with corresponding behavior)	Knowledge	Any knowledge, comprehension, or understanding around a newborn or maternal health behavior or practice.<br>May include knowledge acquired through practice and education; awareness or familiarity gained by experience, or specific source of knowledge

Confidence (will be double coded with corresponding behavior)	Confidence	Any description of confidence and comfort with a newborn or maternal health behavior or practice.<br>- Pay attention to participant response to “How do you feel…” questions
Influence of CCP on participant knowledge, behavior or practice	Parent code: CCP_influence<br><br>Child codes:<br>-Behavior<br>-Knowledge	Any mention of if and how the CCP session has influenced a newborn or maternal health behavior /practice and/or knowledge
Other source of influence on knowledge, behavior or practice	behav_influ_other	Any data or quotes that do not fit into the themes listed above about behavior<br>This also includes any other description of influence on behavior/practice including prior experience with newborns
Other trainings	train_other	Any description of another educational training session that the participant attended
CCP materials	CCP_materials	Any description of materials from the CCP group session, including Whats app, Brochures, Videos, Dolls
Barrier of a behavior or practice	Barrier	Things that make it hard to practice/follow a particular newborn or maternal health behavior or practice.
Facilitator of a behavior or practice	Facilitator	Things that make it easy to practice/follow a particular newborn or maternal health behavior or practice.
Perceived benefits of behavior or practice	Benefits	Include any perceived benefits of any newborn or maternal health behavior or practice described by the participant
Perceived risks or a behavior or practice	Risks	Perceived risks of any newborn or maternal health behavior or practice.
Access issues	Access_resources	Availability of materials that allow or enable a particular behavior or practice<br><br>Any mention of access or lack of access to water, food, medicine etc.
	Access_care_barrier	Description of barriers related to accessing care, including cost, distance, geography, gender, culture etc.

	Access_care_avail	Description of the availability of healthcare providers and services
	Access_care_qua	Description of quality of healthcare providers and services
	Access_care_other	Any data or quotes that do not fit into the themes listed above about the care
Breastfeeding	Breastfeeding	Any description of breastfeeding behaviors families perform, currently or in the past. Examples include the following:<br>● Exclusive Breast milk<br>● Breast milk with occasional complementary feeding (list items)
Other foods or liquids	Other_feeding	Feeds baby other items (besides or in addition to breast milk)<br><br>Includes if they say "no" to any "other foods/liquids" and include a description of why they don't feed their baby "other foods"
Handwashing	Handwashing	Any description of handwashing
Cord Care	Cord care	Description of cord care practice which includes both exclusive dry cord care or non-dry cord care.
Kangaroo Mother Care	KMC	Description of KMC, whether it is participants own interpretation or the practice or the version taught in the CCP: contact where newborn baby is kept chest-to-chest and skin-to-skin with a parent or family member
Restrictive diet	Restrictive diet	Description of mother's diet behavior, including foods or liquids avoided or purposely eaten, mother's daily diet, frequency of eating food or restricting diet or skipping meals.
Burping	Burping	Any description of burping
Other newborn health or maternal	Other_behavior	Any data or quotes that do not fit into the themes listed above about behavior

behaviors or practice		
Family member interjection	Fam_interjection	Any time a family member interjects with their own thoughts and opinions during the interview . This includes any time a participant asks a family member to help clarify an interview question.
Newborn complication	Nb_Complication	Any description of a newborn complication
Maternal complication	Maternal_complication	Any description of a maternal complication
Danger signs	Parent code: Danger_sign<br><br>Child codes:<br>-CCP_influence<br>-Other_influence	Description of if and how CCP or another source helped/ did not help participant recognize the problem
Health seeking behavior	Parent code: Health_seeking<br><br>Child codes:<br>-CPP_influence<br>-Other_influence	Description of health seeking behavior from participants. Includes description of how CCP session or another source helped/ did not help participant know what to do to help the problem (call doctor, go to hospital, etc.)
Symptom management to help reduce or eliminate the problem during newborn complication	sick_action	Description of action that was taken to deal with the problem (e.g. medicine given to baby). Includes any challenges or barriers associated with taking action.
Other advice the participant receives regarding a behavior or practice AND to help reduce or eliminate the	advice_source	Any description of advice source (other class, friends, family etc.), also includes participant description of source of advice on how to deal with health complication
	advice _type	Any description of advice given to participant regarding behavior/practice and/or how to deal with a complication

newborn complication<br><br>(Advice other than what is learned in CCP)		
Quotes for external use	Quotes_public	Any quotes that are good for publication and external communication
Any translation issues	Translation	Any areas that we need translated again
